# Supplementary material for: Synergy between Variant PRC1 Complexes Defines Polycomb-Mediated Gene Repression
Source: Mol Cell. 2019 Jun 6;74(5):1020–1036.e8. doi: 10.1016/j.molcel.2019.03.024 (PMC6561741; doi:10.1016/j.molcel.2019.03.024)
Supplement: Document S1. Figures S1–S7 [file mmc1.pdf]

Molecular Cell, Volume *74*

Supplemental Information

Synergy between Variant PRC1 Complexes Defines Polycomb-Mediated Gene Repression

Nadezda A. Fursova, Neil P. Blackledge, Manabu Nakayama, Shinsuke Ito, Yoko Koseki, Anca M. Farcas, Hamish W. King, Haruhiko Koseki, and Robert J. Klose

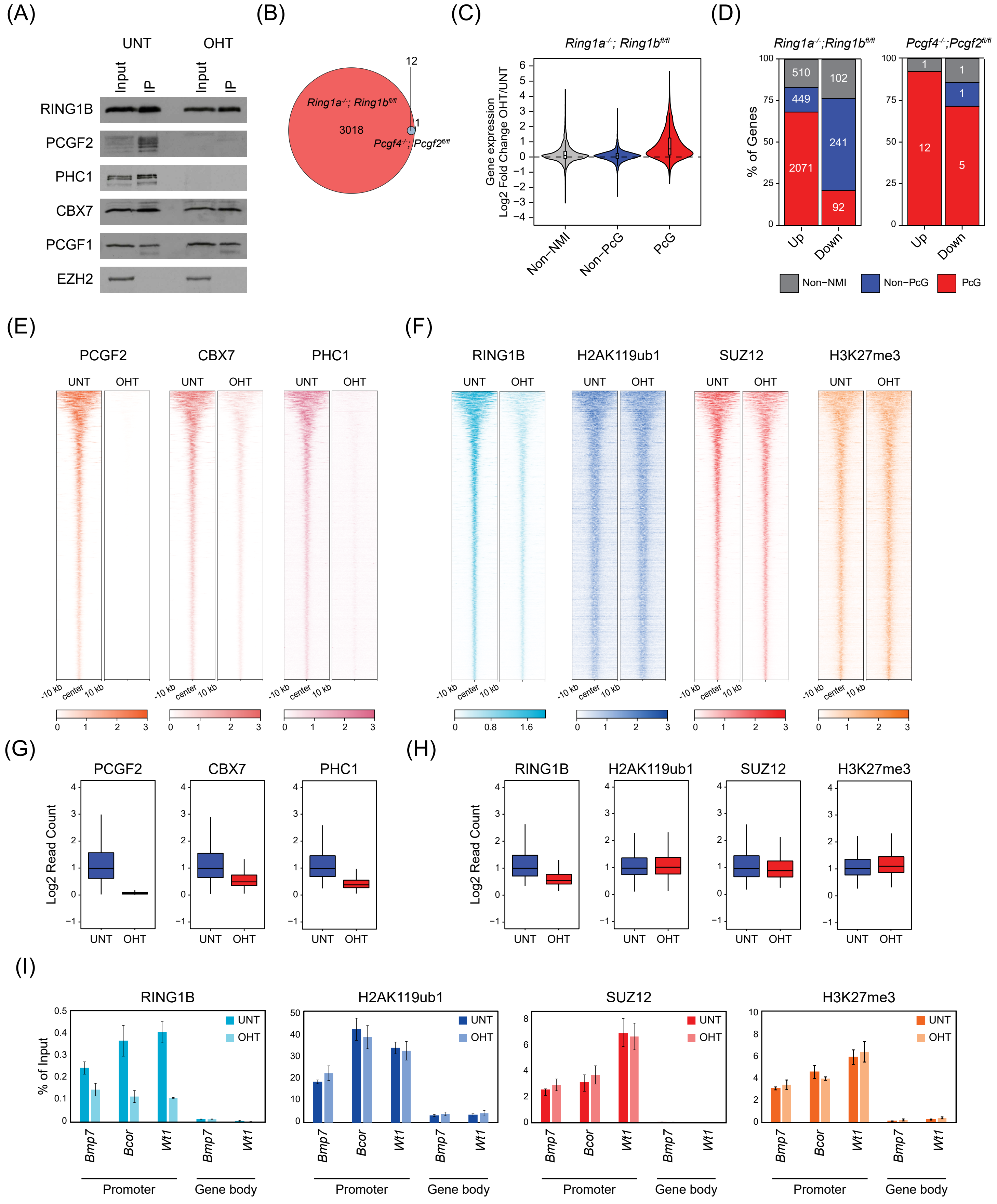
(A)
UNT
OHT
Input
IP
Input
IP
RING1B
PCGF2
PHC1
CBX7
PCGF1
EZH2
(B)
12
Ring1a-/-; Ring1bfl/fl
3018
1
Pcgf4-/-; Pcgf2fl/fl
(C)
Ring1a-/-; Ring1bfl/fl
Gene expression
Log2 Fold Change OHT/UNT
Non−NMI
Non−PcG
PcG
(D)
Ring1a-/-;Ring1bfl/fl
Pcgf4-/-;Pcgf2fl/fl
% of Genes
510
449
2071
102
241
92
1
12
1
1
5
Up
Down
Non−NMI
Non−PcG
PcG
(E)
PCGF2
CBX7
PHC1
UNT
OHT
-10 kb
center
10 kb
(F)
RING1B
H2AK119ub1
SUZ12
H3K27me3
(G)
Log2 Read Count
(H)
(I)
% of Input
Bmp7
Bcor
Wt1
Promoter
Gene body

Figure S1

Figure S1. Related to Figure 1.

- **(A)** Input and immunoprecipitation of RING1B from *Pcgf4$^{-/-}$;Pcgf2$^{fl/fl}$* ESCs (UNT and OHT-treated) followed by western blot with antibodies against PCGF2, PHC1 and CBX7 (canonical PRC1-specific subunits), PCGF1 (a variant PRC1-specific factor) and EZH2 (a PRC2 component that does not interact with RING1B). In OHT-treated cells, PHC1 and PCGF2 no longer associate with RING1B, indicating canonical PRC1 has been disrupted.
- **(B)** A Venn diagram of significantly upregulated genes (p-adj < 0.05 and > 1.5-fold) following OHT treatment in *Ring1a$^{-/-}$;Ring1b$^{fl/fl}$* and *Pcgf4$^{-/-}$;Pcgf2$^{fl/fl}$* ESCs.
- **(C)** A violin plot comparing log2-fold expression changes in *Ring1a$^{-/-}$;Ring1b$^{fl/fl}$* ESCs for three classes of genes - Non-NMI (n = 5867), Non-PcG-occupied (n = 9898) and PcG-occupied (n = 4868). Non-NMI genes lack a non-methylated CpG island (NMI) at their promoter. PcG- and Non-PcG-occupied genes have an NMI promoter that is either bound or not bound by Polycomb complexes.
- **(D)** Bar plots of the distribution of gene expression changes (p-adj < 0.05 and > 1.5-fold) in *Ring1a$^{-/-}$;Ring1b$^{fl/fl}$* and *Pcgf4$^{-/-}$;Pcgf2$^{fl/fl}$* ESCs following OHT treatment between different classes of genes described in (C).
- **(E)** Heatmaps of PCGF2, CBX7 and PHC1 cChIP-seq in *Pcgf4$^{-/-}$;Pcgf2$^{fl/fl}$* ESCs (UNT and OHT) at classical Polycomb chromatin domains sorted based on RING1B occupancy in untreated cells.
- **(F)** As in (E) for RING1B, H2AK119ub1, SUZ12 and H3K27me3.
- **(G)** Box plots of PCGF2, CBX7 and PHC1 cChIP-seq signal at classical Polycomb chromatin domains in *Pcgf4$^{-/-}$; Pcgf2$^{fl/fl}$* ESCs before (UNT, blue) and after OHT treatment (OHT, red).
- **(H)** As in (G) for RING1B, H2AK119ub1, SUZ12 and H3K27me3.
- **(I)** ChIP-qPCR analysis of RING1B, H2AK119ub1, SUZ12 and H3K27me3 in *Pcgf4$^{-/-}$;Pcgf2$^{fl/fl}$* ESCs (UNT and OHT) for a panel of Polycomb-bound genes. Error bars show SEM (n=2 for RING1B and n=3 for H2AK119ub1, SUZ12 and H3K27me3).

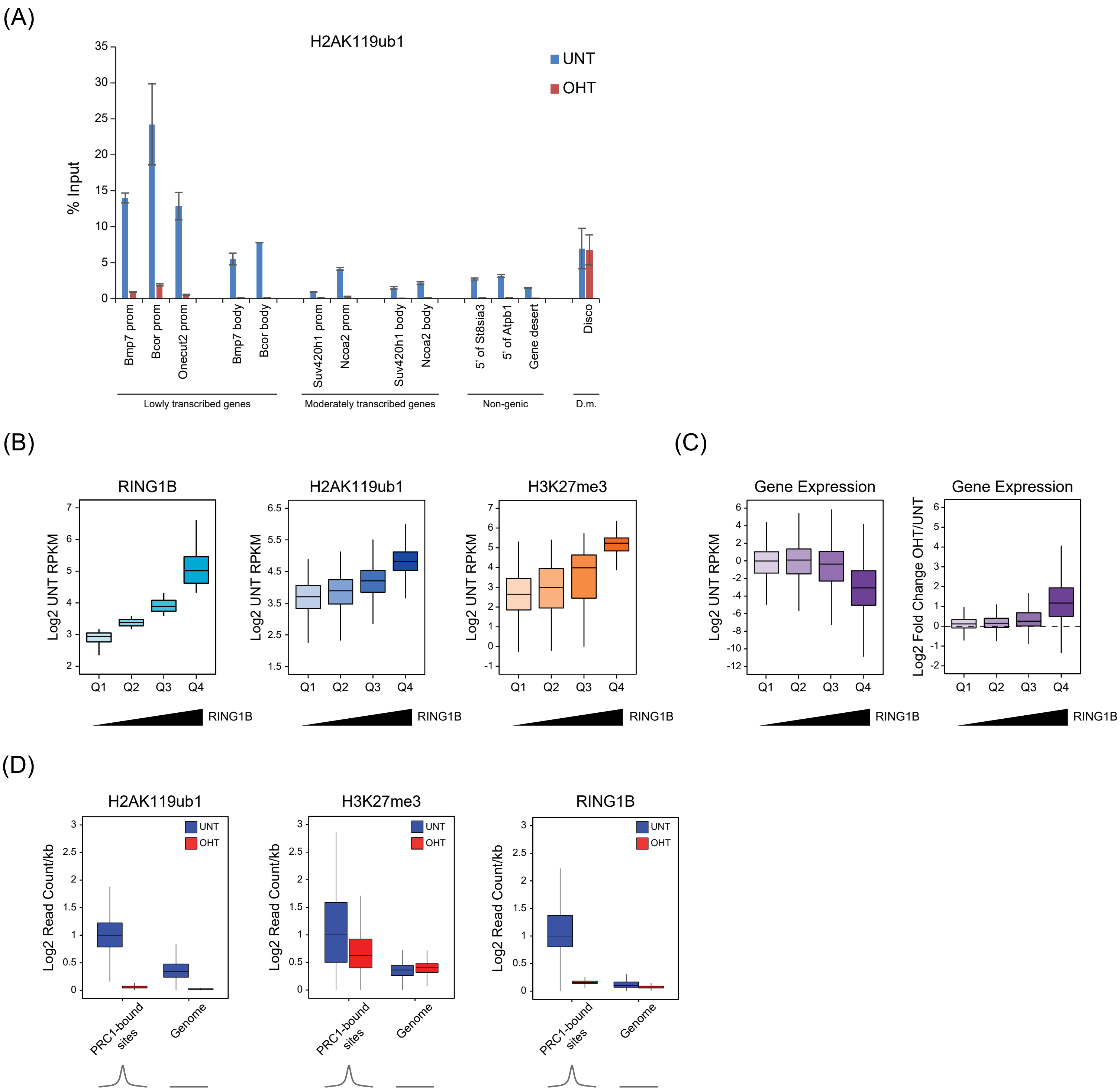
(A)
H2AK119ub1
UNT
OHT
% Input
Bmp7 prom
Bcor prom
Onecut2 prom
Bmp7 body
Bcor body
Suv420h1 prom
Ncoa2 prom
Suv420h1 body
Ncoa2 body
5' of St8sia3
5' of Atpb1
Gene desert
Disco
Lowly transcribed genes
Moderately transcribed genes
Non-genic
D.m.
(B)
RING1B
H2AK119ub1
H3K27me3
Log2 UNT RPKM
Q1
Q2
Q3
Q4
RING1B
(C)
Gene Expression
Log2 UNT RPKM
Log2 Fold Change OHT/UNT
(D)
H2AK119ub1
H3K27me3
RING1B
Log2 Read Count/kb
UNT
OHT
PRC1-bound sites
Genome

Figure S2

Figure S2. Related to Figure 2.

- **(A)** ChIP-qPCR analysis of H2AK119ub1 in untreated (UNT, blue) and OHT-treated (OHT, red) *Ring1a*$^{-/-}$;*Ring1b*$^{fl/fl}$ ESCs. To illustrate the broad nature of H2AK119ub1 in mouse ESCs, qPCR was performed using primers at various genic (lowly or moderately transcribed) or non-genic regions as indicated. As a control for ChIP efficiency, all ChIPs included Drosophila SG4 cells, which were interrogated using primers specific for a Polycomb target in *Drosophila melanogaster* (D.m.).
- **(B)** Box plots of RING1B, H2AK119ub1 and H3K27me3 cChIP-seq signal in untreated *Ring1a*$^{-/-}$; *Ring1b*$^{fl/fl}$ ESCs at PRC1-bound gene promoters divided into quartiles based on RING1B occupancy (from lowest Q1 to highest Q4).
- **(C)** Box plots of gene expression in untreated cells (left panel) and log2-fold expression changes following OHT treatment (right panel) in *Ring1a*$^{-/-}$;*Ring1b*$^{fl/fl}$ ESCs for the gene quartiles as in (B).
- **(D)** Box plots of H2AK119ub1, H3K27me3 and RING1B cChIP-seq signal at PRC1-bound sites and 100 kb windows covering the genome in *Ring1a*$^{-/-}$;*Ring1b*$^{fl/fl}$ ESCs (UNT, blue, and OHT, red). This analysis shows that there is an appreciable amount of H2AK119ub1 outside of PRC1-bound sites throughout the genome which is lost after removal of RING1A/B. In addition, it demonstrates that PRC1 loss leads to a specific reduction in H3K27me3 at regions with punctate H2AK119ub1, indicating that communication between PRC1 and PRC2 is largely restricted to these regions of the genome.

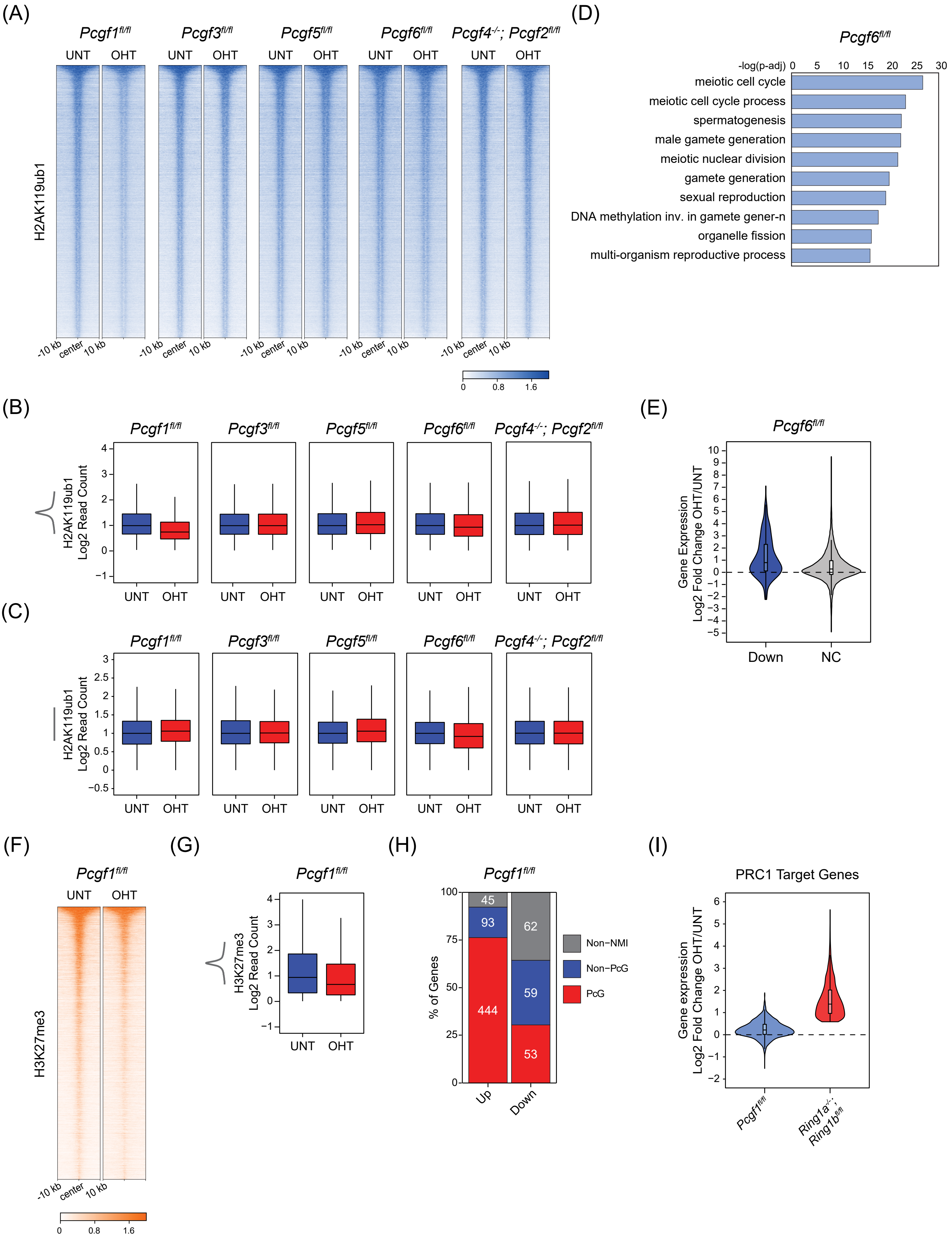
(A)
Pcgf1fl/fl
Pcgf3fl/fl
Pcgf5fl/fl
Pcgf6fl/fl
Pcgf4-/-; Pcgf2fl/fl
UNT
OHT
H2AK119ub1
-10 kb
center
10 kb
0
0.8
1.6
(B)
H2AK119ub1 Log2 Read Count
(C)
(D)
-log(p-adj)
meiotic cell cycle
meiotic cell cycle process
spermatogenesis
male gamete generation
meiotic nuclear division
gamete generation
sexual reproduction
DNA methylation inv. in gamete gener-n
organelle fission
multi-organism reproductive process
(E)
Gene Expression Log2 Fold Change OHT/UNT
Down
NC
(F)
H3K27me3
(G)
H3K27me3 Log2 Read Count
(H)
% of Genes
45
93
444
62
59
53
Up
Down
Non−NMI
Non−PcG
PcG
(I)
PRC1 Target Genes
Gene expression Log2 Fold Change OHT/UNT
Ring1a-/-; Ring1bfl/fl

Figure S3

Figure S3. Related to Figure 3.

- **(A)** Heatmaps of H2AK119ub1 cChIP-seq in *Pcgf1*$^{fl/fl}$, *Pcgf3*$^{fl/fl}$, *Pcgf5*$^{fl/fl}$, *Pcgf6*$^{fl/fl}$ and *Pcgf4*$^{-/-}$*; Pcgf2*$^{fl/fl}$ ESCs (UNT and OHT) at PRC1-bound sites sorted based on RING1B occupancy in untreated *Ring1a*$^{-/-}$*;Ring1b*$^{fl/fl}$ ESCs.
- **(B)** Box plots of H2AK119ub1 cChIP-seq at PRC1-bound sites in *Pcgf1*$^{fl/fl}$, *Pcgf3*$^{fl/fl}$, *Pcgf5*$^{fl/fl}$, *Pcgf6*$^{fl/fl}$ and *Pcgf4*$^{-/-}$*;Pcgf2*$^{fl/fl}$ ESCs before (UNT, blue) and after OHT treatment (OHT, red).
- **(C)** Box plots of H2AK119ub1 cChIP-seq at 100 kb windows covering the genome in *Pcgf1*$^{fl/fl}$, *Pcgf3*$^{fl/fl}$, *Pcgf5*$^{fl/fl}$, *Pcgf6*$^{fl/fl}$ and *Pcgf4*$^{-/-}$*;Pcgf2*$^{fl/fl}$ ESCs (UNT and OHT).
- **(D)** A gene ontology analysis of Biological Process term enrichment for genes showing statistically significant reductions in H2AK119ub1 levels at their promoters in *Pcgf6*$^{fl/fl}$ ESCs following OHT treatment (n = 241).
- **(E)** A violin plot comparing log2-fold expression changes for genes showing a statistically significant reduction (Down) or no change (NC) in H2AK119ub1 levels at their promoters in *Pcgf6*$^{fl/fl}$ ESCs following OHT treatment.
- **(F)** A heatmap of H3K27me3 cChIP-seq signal in *Pcgf1*$^{fl/fl}$ ESCs (UNT and OHT) at PRC1-bound sites sorted based on RING1B occupancy in untreated *Ring1a*$^{-/-}$*;Ring1b*$^{fl/fl}$ ESCs.
- **(G)** A box plot of H3K27me3 cChIP-seq signal at PRC1-bound sites in *Pcgf1*$^{fl/fl}$ ESCs before (UNT, blue) and after OHT treatment (OHT, red).
- **(H)** A bar plot of the distribution of gene expression changes (p-adj < 0.05 and > 1.5-fold) in *Pcgf1*$^{fl/fl}$ ESCs following OHT treatment between different classes of genes (Non-NMI, Non-PcG, PcG) described in Figure S1C.
- **(I)** A violin plot comparing log2-fold expression changes of PRC1 target genes in *Pcgf1*$^{fl/fl}$ and *Ring1a*$^{-/-}$*;Ring1b*$^{fl/fl}$ ESCs following OHT treatment.

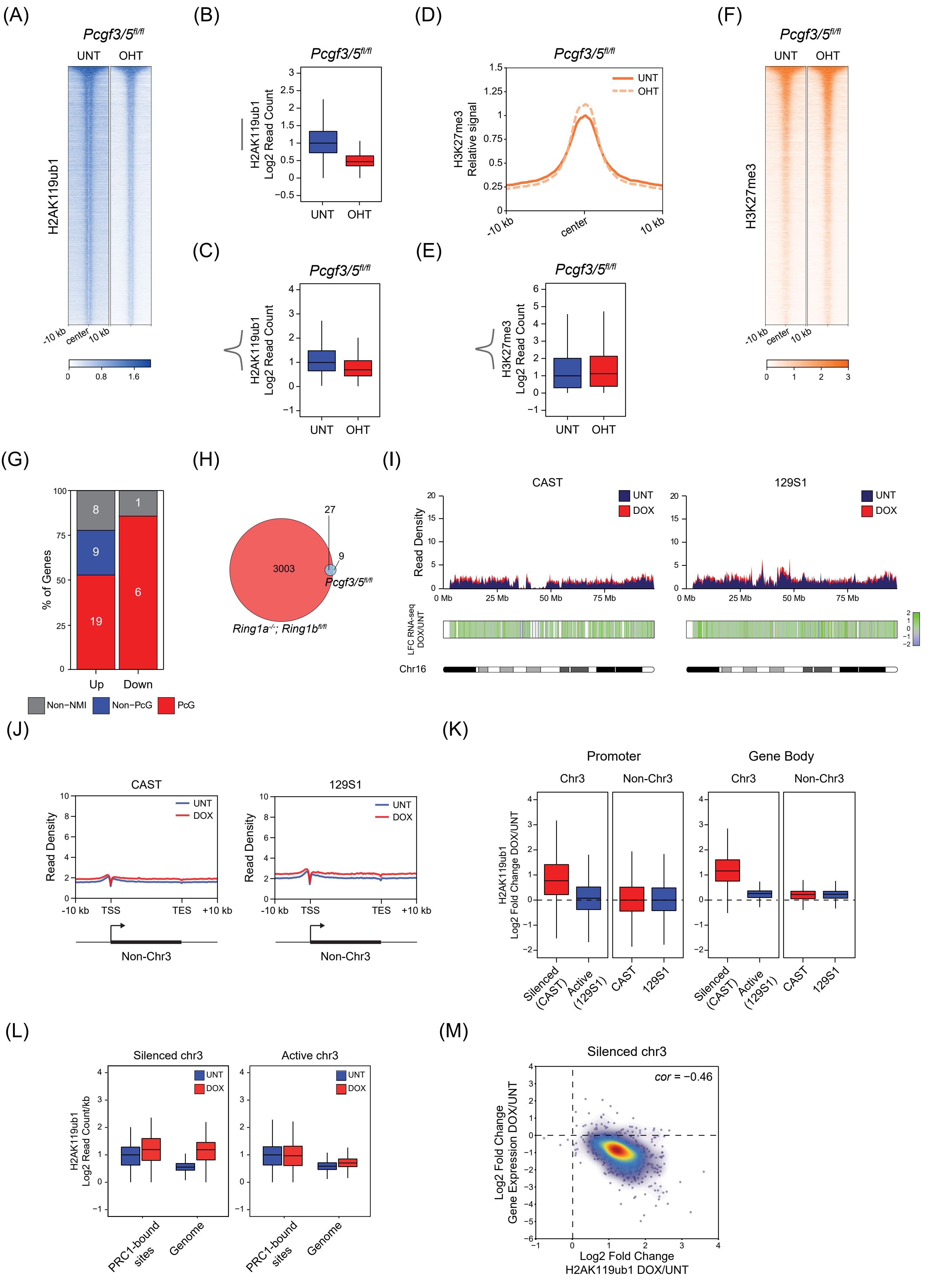
(A)
Pcgf3/5fl/fl
UNT
OHT
H2AK119ub1
-10 kb
center
10 kb
0
0.8
1.6
(B)
Pcgf3/5fl/fl
H2AK119ub1
Log2 Read Count
UNT
OHT
(C)
Pcgf3/5fl/fl
H2AK119ub1
Log2 Read Count
UNT
OHT
(D)
Pcgf3/5fl/fl
H3K27me3
Relative signal
UNT
OHT
-10 kb
center
10 kb
(E)
Pcgf3/5fl/fl
H3K27me3
Log2 Read Count
UNT
OHT
(F)
Pcgf3/5fl/fl
UNT
OHT
H3K27me3
-10 kb
center
10 kb
0
1
2
3
(G)
% of Genes
8
9
19
1
6
Up
Down
Non−NMI
Non−PcG
PcG
(H)
3003
27
9
Pcgf3/5fl/fl
Ring1a-/-; Ring1bfl/fl
(I)
CAST
129S1
Read Density
UNT
DOX
0 Mb
25 Mb
50 Mb
75 Mb
LFC RNA-seq DOX/UNT
Chr16
(J)
CAST
129S1
Read Density
UNT
DOX
-10 kb
TSS
TES
+10 kb
Non-Chr3
(K)
Promoter
Gene Body
Chr3
Non-Chr3
H2AK119ub1
Log2 Fold Change DOX/UNT
Silenced (CAST)
Active (129S1)
CAST
129S1
(L)
Silenced chr3
Active chr3
H2AK119ub1
Log2 Read Count/kb
UNT
DOX
PRC1-bound sites
Genome
(M)
Silenced chr3
cor = −0.46
Log2 Fold Change
Gene Expression DOX/UNT
Log2 Fold Change
H2AK119ub1 DOX/UNT

Figure S4

Figure S4. Related to Figure 4.

(A) A heatmap of H2AK119ub1 cChIP-seq in *Pcgf3/5$^{fl/fl}$* ESCs (UNT and OHT) at PRC1-bound sites sorted based on RING1B occupancy in untreated *Ring1a$^{-/-}$;Ring1b$^{fl/fl}$* ESCs.

(B) A box plot of H2AK119ub1 cChIP-seq signal at 100 kb windows covering the genome in *Pcgf3/5$^{fl/fl}$* ESCs before (UNT, blue) and after OHT treatment (OHT, red).

(C) A box plot of H2AK119ub1 cChIP-seq signal at PRC1-bound sites in *Pcgf3/5$^{fl/fl}$* ESCs before (UNT, blue) and after OHT treatment (OHT, red).

(D) A metaplot of H3K27me3 cChIP-seq signal at PRC1-bound sites in *Pcgf3/5$^{fl/fl}$* ESCs (UNT and OHT).

(E) A box plot of H3K27me3 cChIP-seq signal at PRC1-bound sites in *Pcgf3/5$^{fl/fl}$* ESCs before (UNT, blue) and after OHT treatment (OHT, red).

(F) A heatmap of H3K27me3 cChIP-seq in *Pcgf3/5$^{fl/fl}$* ESCs (UNT and OHT) at PRC1-bound sites sorted based on RING1B occupancy in untreated *Ring1a$^{-/-}$;Ring1b$^{fl/fl}$* ESCs.

(G) A bar plot of the distribution of gene expression changes (p-adj < 0.05 and > 1.5-fold) in *Pcgf3/5$^{fl/fl}$* ESCs following OHT treatment between different classes of genes (Non-NMI, Non-PcG, PcG) described in Figure S1C.

(H) A Venn diagram of significantly upregulated genes (p-adj < 0.05 and > 1.5-fold) in *Pcgf3/5$^{fl/fl}$* and *Ring1a$^{-/-}$;Ring1b$^{fl/fl}$* ESCs following OHT treatment.

(I) Allele-specific chromosome density plots for H2AK119ub1 cChIP-seq across a control chromosome lacking the *Xist* transgene (chromosome 16) in the *Mus domesticus* (129S1) x *Mus castaneus* F1 hybrid ESCs (UNT and DOX). Below is a heatmap of corresponding log2-fold gene expression changes upon DOX addition. The bins containing no expressed genes are shown in white.

(J) Allele-specific metaplots of H2AK119ub1 cChIP-seq for genes located on chromosomes other than chromosome 3 in the *Mus domesticus* (129S1) x *Mus castaneus* F1 hybrid ESCs (UNT and DOX). Genes were scaled to the same length and aligned at their TSS and TES.

(K) Allele-specific box plots of log2-fold changes in H2AK119ub1 cChIP-seq following DOX treatment at promoters (TSS ± 500 bp) or bodies of genes located on chromosome 3 (Chr3) or any other chromosome (Non-Chr3) in the *Mus domesticus* (129S1) x *Mus castaneus* F1 hybrid ESCs.

(L) Allele-specific box plots of H2AK119ub1 cChIP-seq signal at PRC1-bound sites and 100 kb windows covering the chromosome for the silenced (left, from *Mus castaneus*) or active (right, from *Mus domesticus*) allele of chromosome 3 in the *Mus domesticus* (129S1) x *Mus castaneus* F1 hybrid ESCs (UNT and DOX).

(M) A scatterplot comparing log2-fold changes in H2AK119ub1 and gene expression for all expressed 250 kb genomic bins across the silenced allele of chromosome 3 following induction of *Xist* transgene expression with DOX. *Cor* denotes a Pearson correlation coefficient.

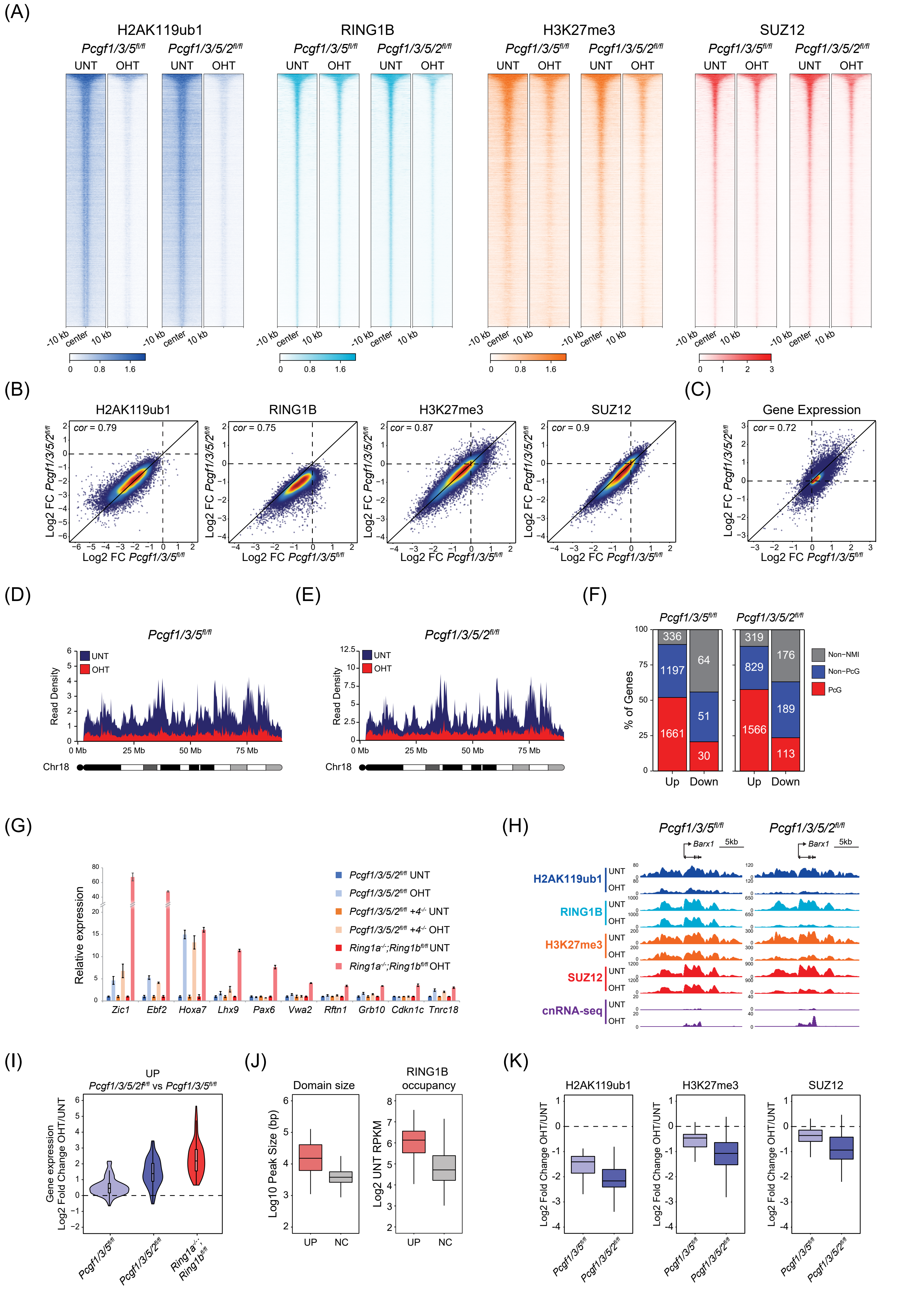
(A)
H2AK119ub1
RING1B
H3K27me3
SUZ12
Pcgf1/3/5fl/fl
Pcgf1/3/5/2fl/fl
UNT
OHT
-10 kb
center
10 kb
(B)
(C)
Gene Expression
cor = 0.79
cor = 0.75
cor = 0.87
cor = 0.9
cor = 0.72
Log2 FC Pcgf1/3/5/2fl/fl
Log2 FC Pcgf1/3/5fl/fl
(D)
(E)
(F)
Read Density
Chr18
0 Mb
25 Mb
50 Mb
75 Mb
% of Genes
Non-NMI
Non-PcG
PcG
Up
Down
336
1197
1661
64
51
30
319
829
1566
176
189
113
(G)
Relative expression
Pcgf1/3/5/2fl/fl UNT
Pcgf1/3/5/2fl/fl OHT
Pcgf1/3/5/2fl/fl +4-/- UNT
Pcgf1/3/5/2fl/fl +4-/- OHT
Ring1a-/-;Ring1bfl/fl UNT
Ring1a-/-;Ring1bfl/fl OHT
Zic1
Ebf2
Hoxa7
Lhx9
Pax6
Vwa2
Rftn1
Grb10
Cdkn1c
Tnrc18
(H)
Barx1
5kb
cnRNA-seq
(I)
UP
Pcgf1/3/5/2fl/fl vs Pcgf1/3/5fl/fl
Gene expression
Log2 Fold Change OHT/UNT
Ring1a-/-; Ring1bfl/fl
(J)
Domain size
RING1B occupancy
Log10 Peak Size (bp)
Log2 UNT RPKM
UP
NC
(K)
Log2 Fold Change OHT/UNT

Figure S5

Figure S5. Related to Figure 5.

(A) Heatmaps of H2AK119ub1, RING1B, H3K27me3 and SUZ12 cChIP-seq in *Pcgf1/3/5*$^{fl/fl}$ (*left*) and *Pcgf1/3/5/2*$^{fl/fl}$ (*right*) ESCs (UNT and OHT) at PRC1-bound sites sorted based on RING1B occupancy in untreated *Ring1a*$^{-/-}$;*Ring1b*$^{fl/fl}$ ESCs.

(B) Scatterplots comparing log2-fold changes in H2AK119ub1, RING1B, H3K27me3 and SUZ12 cChIP-seq at PRC1-bound sites between *Pcgf1/3/5*$^{fl/fl}$ and *Pcgf1/3/5/2*$^{fl/fl}$ ESCs following OHT treatment. *Cor* denotes a Pearson correlation coefficient.

(C) A scatterplot comparing log2-fold changes in gene expression (cnRNA-seq) between *Pcgf1/3/5*$^{fl/fl}$ and *Pcgf1/3/5/2*$^{fl/fl}$ ESCs following OHT treatment. *Cor* denotes a Pearson correlation coefficient.

(D) A chromosome density plot of H2AK119ub1 cChIP-seq across chromosome 18 in *Pcgf1/3/5*$^{fl/fl}$ ESCs before (UNT, blue) and after OHT treatment (OHT, red).

(E) As in (D) for *Pcgf1/3/5/2*$^{fl/fl}$ ESCs.

(F) A bar plot comparing the distribution of gene expression changes (p-adj < 0.05 and > 1.5-fold) in *Pcgf1/3/5*$^{fl/fl}$ (left) and *Pcgf1/3/5/2*$^{fl/fl}$ (right) ESCs following OHT treatment between different classes of genes (Non-NMI, Non-PcG, PcG) described in Figure S1C.

(G) RT-qPCR analysis of expression for a panel of Polycomb target genes in *Pcgf1/3/5/2*$^{fl/fl}$, *Pcgf4*$^{-/-}$;*Pcgf1/3/5/2*$^{fl/fl}$ and *Ring1a*$^{-/-}$;*Ring1b*$^{fl/fl}$ ESCs (UNT and OHT). Gene expression is normalised to *b-actin* and is shown relative to the average expression in untreated cells for each individual cell line. Error bars show SEM (n=3).

(H) A genomic snapshot of a PRC1 target gene that is further derepressed when PCGF2 is removed in addition to PCGF1/3/5, showing PRC1 (H2AK119ub1 and RING1B) and PRC2 (H3K27me3 and SUZ12) cChIP-seq, and cnRNA-seq in *Pcgf1/3/5*$^{fl/fl}$ and *Pcgf1/3/5/2*$^{fl/fl}$ ESCs (UNT and OHT).

(I) A violin plot comparing log2-fold expression changes for PRC1 target genes that are further derepressed when PCGF2 is removed along with PCGF1/3/5 (n = 137) in *Pcgf1/3/5*$^{fl/fl}$, *Pcgf1/3/5/2*$^{fl/fl}$ and *Ring1a*$^{-/-}$;*Ring1b*$^{fl/fl}$ ESCs following OHT treatment.

(J) Box plots of the size (left panel) and RING1B occupancy in untreated cells (right panel) for Polycomb chromatin domains associated with PRC1 target genes displaying an additional derepression (UP, n=137) or no additional derepression (NC, n = 1934) when PCGF2 is removed along with PCGF1/3/5 in *Pcgf1/3/5/2*$^{fl/fl}$ ESCs.

(K) Box plots comparing log2-fold changes in H2AK119ub1, H3K27me3 and SUZ12 cChIP-seq at promoters of PRC1 target genes that are further derepressed when PCGF2 is removed along with PCGF1/3/5 (n = 137) in *Pcgf1/3/5*$^{fl/fl}$ and *Pcgf1/3/5/2*$^{fl/fl}$ ESCs following OHT treatment.

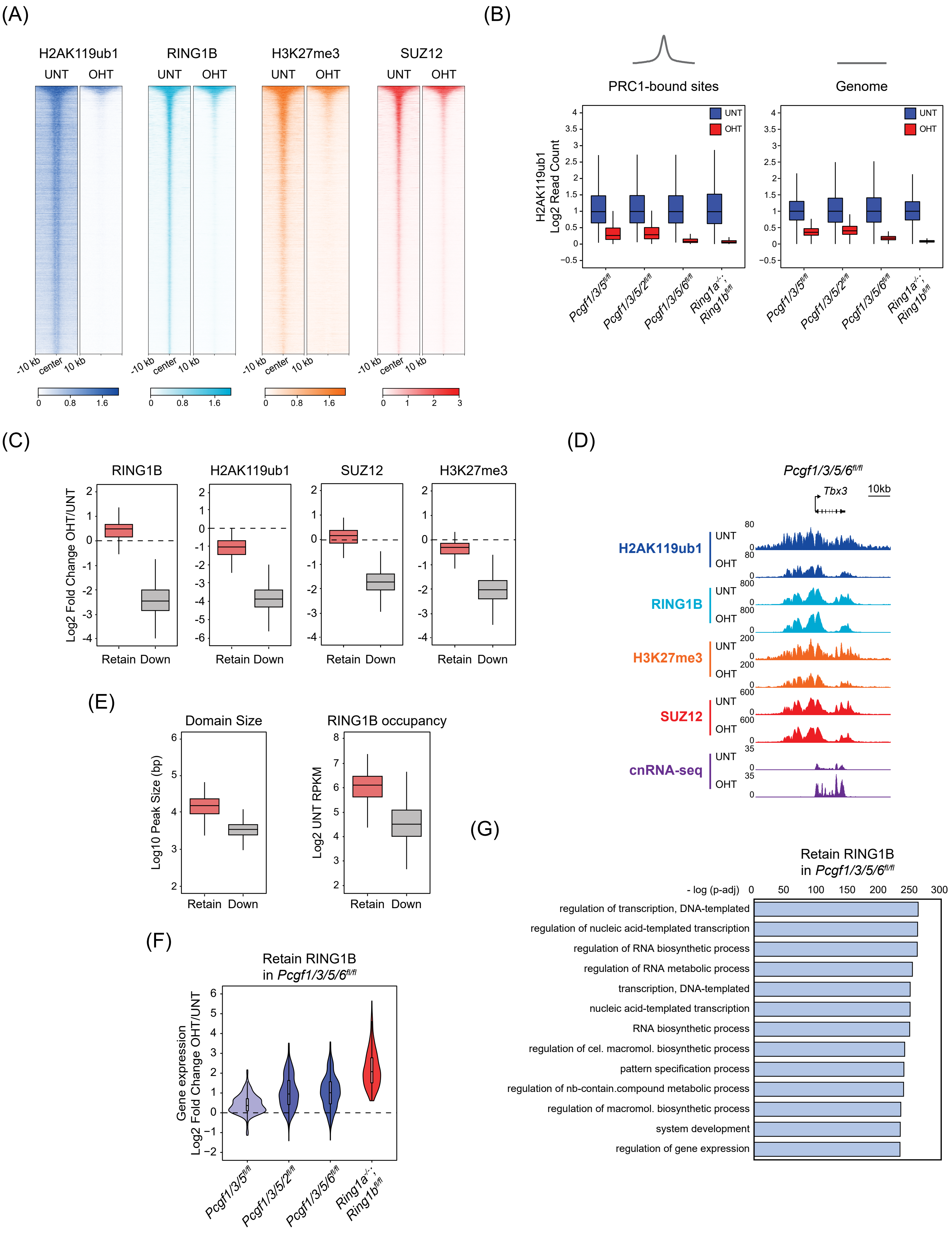

(A)
H2AK119ub1
RING1B
H3K27me3
SUZ12
UNT
OHT
-10 kb
center
10 kb
(B)
PRC1-bound sites
Genome
H2AK119ub1
Log2 Read Count
UNT
OHT
Pcgf1/3/5fl/fl
Pcgf1/3/5/2fl/fl
Pcgf1/3/5/6fl/fl
Ring1a-/-; Ring1bfl/fl
(C)
RING1B
H2AK119ub1
SUZ12
H3K27me3
Log2 Fold Change OHT/UNT
Retain
Down
(D)
Pcgf1/3/5/6fl/fl
Tbx3
10kb
H2AK119ub1
RING1B
H3K27me3
SUZ12
cnRNA-seq
(E)
Domain Size
Log10 Peak Size (bp)
RING1B occupancy
Log2 UNT RPKM
Retain
Down
(F)
Retain RING1B in Pcgf1/3/5/6fl/fl
Gene expression Log2 Fold Change OHT/UNT
Pcgf1/3/5fl/fl
Pcgf1/3/5/2fl/fl
Pcgf1/3/5/6fl/fl
Ring1a-/-; Ring1bfl/fl
(G)
Retain RING1B in Pcgf1/3/5/6fl/fl
- log (p-adj)
regulation of transcription, DNA-templated
regulation of nucleic acid-templated transcription
regulation of RNA biosynthetic process
regulation of RNA metabolic process
transcription, DNA-templated
nucleic acid-templated transcription
RNA biosynthetic process
regulation of cel. macromol. biosynthetic process
pattern specification process
regulation of nb-contain.compound metabolic process
regulation of macromol. biosynthetic process
system development
regulation of gene expression

Figure S6

Figure S6. Related to Figure 6.

- **(A)** Heatmaps of H2AK119ub1, RING1B, H3K27me3 and SUZ12 cChIP-seq in *Pcgf1/3/5/6*$^{fl/fl}$ ESCs (UNT and OHT) at PRC1-bound sites sorted based on RING1B occupancy in untreated *Ring1a*$^{-/-}$*;Ring1b*$^{fl/fl}$ ESCs.
- **(B)** Box plots of H2AK119ub1 cChIP-seq signal at PRC1-bound sites (left) and 100 kb windows covering the genome (right) in *Pcgf1/3/5*$^{fl/fl}$, *Pcgf1/3/5/2*$^{fl/fl}$, *Pcgf1/3/5/6*$^{fl/fl}$ and *Ring1a*$^{-/-}$*; Ring1b*$^{fl/fl}$ ESCs (UNT, blue, and OHT, red).
- **(C)** Box plots of log2-fold changes in RING1B, H2AK119ub1, SUZ12 and H3K27me3 cChIP-seq at the promoters of PRC1 target genes showing no reduction (Retain, n=354) or a statistically significant decrease (Down, n=1717) in RING1B occupancy in *Pcgf1/3/5/6*$^{fl/fl}$ ESCs following OHT treatment.
- **(D)** A genomic snapshot of a PRC1 target gene that retains RING1B binding at the promoter following removal of PCGF1/3/5/6, showing PRC1 (H2AK119ub1 and RING1B) and PRC2 (H3K27me3 and SUZ12) cChIP-seq, and cnRNA-seq in *Pcgf1/3/5/6*$^{fl/fl}$ ESCs (UNT and OHT).
- **(E)** Box plots of the size (left panel) and RING1B occupancy in untreated cells (right panel) for Polycomb chromatin domains associated with the promoters of PRC1 target genes that demonstrate no reduction (Retain, n=354) or a statistically significant decrease (Down, n=1717) in RING1B occupancy in *Pcgf1/3/5/6*$^{fl/fl}$ ESCs following OHT treatment.
- **(F)** A violin plot of log2-fold expression changes for PRC1 target genes that retain RING1B binding at their promoter upon PCGF1/3/5/6 removal (n=354) in *Pcgf1/3/5*$^{fl/fl}$, *Pcgf1/3/5/2*$^{fl/fl}$, *Pcgf1/3/5/6*$^{fl/fl}$, and *Ring1a*$^{-/-}$*;Ring1b*$^{fl/fl}$ ESCs following OHT treatment.
- **(G)** A gene ontology analysis of Biological Process term enrichment for genes that retain RING1B in *Pcgf1/3/5/6*$^{fl/fl}$ ESCs following OHT treatment (n = 354).

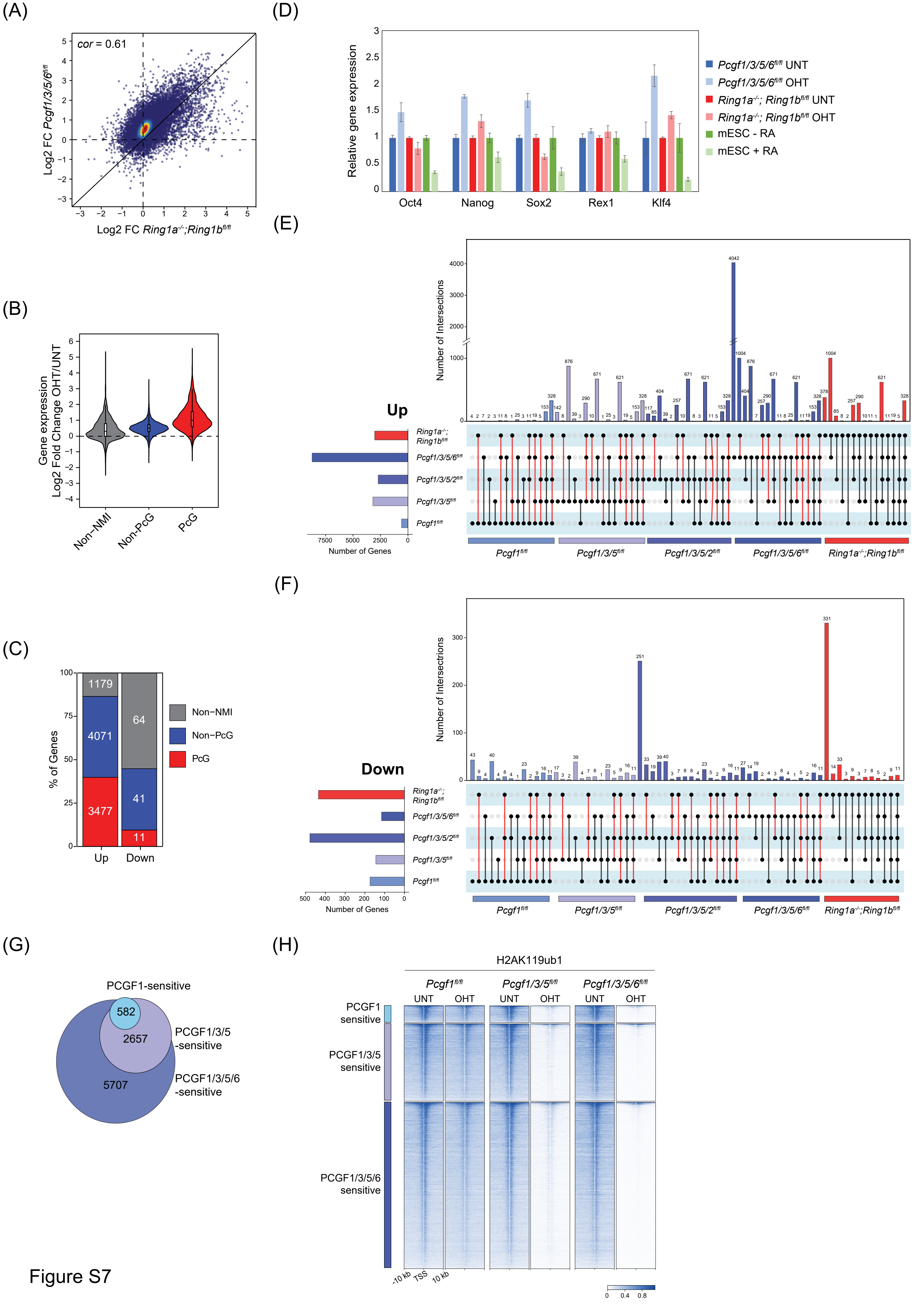

(A)
cor = 0.61
Log2 FC Pcgf1/3/5/6fl/fl
Log2 FC Ring1a-/-;Ring1bfl/fl
(B)
Gene expression
Log2 Fold Change OHT/UNT
Non−NMI
Non−PcG
PcG
(C)
% of Genes
1179
4071
3477
64
41
11
Up
Down
Non−NMI
Non−PcG
PcG
(D)
Relative gene expression
Pcgf1/3/5/6fl/fl UNT
Pcgf1/3/5/6fl/fl OHT
Ring1a-/-; Ring1bfl/fl UNT
Ring1a-/-; Ring1bfl/fl OHT
mESC - RA
mESC + RA
Oct4
Nanog
Sox2
Rex1
Klf4
(E)
Up
Number of Intersections
Number of Genes
Ring1a-/-; Ring1bfl/fl
Pcgf1/3/5/6fl/fl
Pcgf1/3/5/2fl/fl
Pcgf1/3/5fl/fl
Pcgf1fl/fl
(F)
Down
Number of Intersectrions
Number of Genes
Ring1a-/-; Ring1bfl/fl
Pcgf1/3/5/6fl/fl
Pcgf1/3/5/2fl/fl
Pcgf1/3/5fl/fl
Pcgf1fl/fl
(G)
PCGF1-sensitive
582
2657
5707
PCGF1/3/5
-sensitive
PCGF1/3/5/6
-sensitive
(H)
H2AK119ub1
Pcgf1fl/fl
Pcgf1/3/5fl/fl
Pcgf1/3/5/6fl/fl
UNT
OHT
PCGF1
sensitive
PCGF1/3/5
sensitive
PCGF1/3/5/6
sensitive
-10 kb
TSS
10 kb
0
0.4
0.8

Figure S7

Figure S7. Related to Figure 7.

- **(A)** A scatterplot comparing log2-fold changes in gene expression in *Pcgf1/3/5/6$^{fl/fl}$* and *Ring1a$^{-/-}$; Ring1b$^{fl/fl}$* ESCs following OHT treatment. *Cor* denotes a Pearson correlation coefficient.
- **(B)** A violin plot of log2-fold gene expression changes in *Pcgf1/3/5/6$^{fl/fl}$* ESCs following OHT treatment for different classes of genes (Non-NMI, Non-PcG and PcG) described in Figure S1C.
- **(C)** A bar plot of the distribution of gene expression changes (p-adj < 0.05 and > 1.5-fold) in *Pcgf1/3/5/6$^{fl/fl}$* ESCs following OHT treatment between different classes of genes (Non-NMI, Non-PcG, PcG) as described in Figure S1C.
- **(D)** Expression of pluripotency-associated genes in *Pcgf1/3/5/6$^{fl/fl}$* and *Ring1a$^{-/-}$; Ring1b$^{fl/fl}$* ESCs (UNT and OHT) as well as in ESCs before (-RA) and after 72 hr retinoic acid treatment (+RA) to induce differentiation. Read counts from cnRNA-seq (*Pcgf1/3/5/6$^{fl/fl}$* and *Ring1a$^{-/-}$; Ring1b$^{fl/fl}$*) or 4sU RNA-seq (RA-treated ESCs (Dimitrova et al., 2018)) were normalised to the average gene expression in the corresponding untreated cells. Error bars show SEM (n=3).
- **(E)** UpSet plot for significantly upregulated genes in *Pcgf1$^{fl/fl}$*, *Pcgf1/3/5$^{fl/fl}$*, *Pcgf1/3/5/2$^{fl/fl}$*, *Pcgf1/3/5/6$^{fl/fl}$*, and *Ring1a$^{-/-}$; Ring1b$^{fl/fl}$* ESCs following OHT treatment. The number of genes in each set is shown in the bar chart on the left. All non-empty intersections for each set were grouped together and sorted by the number of overlapping sets. Intersections of each set of genes with the set of genes upregulated in *Ring1a$^{-/-}$; Ring1b$^{fl/fl}$* ESCs are highlighted in red.
- **(F)** As in (E) for significantly downregulated genes (p-adj < 0.05 and > 1.5-fold).
- **(G)** A Venn diagram illustrating the classification of variant PRC1-regulated genes used for the heatmaps in Figure 7E and Figure S7H. PCGF1-sensitive genes were defined as genes significantly upregulated following OHT treatment in *Pcgf1$^{fl/fl}$* ESCs. PCGF1/3/5-sensitive genes were defined as the set of genes significantly upregulated following OHT treatment in *Pcgf1/3/5$^{fl/fl}$* ESCs, excluding the PCGF1-sensitive set of genes. Similarly, PCGF1/3/5/6-sensitive genes were defined as the set of genes significantly upregulated following OHT treatment in *Pcgf1/3/5/6$^{fl/fl}$* ESCs, excluding the PCGF1- and PCGF1/3/5-sensitive sets of genes.
- **(H)** Heatmaps of H2AK119ub1 cChIP-seq in *Pcgf1$^{fl/fl}$*, *Pcgf1/3/5$^{fl/fl}$*, and *Pcgf1/3/5/6$^{fl/fl}$* ESCs (UNT and OHT) at promoters of variant PRC1-regulated genes. Classification of genes into PCGF1-, PCGF1/3/5- and PCGF1/3/5/6- sensitive is as in (G).
