# Supplementary material for: Synergy between Variant PRC1 Complexes Defines Polycomb-Mediated Gene Repression
Source: Mol Cell. 2019 Jun 6;74(5):1020–1036.e8. doi: 10.1016/j.molcel.2019.03.024 (PMC6561741; doi:10.1016/j.molcel.2019.03.024)
Supplement: Document S2. Article plus Supplemental Information [file mmc5.pdf]

Molecular Cell

Article

Synergy between Variant PRC1 Complexes Defines Polycomb-Mediated Gene Repression

Graphical Abstract

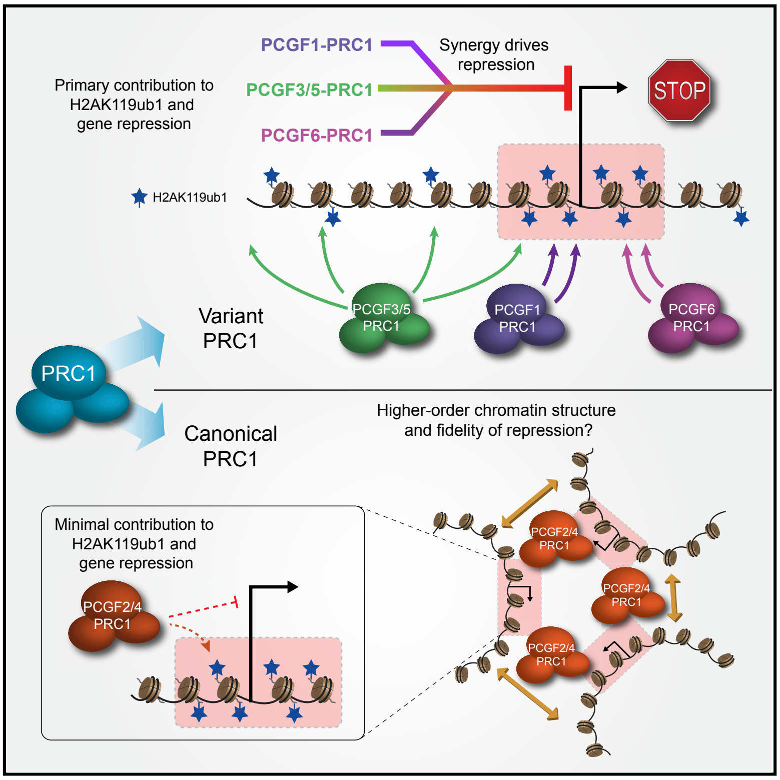

Authors

Nadezda A. Fursova, Neil P. Blackledge, Manabu Nakayama, ..., Hamish W. King, Haruhiko Koseki, Robert J. Klose

Correspondence

rob.klose@bioch.ox.ac.uk

In Brief

In this article, Fursova et al. uncover the central determinants of Polycomb-mediated gene repression in ESCs. They demonstrate that deposition of H2AK119ub1 and gene repression is driven by synergy between variant PRC1 complexes with little contribution from canonical PRC1 complexes, which mediate higher-order chromatin structures.

Highlights

- Canonical PRC1 complexes contribute little to H2AK119ub1 and gene repression
- Variant PRC1 complexes deposit H2AK119ub1 broadly throughout the genome
- Pervasive deposition of H2AK119ub1 by PCGF3/5-PRC1 is linked to X chromosome silencing
- Synergy between variant PRC1 complexes defines Polycomb-mediated gene repression

Fursova et al., 2019, Molecular Cell *74*, 1020–1036
June 6, 2019 © 2019 The Author(s). Published by Elsevier Inc.
https://doi.org/10.1016/j.molcel.2019.03.024

Synergy between Variant PRC1 Complexes Defines Polycomb-Mediated Gene Repression

Nadezda A. Fursova,[1,5] Neil P. Blackledge,[1,5] Manabu Nakayama,[2] Shinsuke Ito,[3] Yoko Koseki,[3] Anca M. Farcas,[1] Hamish W. King,[1] Haruhiko Koseki,[3,4] and Robert J. Klose[1,6,*]
[1]Department of Biochemistry, University of Oxford, South Parks Road, Oxford OX1 3QU, UK
[2]Laboratory of Medical Omics Research, Department of Frontier Research and Development, Kazusa DNA Research Institute, 2-6-7 Kazusa-Kamatari, Kisarazu, Chiba 292-0818, Japan
[3]Laboratory for Developmental Genetics, RIKEN Center for Integrative Medical Sciences, 1-7-29 Suehiro-cho, Tsurumi-ku, Yokohama, Kanagawa 230-0045, Japan
[4]AMED-CREST, Japanese Agency for Medical Research and Development, 1-7-22 Suehiro-cho, Tsurumi-ku, Yokohama 230-0045, Japan
[5]These authors contributed equally
[6]Lead Contact
*Correspondence: rob.klose@bioch.ox.ac.uk
https://doi.org/10.1016/j.molcel.2019.03.024

SUMMARY

The Polycomb system modifies chromatin and plays an essential role in repressing gene expression to control normal mammalian development. However, the components and mechanisms that define how Polycomb protein complexes achieve this remain enigmatic. Here, we use combinatorial genetic perturbation coupled with quantitative genomics to discover the central determinants of Polycomb-mediated gene repression in mouse embryonic stem cells. We demonstrate that canonical Polycomb repressive complex 1 (PRC1), which mediates higher-order chromatin structures, contributes little to gene repression. Instead, we uncover an unexpectedly high degree of synergy between variant PRC1 complexes, which is fundamental to gene repression. We further demonstrate that variant PRC1 complexes are responsible for distinct pools of H2A monoubiquitylation that are associated with repression of Polycomb target genes and silencing during X chromosome inactivation. Together, these discoveries reveal a new variant PRC1-dependent logic for Polycomb-mediated gene repression.

INTRODUCTION

In multicellular organisms, the specification and maintenance of highly defined gene expression patterns is required for tissue organization and normal development. Gene expression is primarily controlled by transcription factors that bind regulatory elements and define RNA polymerase II recruitment and activity at gene promoters. However, in addition to these DNA-encoded mechanisms, it has become clear that the chromatin template on which transcription occurs can profoundly regulate gene expression, particularly during development. This often relies on mobilization of nucleosomes by chromatin remodeling enzymes and post-translational modification of histones to create chromatin states that can potentiate or inhibit transcription (Atlasi and Stunnenberg, 2017; Lai and Pugh, 2017).

The Polycomb system is an essential chromatin-based regulator of gene expression. The first Polycomb phenotype was identified in *Drosophila* over 70 years ago (Lewis, 1978; Schuettengruber et al., 2017), and genetic screens (Gaytán de Ayala Alonso et al., 2007; Jürgens, 1985) have identified a multitude of Polycomb group (PcG) genes that can regulate gene expression and development. PcG proteins form large multi-component complexes with histone-modifying activities, suggesting that they function through chromatin-based and possibly epigenetic mechanisms. The best characterized of these complexes are PRC1 (Shao et al., 1999), which monoubiquitylates histone H2A at lysine 119 (to form H2AK119ub1; Wang et al., 2004a), and PRC2, which mono-, di-, and tri-methylates histone H3 at lysine 27 (to form H3K27me1, me2, and me3; Cao et al., 2002; Czermin et al., 2002; Kuzmichev et al., 2002; Müller et al., 2002). Polycomb systems function at gene-regulatory sites, where their activities inhibit the expression of associated genes (reviewed in Blackledge et al., 2015; Di Croce and Helin, 2013; Schuettengruber et al., 2017).

In mammals, it has been proposed that PcG proteins are initially targeted to promoter-associated gene regulatory elements, called CpG islands, through DNA binding factors and/or RNA (Blackledge et al., 2015; Farcas et al., 2012; He et al., 2013; Li et al., 2017; Perino et al., 2018; Schuettengruber et al., 2017). Following this initial recruitment, PRC1 and PRC2 occupancy is stabilized through the placement and subsequent recognition of Polycomb-specific histone modifications. This creates feedback loops that amplify the formation of transcriptionally repressive Polycomb chromatin domains containing PRC1, PRC2, H2AK119ub1, and H3K27me3 (Blackledge et al., 2014; Cooper et al., 2016; Margueron et al., 2009; Poux et al., 2001; Rose et al., 2016; Wang et al., 2004b). Despite a detailed description of the proteins that make up Polycomb repressive complexes (Conway et al., 2018; Gao et al., 2012; Hauri et al., 2016; Kloet et al., 2016; Smits et al., 2013; Tavares et al., 2012), and some understanding of their molecular interactions on chromatin (Blackledge et al., 2014; Wang et al., 2004b), the

 © 2019 The Author(s). Published by Elsevier Inc.
This is an open access article under the CC BY license (http://creativecommons.org/licenses/by/4.0/).

central components and mechanisms that define how Polycomb systems regulate gene expression in mammals remain unknown.

Uncovering the determinants of Polycomb-mediated gene repression has proven challenging due to the number and complexity of protein assemblies that comprise PRC1 and PRC2 (Hauri et al., 2016). This complexity is exemplified by PRC1, which contains one of two interchangeable E3 ubiquitin ligases (RING1A or RING1B) that dimerizes with a PCGF protein to support catalysis. In mammals, six PCGF proteins form an array of biochemically distinct multi-protein PRC1 complexes (Gao et al., 2012; Hauri et al., 2016). This multiplicity is thought to provide unique targeting modalities and regulatory capacity to PRC1. For example, PRC1 complexes that contain chromobox (CBX) proteins, often referred to as canonical PRC1 complexes, can bind to H3K27me3 to occupy chromatin modified by PRC2. Canonical PRC1 complexes have been proposed to compact chromatin and mediate higher-order chromatin structures, and it has been widely postulated that these activities are a central determinant of PRC1-mediated gene repression (Francis et al., 2004; Isono et al., 2013; Lau et al., 2017; Wang et al., 2004b). Conversely, PRC1 complexes that lack CBX proteins but contain RYBP or YAF2 in their place form variant PRC1 complexes (Gao et al., 2012; Morey et al., 2013; Tavares et al., 2012). Variant PRC1 complexes are the most active H2AK119 ubiquitin ligases *in vitro* and have also been proposed to contribute to gene repression in more specialized contexts (Blackledge et al., 2015; Gao et al., 2012; Rose et al., 2016; Schuettengruber et al., 2017). Extensive efforts have been placed on studying the function of individual PcG complexes *in vivo*, but this has failed to uncover the central determinants of Polycomb-mediated gene repression. Therefore, the logic by which PcG systems function in normal biology and how their perturbation leads to human pathogenesis, such as cancer, remains elusive.

Here, we have exploited systematic combinatorial genome editing and calibrated genomic approaches to discover the determinants of Polycomb-mediated gene repression in mouse embryonic stem cells (ESCs). We demonstrate that PRC1 is central to gene repression by the Polycomb system, though canonical PRC1 complexes contribute very little to this process. Instead, we discover that variant PRC1 complexes play a fundamental and synergistic role in shaping genomic H2AK119ub1, supporting communication between PRC1 and PRC2 to form Polycomb chromatin domains, and defining gene repression. Together, this reveals a variant PRC1-dependent logic for Polycomb activity and defines the molecular determinants required for Polycomb-mediated gene repression.

RESULTS

Canonical PRC1 Is Not Required for Gene Repression

In mammals, canonical PRC1 complexes assemble around either PCGF2 or PCGF4 and contain CBX proteins, which bind H3K27me3, and polyhomeotic (PHC) proteins that can polymerize (Figure 1A; Gao et al., 2012; Hauri et al., 2016; Isono et al., 2013; Kim et al., 2002). Biochemical studies *in vitro* have shown that canonical PRC1 complexes can bind, bridge, and compact nucleosomal arrays in a manner that does not require histone tails (Francis et al., 2004; Grau et al., 2011; Lavigne et al., 2004). Furthermore, in cells, canonical PRC1 function is linked to the formation of higher-order chromatin interactions, which rely on PHC proteins but appear to occur independently of H2AK119ub1 (Boettiger et al., 2016; Eskeland et al., 2010; Francis et al., 2004; Kundu et al., 2017; Lau et al., 2017; Lavigne et al., 2004; Wani et al., 2016). Therefore, it has been proposed that deposition of H3K27me3 by PRC2 recruits canonical PRC1 to Polycomb target genes in order to compact chromatin and drive Polycomb-dependent transcriptional repression.

In ESCs, canonical PRC1 complexes have previously been studied in constitutive RNAi or gene knockout systems (Kundu et al., 2017; Morey et al., 2015), in which primary gene expression defects are often masked by secondary changes in transcription and compensatory selection events. To overcome these limitations and define the primary contribution of canonical PRC1 to gene regulation, we developed a conditional ESC deletion system in which PCGF2 removal can be rapidly induced by tamoxifen (OHT) treatment (Figure 1B). Although PCGF4 is not expressed in ESCs (Gil et al., 2005), we also deleted the *Pcgf4* gene to mitigate possible compensation. Treatment of *Pcgf4*$^{-/-}$;*Pcgf2*$^{fl/fl}$ ESCs with OHT resulted in a complete loss of PCGF2 protein and major reductions in the levels of CBX7 and PHC1 (Figure 1C). In addition, RING1B protein levels were reduced (Figure 1C), suggesting that a failure to form functional canonical PRC1 complexes (Figure S1A) destabilizes RING1B. These observations are in agreement with a requirement for PCGF2 in canonical PRC1 complex formation and stability (Morey et al., 2015). We then carried out calibrated nuclear RNA sequencing (cnRNA-seq) and found very few genes with significant expression changes (Figures 1E and S1B). In contrast, removal of RING1A/B, the core components of both canonical and variant PRC1 complexes, resulted in derepression of several thousand genes (Figure 1E), most of which were classical Polycomb target genes bound by both PRC1 and PRC2 (Figures S1C and S1D). Therefore, canonical PRC1 complexes are not the central determinant of Polycomb-mediated gene repression in ESCs.

Canonical PRC1 Shapes RING1B Occupancy, but Not H2AK119ub1 or PRC2 Activity

The maintenance of Polycomb-mediated gene repression following removal of PCGF2/4 prompted us to examine whether Polycomb chromatin domains were also retained. Using calibrated chromatin immunoprecipitation sequencing (cChIP-seq) (Bonhoure et al., 2014; Hu et al., 2015; Orlando et al., 2014), we first confirmed that PCGF2 was absent from chromatin following OHT treatment (Figures 1F, 1G, S1E, and S1G). We then examined the binding of RING1B, a component shared among PRC1 complexes, and CBX7 and PHC1, which are canonical PRC1-specific subunits. This revealed that, following PCGF2/4 removal, there were major and widespread reductions in RING1B and CBX7 occupancy at classical Polycomb chromatin domains (Figures 1F–1H and S1E–S1I) in agreement with previous knockdown experiments (Morey et al., 2015). Furthermore, binding of PHC1, which is central to chromatin compaction by canonical PRC1, was lost (Figures 1F, 1G, S1E, and S1G). Removal of PCGF2/4 caused a major reduction in

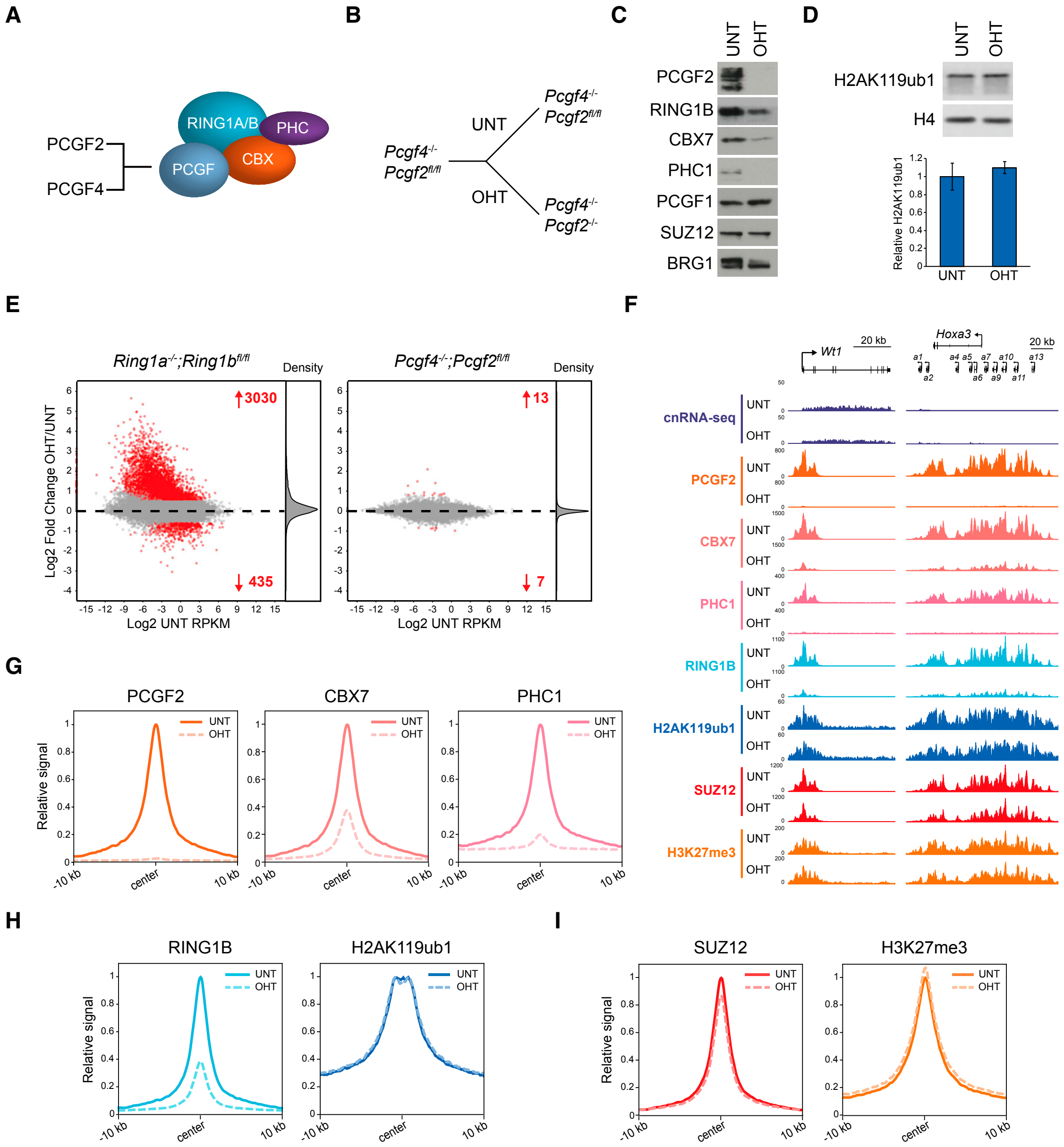

Figure 1. Canonical PRC1 Is Not Required for Polycomb-Mediated Gene Repression

(A) A schematic of PCGF2- or PCGF4-containing canonical PRC1 complexes.

(B) A schematic of the *Pcgf4*$^{-/-}$;*Pcgf2*$^{fl/fl}$ ESCs in which addition of OHT leads to removal of PCGF2 and loss of canonical PRC1.

(C) Western blots of PRC1 and PRC2 factors in untreated (UNT) and OHT-treated *Pcgf4*$^{-/-}$;*Pcgf2*$^{fl/fl}$ ESCs. BRG1 is shown as a loading control.

(D) Western blot for H2AK119ub1 in *Pcgf4*$^{-/-}$;*Pcgf2*$^{fl/fl}$ ESCs (UNT and OHT) together with quantification of H2AK119ub1 levels relative to histone H4. Error bars represent SEM (n = 3).

(E) MA plots of log2-fold changes in gene expression (cnRNA-seq) in *Ring1a*$^{-/-}$;*Ring1b*$^{fl/fl}$ (left) and *Pcgf4*$^{-/-}$;*Pcgf2*$^{fl/fl}$ (right) ESCs following OHT treatment. Significant gene expression changes (p-adj < 0.05 and >1.5-fold) are shown in red. Density of gene expression changes is shown on the right.

(F) Genomic snapshots of typical Polycomb target genes illustrating cnRNA-seq and cChIP-seq for canonical PRC1 (PCGF2, CBX7, and PHC1), PRC1 (RING1B and H2AK119ub1), and PRC2 (SUZ12 and H3K27me3) in the *Pcgf4*$^{-/-}$;*Pcgf2*$^{fl/fl}$ ESCs (UNT and OHT).

(G) Metaplots of canonical PRC1 (PCGF2, CBX7, and PHC1) cChIP-seq at classical Polycomb chromatin domains (n = 2,096) in *Pcgf4*$^{-/-}$;*Pcgf2*$^{fl/fl}$ ESCs (UNT and OHT).

(H) As in (G) for PRC1 (RING1B and H2AK119ub1).

(I) As in (G) for PRC2 (SUZ12 and H3K27me3).

See also Figure S1.

RING1B occupancy, but this did not translate into effects on H2AK119ub1 as quantified by bulk histone western blot analysis and cChIP-seq (Figures 1D, 1F, 1H, S1F, S1H, and S1I). This agrees with observations that canonical PRC1 complexes are weak E3 ubiquitin ligases *in vitro* (Gao et al., 2012; Rose et al., 2016; Taherbhoy et al., 2015) and *in vivo* (Blackledge et al., 2014). We and others have previously proposed that communication between PRC1 and PRC2 at Polycomb chromatin domains relies on the capacity of PRC2 to recognize H2AK119ub1 (Blackledge et al., 2014; Cooper et al., 2014, 2016; Kalb et al., 2014; Rose et al., 2016). In support of this idea, binding of SUZ12 (a core component of PRC2) and H3K27me3 remained largely unperturbed following loss of PCGF2/4 (Figures 1F, 1I, S1F, S1H, and S1I). Therefore, although PCGF2/4 play a central role in defining RING1B occupancy, they are not required for H2AK119ub1 deposition, PRC2 recruitment and activity, or repression of Polycomb target genes.

H2AK119ub1 Is Widespread but Enriched at PRC1-Bound Sites

Removal of PCGF2/4 led to major reductions in PRC1 binding but did not affect Polycomb-mediated gene repression, indicating that the remaining PRC1 or PRC2 activity must be sufficient to inhibit transcription. Previous work has shown that PRC2 removal does not lead to widespread derepression of Polycomb target genes in ESCs (Riising et al., 2014), suggesting that additional PRC1 activities must be responsible for gene repression. As H2AK119ub1 was unaffected in PCGF2/4-deficient cells, we postulated that this may be related to gene repression. Therefore, we set out to characterize in detail the genomic distribution of H2AK119ub1, as we reasoned that pinpointing which PRC1 complexes shape H2AK119ub1 could provide clues to the determinants of Polycomb-mediated gene repression.

To characterize H2AK119ub1 distribution in the genome, we carried out cChIP-seq in *Ring1a*$^{-/-}$*; Ring1b*$^{fl/fl}$ cells, where all PRC1 complexes and their activity can be removed (Figures 2A, 2B, and S2A). This revealed two important features of H2AK119ub1. First, as expected, RING1B-bound sites (herein referred to as PRC1-bound sites) were enriched for H2AK119ub1 (Figures 2C, 2D, and S2B) and H2AK119ub1 scaled with RING1B occupancy (Figure S2B). The sites most enriched for RING1B and H2AK119ub1 were associated with promoters of lowly transcribed genes that were also derepressed following RING1A/B removal, whereas sites with low to moderate levels of RING1B and H2AK119ub1 corresponded to promoters of genes that were more expressed and less susceptible to reactivation (Figure S2C). Second, we identified low-level yet ubiquitous H2AK119ub1 throughout the genome, indicating that PRC1 must transiently interact and place H2AK119ub1 at sites where it is not effectively captured by ChIP (Figures 2E, S2A, and S2D). This widespread H2AK119ub1 was evident when we visualized an entire chromosome or focused on genomic regions between sites with punctate high-level enrichment of RING1B and H2AK119ub1 (Figure 2E). In agreement with H2AK119ub1 being widespread, we estimated that 10% of H2A is monoubiquitylated (Figure 2B), consistent with earlier quantitation (Albright et al., 1979; Matsui et al., 1979). Together, our new analyses demonstrate that there are two distinct pools of H2AK119ub1: a highly enriched fraction that overlaps with stable RING1B binding and a low-level ubiquitous fraction that blankets the genome.

PCGF1-PRC1 Shapes Polycomb Chromatin Domains and Contributes to Gene Repression

Having identified two distinct pools of H2AK119ub1 in the genome, we wanted to understand how these are deposited and related to gene repression. Given that canonical PRC1 complexes made little or no contribution to H2AK119ub1 and gene repression, we engineered a series of OHT-inducible conditional alleles for the *Pcgf* genes that exclusively form variant PRC1 complexes (*Pcgf1*, *Pcgf3*, *Pcgf5*, and *Pcgf6*; Gao et al., 2012; Hauri et al., 2016; Figures 3A and 3B). We then carried out cChIP-seq for H2AK119ub1 as a simple screen to identify regions of the genome where individual PCGF-PRC1 complexes function. Removal of PCGF1 caused a major and specific reduction of punctate H2AK119ub1 at PRC1-bound sites, in agreement with previous findings that the PCGF1-PRC1 complex is targeted to Polycomb-occupied CpG islands (Figures 3C, 3D, and S3A–S3C; Farcas et al., 2012; He et al., 2013). However, similarly to removal of PCGF2, loss of either PCGF3 or PCGF5 had virtually no effect on H2AK119ub1 (Figures 3C, 3D, and S3A–S3C). Finally, PCGF6 removal caused minor reductions in H2AK119ub1 (Figures 3C, 3D, and S3A–S3C), with major losses only at a small subset of germ-cell-specific genes, which, as we have previously shown, require PCGF6 for H2AK119ub1 and repression (Figures S3D and S3E; Endoh et al., 2017).

Given that loss of PCGF1 had the largest effect on H2AK119ub1 at PRC1-bound sites, we next asked whether PCGF1-PRC1 also contributed to PRC2 activity and gene repression at these regions. In agreement with our previous observations that H2AK119ub1 shapes PRC2 activity at PRC1 target sites, removal of PCGF1-PRC1 led to a specific and substantial reduction in H3K27me3 (Figures 3E, 3F, S3F, and S3G; Blackledge et al., 2014; Rose et al., 2016). In addition, loss of PCGF1-PRC1 resulted in reactivation of hundreds of genes, most of which were Polycomb targets (Figures 3G and S3H), indicating that PCGF1-PRC1 plays an important role in Polycomb-mediated gene repression. However, PCGF1-dependent gene expression changes were considerably less dramatic than following removal of RING1A/B, both in number of derepressed genes and magnitude of derepression (compare Figures 3G, 1E, and S3I). Together, these observations reveal that the PCGF1-PRC1 complex is essential for shaping normal H2AK119ub1 and H3K27me3 at PRC1 target sites but is not sufficient to fully explain Polycomb-dependent gene repression.

Pervasive PCGF3/5-Dependent H2AK119ub1 Is Not Required for Polycomb Target Gene Repression but Is Linked to Xist-Mediated Chromosome Silencing

Systematic removal of individual PCGF proteins did not recapitulate the effects of removing RING1A/B, suggesting that individual PRC1 complexes must functionally cooperate to shape genomic H2AK119ub1 and repress transcription. Initially, we reasoned that the most closely related variant PRC1 complexes would likely underpin any potential cooperation. Therefore, using *Pcgf3/5*$^{fl/fl}$ ESCs (Figure 4A), we focused on PCGF3 and PCGF5,

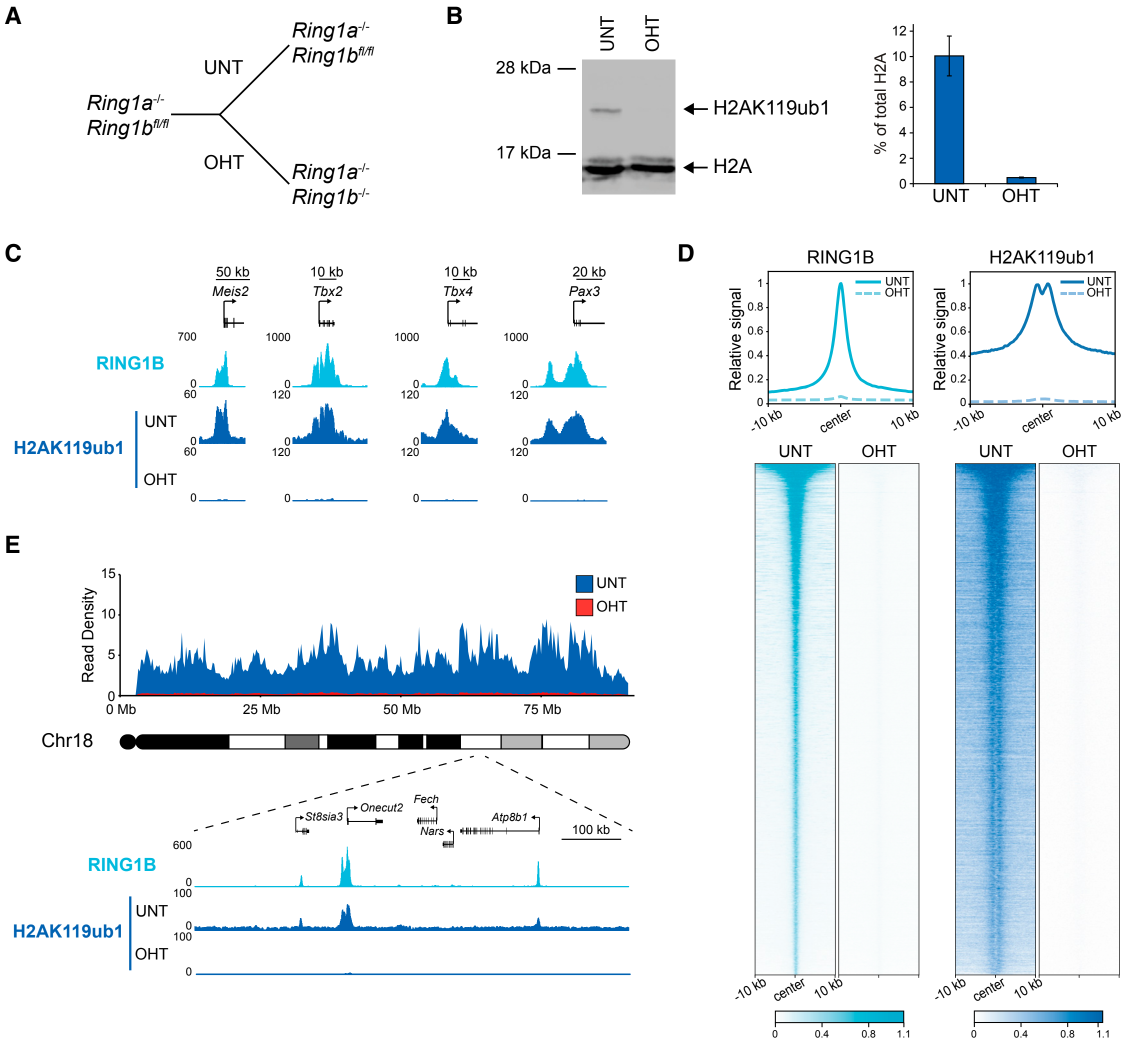

Figure 2. H2AK119ub1 Is Widespread but Enriched at Polycomb Chromatin Domains

(A) A schematic of the *Ring1a*$^{-/-}$;*Ring1b*$^{fl/fl}$ ESCs in which addition of OHT leads to removal of RING1B and loss of all PRC1.

(B) Western blot of H2AK119ub1 in *Ring1a*$^{-/-}$;*Ring1b*$^{fl/fl}$ ESCs before (UNT) and after OHT treatment, using an antibody against total histone H2A, together with quantification of H2AK119ub1 relative to histone H2A. Error bars represent SEM (n = 3).

(C) Genomic snapshots illustrating RING1B and H2AK119ub1 cChIP-seq in the *Ring1a*$^{-/-}$; *Ring1b*$^{fl/fl}$ ESCs (UNT and OHT).

(D) Metaplots and heatmaps of RING1B and H2AK119ub1 cChIP-seq at PRC1-bound sites (n = 8,833) in *Ring1a*$^{-/-}$;*Ring1b*$^{fl/fl}$ ESCs (UNT and OHT).

(E) A chromosome density plot showing H2AK119ub1 cChIP-seq across chromosome 18 in *Ring1a*$^{-/-}$;*Ring1b*$^{fl/fl}$ ESCs (UNT and OHT), together with an expanded genomic snapshot also showing RING1B cChIP-seq.

See also Figure S2.

which share high sequence similarity and form nearly identical protein complexes (Gao et al., 2012). Remarkably, despite H2AK119ub1 being unaffected following removal of PCGF3 or PCGF5 individually, loss of PCGF3/5 in combination resulted in a major reduction in H2AK119ub1 by western blot (Figure 4B) and a profound and uniform depletion of H2AK119ub1 throughout the genome by cChIP-seq (Figures 4C–4E, S4A, and S4B). This demonstrates an unexpected role of PCGF3/5-PRC1 in depositing low-level H2AK119ub1 genome-wide. Nevertheless, despite these global reductions in H2AK119ub1, the punctate enrichment of H2AK119ub1 at PRC1-bound sites was retained following PCGF3/5 removal (Figures 4C, 4E, S4A, and S4C), as was H3K27me3 enrichment (Figures S4D–S4F). More importantly, in the absence of PCGF3/5, very few Polycomb target genes were derepressed (Figures 4F, S4G, and S4H). Furthermore, we found no evidence for PCGF3/5-dependent activation of gene expression, as has been previously reported (Gao et al., 2014; Yao et al., 2018; Zhao et al., 2017a).

Although PCGF3/5-PRC1 complexes were not required for Polycomb target gene repression, they have recently been shown to support Xist-mediated chromosome silencing and H2AK119ub1 accumulation on the silenced X chromosome as

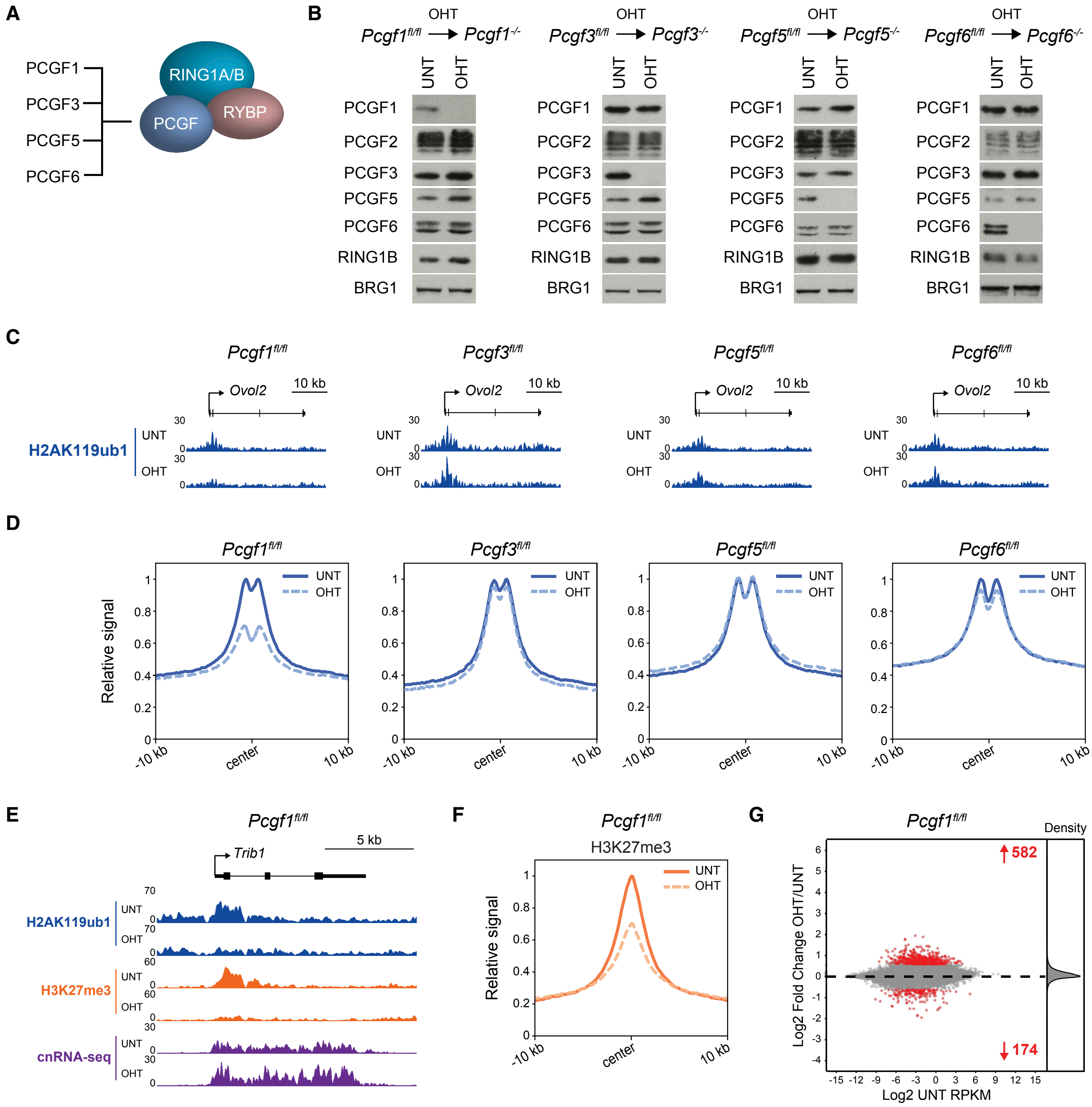

Figure 3. PCGF1-PRC1 Shapes Polycomb Chromatin Domains and Contributes to Gene Repression

(A) A schematic of the PCGF1, PCGF3, PCGF5, and PCGF6 variant PRC1 complexes.

(B) Western blots of PCGF proteins and RING1B in *Pcgf1*$^{fl/fl}$, *Pcgf3*$^{fl/fl}$, *Pcgf5*$^{fl/fl}$, and *Pcgf6*$^{fl/fl}$ ESCs before (UNT) and after OHT treatment. BRG1 is shown as a loading control.

(C) Genomic snapshots of typical Polycomb target genes showing H2AK119ub1 cChIP-seq in *Pcgf1*$^{fl/fl}$, *Pcgf3*$^{fl/fl}$, *Pcgf5*$^{fl/fl}$, and *Pcgf6*$^{fl/fl}$ ESCs (UNT and OHT).

(D) Metaplots of H2AK119ub1 cChIP-seq at PRC1-bound sites in *Pcgf1*$^{fl/fl}$, *Pcgf3*$^{fl/fl}$, *Pcgf5*$^{fl/fl}$, and *Pcgf6*$^{fl/fl}$ ESCs (UNT and OHT).

(E) A genomic snapshot of a typical Polycomb target gene, showing H2AK119ub1 and H3K27me3 cChIP-seq and cnRNA-seq in *Pcgf1*$^{fl/fl}$ ESCs (UNT and OHT).

(F) A metaplot of H3K27me3 cChIP-seq at PRC1-bound sites in *Pcgf1*$^{fl/fl}$ ESCs (UNT and OHT).

(G) An MA plot of log2-fold changes in gene expression (cnRNA-seq) in *Pcgf1*$^{fl/fl}$ ESCs following OHT treatment. Significant gene expression changes (p-adj < 0.05 and >1.5-fold) are shown in red.

See also Figure S3.

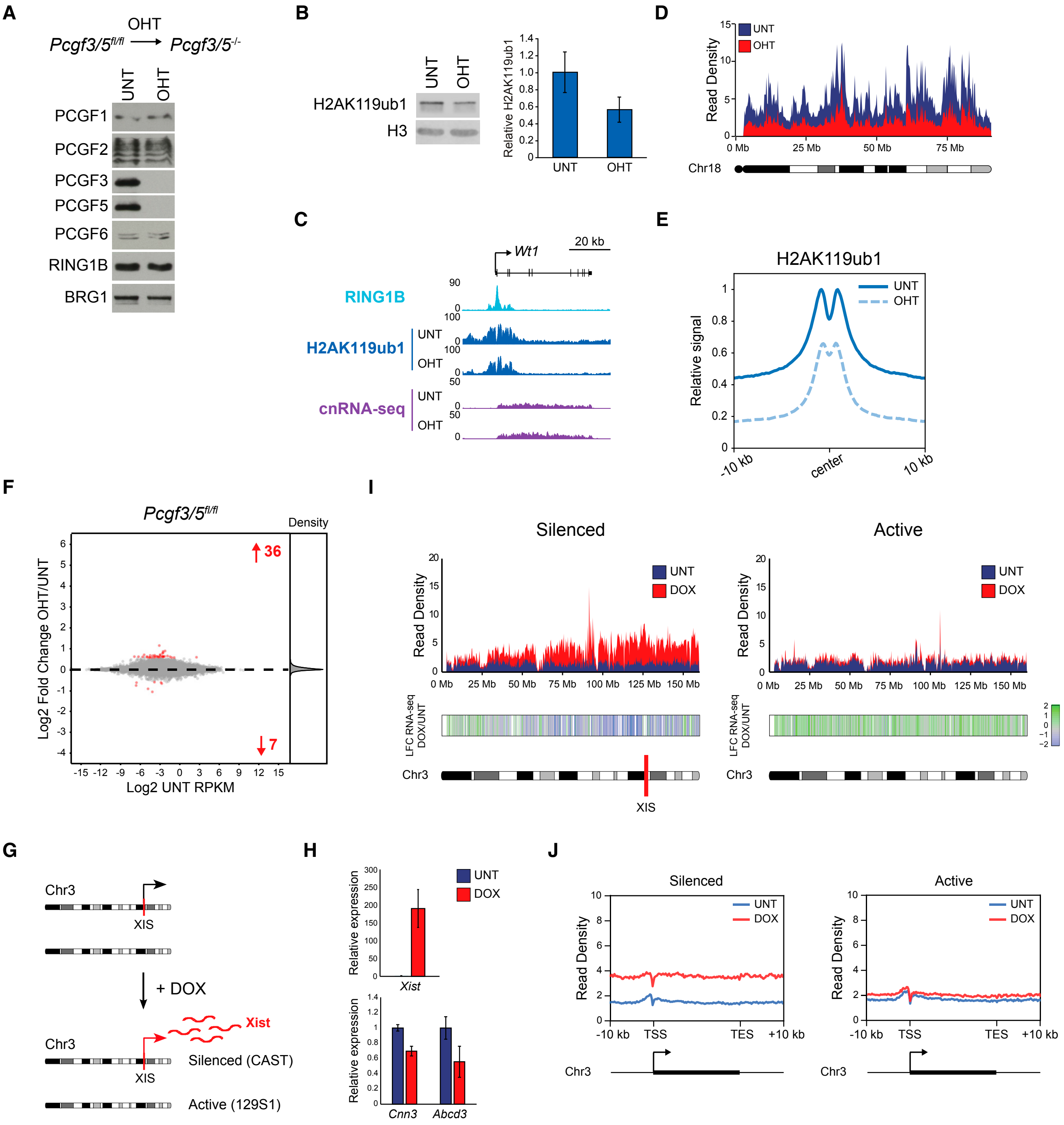

Figure 4. Pervasive PCGF3/5-Dependent H2AK119ub1 Is Dispensable for Classical Polycomb Target Gene Repression but Is Associated with Xist-Mediated Chromosome Silencing

(A) Western blots of PCGF proteins and RING1B in *Pcgf3/5$^{fl/fl}$* ESCs before (UNT) and after OHT treatment. BRG1 is shown as a loading control.

(B) Western blot for H2AK119ub1 in *Pcgf3/5$^{fl/fl}$* ESCs (UNT and OHT) together with quantification of H2AK119ub1 levels relative to histone H3. Error bars represent SEM (n = 3).

(C) A genomic snapshot of a typical Polycomb target gene illustrating H2AK119ub1 and RING1B cChIP-seq and cnRNA-seq in *Pcgf3/5$^{fl/fl}$* ESCs (UNT and OHT).

(D) A chromosome density plot showing H2AK119ub1 cChIP-seq across chromosome 18 in *Pcgf3/5$^{fl/fl}$* ESCs (UNT and OHT).

(E) A metaplot of H2AK119ub1 cChIP-seq at PRC1-bound sites in *Pcgf3/5$^{fl/fl}$* ESCs (UNT and OHT).

(F) An MA plot of log2-fold changes in gene expression (cnRNA-seq) in *Pcgf3/5$^{fl/fl}$* ESCs following OHT treatment. Significant gene expression changes (p-adj < 0.05 and >1.5-fold) are shown in red.

(legend continued on next page)

examined by immunofluorescence (Almeida et al., 2017; Pintacuda et al., 2017). Therefore, we wondered whether pervasive PCGF3/5-dependent H2AK119ub1 might play a specialized and context-dependent role in chromosome-wide gene repression. To examine this possibility, we performed H2AK119ub1 cChIP-seq in an interspecies hybrid ESC line in which *Xist* expression and gene silencing can be induced on one of two autosomal alleles (Figures 4G and 4H; Pintacuda et al., 2017). Strikingly, *Xist* induction resulted in broad accumulation of H2AK119ub1 across the *Xist*-expressing chromosome with no preference for gene promoters or pre-existing PRC1-bound sites (Figures 4I, 4J, and S4I–S4L). This was reminiscent of the genome-wide H2AK119ub1 deposition by PCGF3/5-PRC1, albeit elevated in magnitude. Indeed, recent studies have demonstrated that broad H2AK119ub1 acquisition during Xist-mediated chromosome silencing relies on PCGF3/5 (Nesterova et al., 2018) and the region of the *Xist* RNA that recruits PCGF3/5 (Bousard et al., 2018). Importantly, spreading of H2AK119ub1 across the silenced chromosome scaled with the distance from the *Xist* integration site and correlated well with gene repression (Figures 4I and S4M). Therefore, in contrast to classical Polycomb-dependent gene repression, which is associated with punctate Polycomb chromatin domains at target gene promoters, PCGF3/5-PRC1 and ubiquitous H2AK119ub1 appear to play a context-dependent role in repressing gene expression during Xist-mediated chromosome silencing.

Loss of PCGF1/3/5 Erodes Polycomb Chromatin Domains but Does Not Fully Compromise Gene Repression

Removal of PCGF1 led to a partial derepression of Polycomb target genes and removal of PCGF3/5 had virtually no effect on gene expression. However, as these complexes independently contributed to H2AK119ub1 at PRC1-bound sites, we reasoned that PCGF1/3/5-PRC1 complexes may cooperate to support gene repression. We therefore developed a triple conditional knockout system enabling simultaneous removal of PCGF1/3/5 (Figure 5A). Western blot analysis revealed a dramatic loss of H2AK119ub1 following PCGF1/3/5 removal (Figure 5B), and cChIP-seq showed dramatic H2AK119ub1 depletion at PRC1-bound sites (Figures 5C, 5D, and S5A) and throughout the genome (Figure S5D). We also observed an extensive erosion of other Polycomb chromatin domain features, including binding of RING1B and SUZ12, and H3K27me3 (Figures 5C, 5D, and S5A). More importantly, in contrast to the modest effect on repression of Polycomb target genes in PCGF1 or PCGF3/5-deficient cells, removal of PCGF1/3/5 in combination led to reactivation of a large number of genes (Figure 5E), most of which were Polycomb targets (Figure S5F). However, these widespread gene expression changes still failed to recapitulate the breadth and magnitude of Polycomb target gene derepression observed following RING1A/B removal (Figure 5F).

Canonical PRC1 Is Unable to Compensate for Compromised Variant PRC1 Complex Activity

We reasoned that, in the absence of PCGF1/3/5, the remaining repressive capacity of PRC1 must lie within the PCGF2- and/or PCGF6-containing complexes. Initially, we hypothesized that, when the repressive activity of variant PRC1 is compromised, a contribution of canonical PCGF2-PRC1 complexes, which could bind the remaining H3K27me3, may be unmasked. Therefore, we generated a PCGF1/3/5/2 quadruple conditional knockout ESC line (Figure 5G). However, following removal of PCGF2 in addition to PCGF1/3/5, we observed no further reduction in H2AK119ub1 at the majority of PRC1-bound sites (Figures 5I, 5J, S5A, and S5B) or throughout the genome (Figures 5H and S5E), despite RING1B binding being further reduced (Figures 5I, 5J, S5A, and S5B). Similarly, reductions in SUZ12 binding and H3K27me3 following removal of PCGF1/3/5/2 were highly comparable to the changes observed in PCGF1/3/5-deficient cells (Figures 5I, 5J, S5A, and S5B). This again suggests that, although PCGF2 shapes RING1B occupancy, this has minimal effects on H2AK119ub1 and PRC1-dependent stimulation of PRC2 binding and activity. Finally, following PCGF1/3/5/2 removal, the extent of Polycomb target gene reactivation was only marginally increased when compared to PCGF1/3/5-deficient cells (Figures 5F, 5K, S5C, and S5F) and still did not recapitulate the gene expression changes following RING1A/B removal (Figure 5F). Importantly, PCGF4 did not compensate for PCGF2 loss in these experiments (Figure S5G). This further supports our conclusion that canonical PRC1 complexes are unable to drive repression of Polycomb target genes, even when variant PRC1 function is substantially perturbed.

Interestingly, 137 PRC1-repressed genes did exhibit an additional increase in expression in PCGF1/3/5/2-deficient compared to PCGF1/3/5-deficient cells (Figures S5H–S5K). These genes were associated with very large Polycomb chromatin domains and extremely high RING1B occupancy compared to typical PRC1-repressed genes (Figure S5J). Strikingly, promoters of these genes also displayed a further reduction in H2AK119ub1, SUZ12 occupancy, and H3K27me3 when PCGF2 was removed in combination with PCGF1/3/5 (Figures S5H and S5K). This indicates that PCGF2-PRC1 can contribute to H2AK119ub1, PRC1-PRC2 communication, and repression at a specialized group of Polycomb target genes, although this contribution is modest and only evident when variant PRC1

(G) A schematic of the *Mus domesticus* (129S1) × *Mus castaneus* F1 ESCs, in which a doxycycline (DOX)-inducible *Xist* transgene is integrated into one allele of chromosome 3. DOX treatment causes Xist-mediated silencing of the allele harboring the *Xist* integration site (XIS) (silenced allele, from *Mus castaneus*), but not the other allele (active, from *Mus domesticus*).

(H) qRT-PCR gene expression analysis for *Xist* transgene and two neighboring genes before (UNT) and after DOX treatment. Expression is normalized to *b-actin* and shown relative to the average expression in UNT cells. Error bars show SEM (n = 3).

(I) Chromosome density plots of H2AK119ub1 cChIP-seq across two alleles of chromosome 3 in the system described in (G). Below is a heatmap of corresponding log2-fold changes in gene expression upon addition of DOX. Bins containing no expressed genes are shown in white.

(J) Allele-specific metaplots of H2AK119ub1 cChIP-seq for the genes on chromosome 3 in the system described in (G).

See also Figure S4.

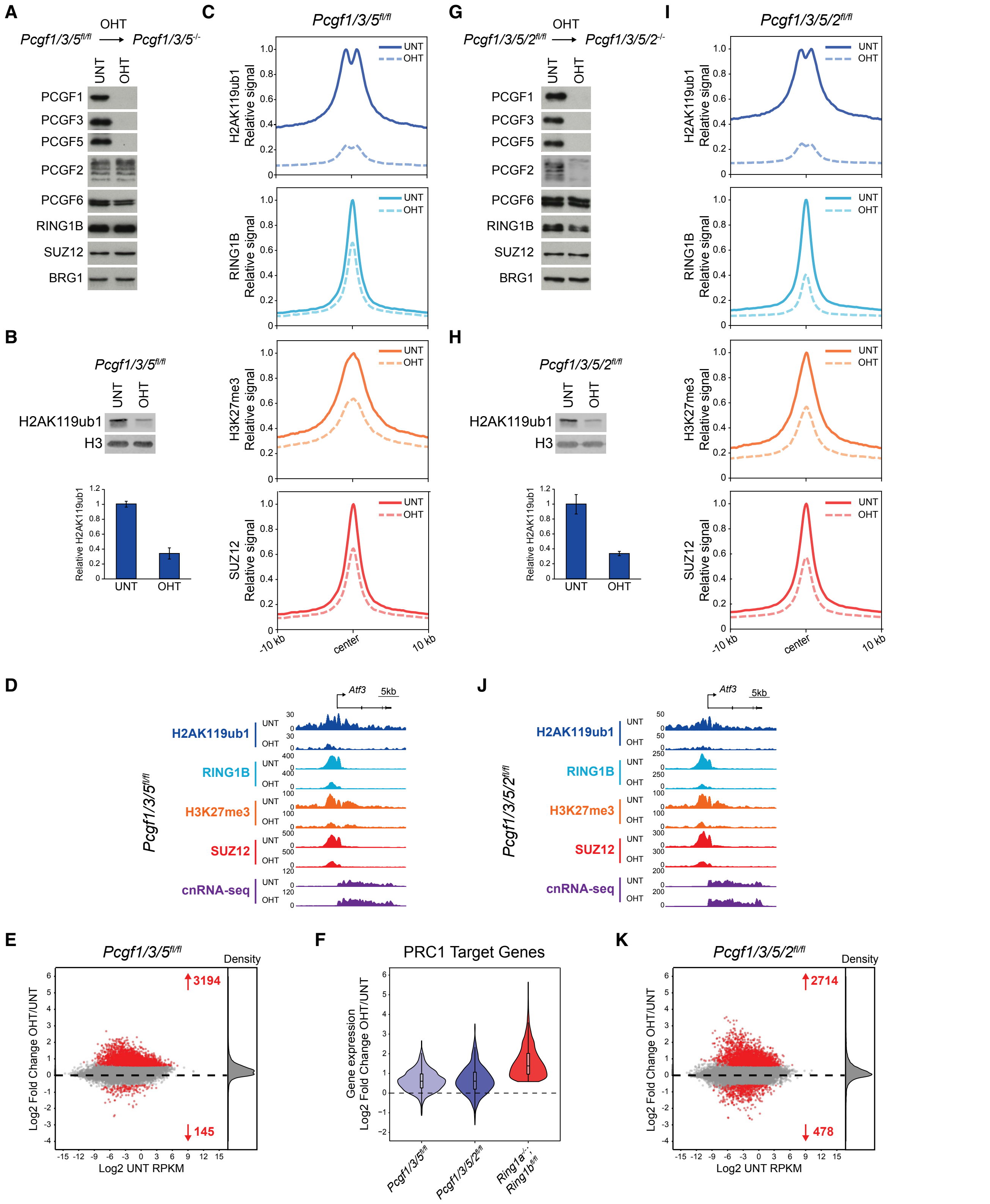

(legend on next page)

complexes are perturbed. At these sites, we speculate that extremely high occupancy enables PCGF2-PRC1 to partially support Polycomb chromatin domain formation and gene repression, despite the inherently weak E3 ubiquitin ligase activity of this complex (Rose et al., 2016) or that more active RYBP-containing PCGF2-PRC1 complexes (Gao et al., 2012; Rose et al., 2016) may contribute at these genes.

Variant PRC1 Complexes Synergize to Define Polycomb-Mediated Gene Repression

PCGF2-containing complexes were unable to account for the remaining repressive activity of PRC1 in PCGF1/3/5-deficient cells. This was surprising considering that PCGF6, the only remaining PCGF protein expressed in ESCs, has been shown to play a unique role in repressing germline-specific genes (Endoh et al., 2017; Suzuki et al., 2016; Zdzieblo et al., 2014; Zhao et al., 2017b). Nevertheless, to address the intriguing possibility that PCGF6 may also act more broadly in Polycomb-mediated gene repression, we generated a PCGF1/3/5/6 quadruple conditional knockout ESC line (Figure 6A). Strikingly, removal of PCGF6 in addition to PCGF1/3/5 had a dramatic effect on H2AK119ub1 (Figures 6B–6E, S6A, and S6B), which exceeded the combined contributions of the individual complexes (Figure 3). Importantly, we observed an almost complete loss of both the residual genome-wide H2AK119ub1 (Figures 6C and S6B) and punctate H2AK119ub1 at PRC1-bound sites (Figures 6D, 6E, S6A, and S6B). RING1B and SUZ12 occupancy and H3K27me3 were further compromised, indicating additional erosion of Polycomb chromatin domains (Figures 6E and S6A).

We did observe 354 PRC1 target gene promoters at which RING1B occupancy was retained (Figures S6C and S6D). At these sites, reductions in H2AK119ub1 were less pronounced and SUZ12 and H3K27me3 were largely unaffected (Figures S6C and S6D). Interestingly, like the sites at which PCGF2-PRC1 contributed to repressive Polycomb chromatin domains in the absence of PCGF1/3/5, these were large Polycomb chromatin domains with high levels of RING1B, which were associated with developmental genes (Figures S6D–S6G and S5J). This suggests that, at a small group of sites, both PCGF1/3/5/6- and PCGF2-containing complexes can shape Polycomb chromatin domains, which makes them more robust to transcriptional perturbation. However, this contrasts the vast majority of Polycomb-occupied sites at which Polycomb chromatin domains are greatly eroded following PCGF1/3/5/6 removal.

Next, we performed cnRNA-seq in the PCGF1/3/5/6 conditional knockout cells, which led to several fundamentally important observations. First, away from classical Polycomb target genes, we observed a widespread but modest increase in gene expression following removal of PCGF1/3/5/6 (Figures 7A, S7B, and S7C). Intriguingly, this effect was not seen in RING1A/B-deficient cells (Figures 1E and S1C), suggesting that, in some instances, variant PRC1-specific PCGF proteins can repress gene expression independently of RING1A/B or that variant PRC1 complexes may counteract a PCGF2-PRC1-dependent activity that supports gene expression. Indeed, there is accumulating evidence that PRC1 may potentiate as well as repress gene expression (Cohen et al., 2018; Creppe et al., 2014; Frangini et al., 2013; Gao et al., 2014; Morey et al., 2015). Second, and most importantly, removal of PCGF1/3/5/6 resulted in PRC1 target gene reactivation that largely recapitulated that observed in RING1A/B-deficient cells (Figures 7A, 1E, and S7A) both in the number of reactivated genes and the magnitude of reactivation (Figures 7B and 7C), revealing that variant PRC1 complexes define Polycomb-mediated gene repression. Importantly, both PCGF1/3/5/6- and RING1A/B-deficient cells retained expression of pluripotency factors (Figure S7D), suggesting that these gene expression changes were specific to loss of PRC1 activity and not the result of differentiation.

Comparing PRC1 target gene derepression across the complement of our PRC1 perturbation cell lines highlighted the fact that repression relies on synergy between variant PRC1 complexes (Figures 7B, 7C, S7E, and S7F): loss of PCGF1 caused a moderate reactivation of PRC1 target gene expression, whereas loss of PCGF2 or PCGF3/5 caused little if any derepression. When PCGF1/3/5 were removed together, there was a synergistic increase in gene expression when compared to PCGF1 and PCGF3/5 loss, which was not further potentiated by removal of PCGF2. However, gene reactivation analogous to that in RING1A/B-deficient cells was only achieved when PCGF6 was removed in combination with PCGF1/3/5. Moreover, when we examined the genomic distribution of individual PCGF proteins (and members of their protein complexes), we found that

Figure 5. Loss of PCGF1/3/5 Dramatically Erodes Polycomb Chromatin Domains but Does Not Fully Compromise Gene Repression, and This Is Not Further Potentiated by Removal of Canonical PRC1

(A) Western blots for PCGF proteins, RING1B, and SUZ12 in *Pcgf1/3/5*$^{fl/fl}$ ESCs before (UNT) and after OHT treatment. BRG1 is shown as a loading control.

(B) Western blot for H2AK119ub1 in *Pcgf1/3/5*$^{fl/fl}$ ESCs (UNT and OHT) together with quantification of H2AK119ub1 levels relative to histone H3. Error bars represent SEM (n = 3).

(C) Metaplots of PRC1 (H2AK119ub1 and RING1B) and PRC2 (H3K27me3 and SUZ12) cChIP-seq at PRC1-bound sites in *Pcgf1/3/5*$^{fl/fl}$ ESCs (UNT and OHT).

(D) A genomic snapshot of a typical Polycomb target gene, showing cnRNA-seq and cChIP-seq for PRC1 (H2AK119ub1 and RING1B) and PRC2 (H3K27me3 and SUZ12) in *Pcgf1/3/5*$^{fl/fl}$ ESCs (UNT and OHT).

(E) An MA plot of log2-fold changes in gene expression (cnRNA-seq) in *Pcgf1/3/5*$^{fl/fl}$ ESCs following OHT treatment. Significant gene expression changes (p-adj < 0.05 and >1.5-fold) are shown in red.

(F) A violin plot comparing log2-fold changes of PRC1 target gene expression in *Pcgf1/3/5*$^{fl/fl}$, *Pcgf1/3/5/2*$^{fl/fl}$, and *Ring1a*$^{-/-}$;*Ring1b*$^{fl/fl}$ ESCs following OHT treatment.

(G) As in (A) for *Pcgf1/3/5/2*$^{fl/fl}$ ESCs.

(H) As in (B) for *Pcgf1/3/5/2*$^{fl/fl}$ ESCs.

(I) As in (C) for *Pcgf1/3/5/2*$^{fl/fl}$ ESCs.

(J) As in (D) for *Pcgf1/3/5/2*$^{fl/fl}$ ESCs.

(K) As in (E) for *Pcgf1/3/5/2*$^{fl/fl}$ ESCs.

See also Figure S5.

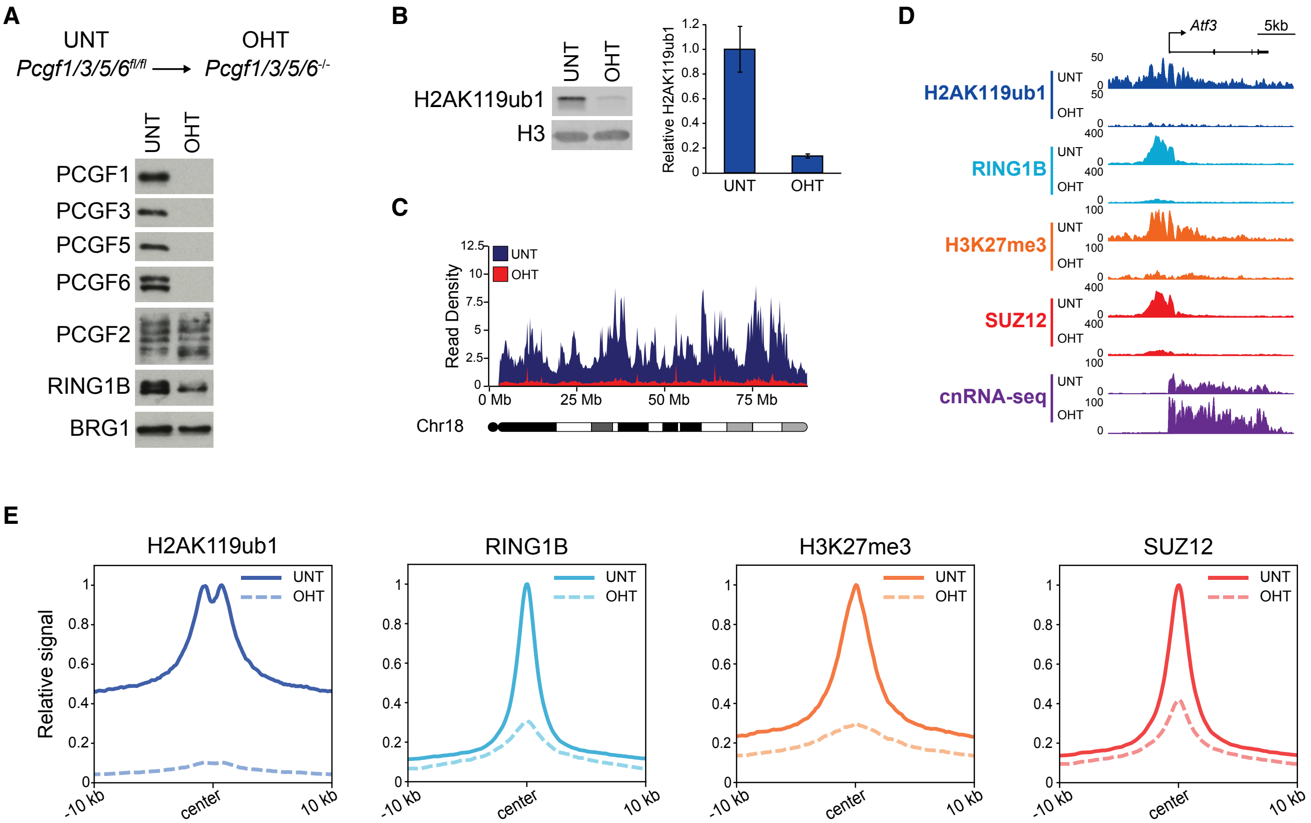

Figure 6. Variant PRC1 Complexes Define Polycomb Chromatin Domain Formation

(A) Western blots for PCGF proteins and RING1B in *Pcgf1/3/5/6$^{fl/fl}$* ESCs before (UNT) and after OHT treatment. BRG1 is shown as a loading control.

(B) Western blot for H2AK119ub1 in *Pcgf1/3/5/6$^{fl/fl}$* ESCs (UNT and OHT) together with quantification of H2AK119ub1 levels relative to histone H3. Error bars represent SEM (n = 3).

(C) A chromosome density plot showing H2AK119ub1 cChIP-seq across chromosome 18 in *Pcgf1/3/5/6$^{fl/fl}$* ESCs (UNT and OHT).

(D) A genomic snapshot of a typical Polycomb target gene, showing cnRNA-seq and cChIP-seq for PRC1 (H2AK119ub1 and RING1B) and PRC2 (H3K27me3 and SUZ12) in *Pcgf1/3/5/6$^{fl/fl}$* ESCs (UNT and OHT).

(E) Metaplots of PRC1 (H2AK119ub1 and RING1B) and PRC2 (H3K27me3 and SUZ12) cChIP-seq at PRC1-bound sites in *Pcgf1/3/5/6$^{fl/fl}$* ESCs (UNT and OHT). See also Figure S6.

variant PRC1 complexes broadly engage with promoters of the genes that they regulate (Figures 7D, 7E, and S7G) and contribute to H2AK119ub1 at these sites (Figure S7H). This was especially evident for PCGF1 (KDM2B) and PCGF6 (MGA and L3MBTL2), which showed promoter-specific enrichment at variant PRC1-regulated genes, whereas for PCGF5, this enrichment was less pronounced, in agreement with its role in pervasive H2AK119ub1 deposition. Despite the inherent limitations of comparing ChIP-seq signals derived from different antibodies, these PCGF occupancy profiles are consistent with our combinatorial genetic analysis and support our central conclusion that there is an unexpected and remarkably high level of synergy among variant PRC1 complexes that drives Polycomb chromatin domain formation and gene repression.

DISCUSSION

Using genome engineering and systematic genetic ablation coupled with calibrated genomics, here, we uncover the determinants of Polycomb-mediated gene repression in ESCs. We reveal that canonical PRC1 complexes contribute little to Polycomb target gene repression and H2AK119ub1. Instead, we discover that functionally distinct pools of genomic H2AK119ub1 correspond to defined modes of variant PRC1-dependent gene repression. Importantly, we reveal that synergy between variant PRC1 complexes is central to H2AK119ub1 deposition and Polycomb-mediated gene repression.

The nature of the relationship between PRC1, H2AK119ub1, and gene repression in mammals has remained a matter of active debate (Schuettengruber et al., 2017). This is in large part because the location of H2AK119ub1 in the genome has been poorly defined. Here, we discover that PCGF3/5-PRC1 deposit low-level H2AK119ub1 throughout the genome, consistent with earlier work in *Drosophila*, which indicated that H2AK119ub1 exists outside of classical Polycomb responsive elements (Kahn et al., 2016; Lee et al., 2015). However, loss of PCGF3/5-PRC1 does not lead to Polycomb target gene reactivation, suggesting the blanket of H2AK119ub1 may instead

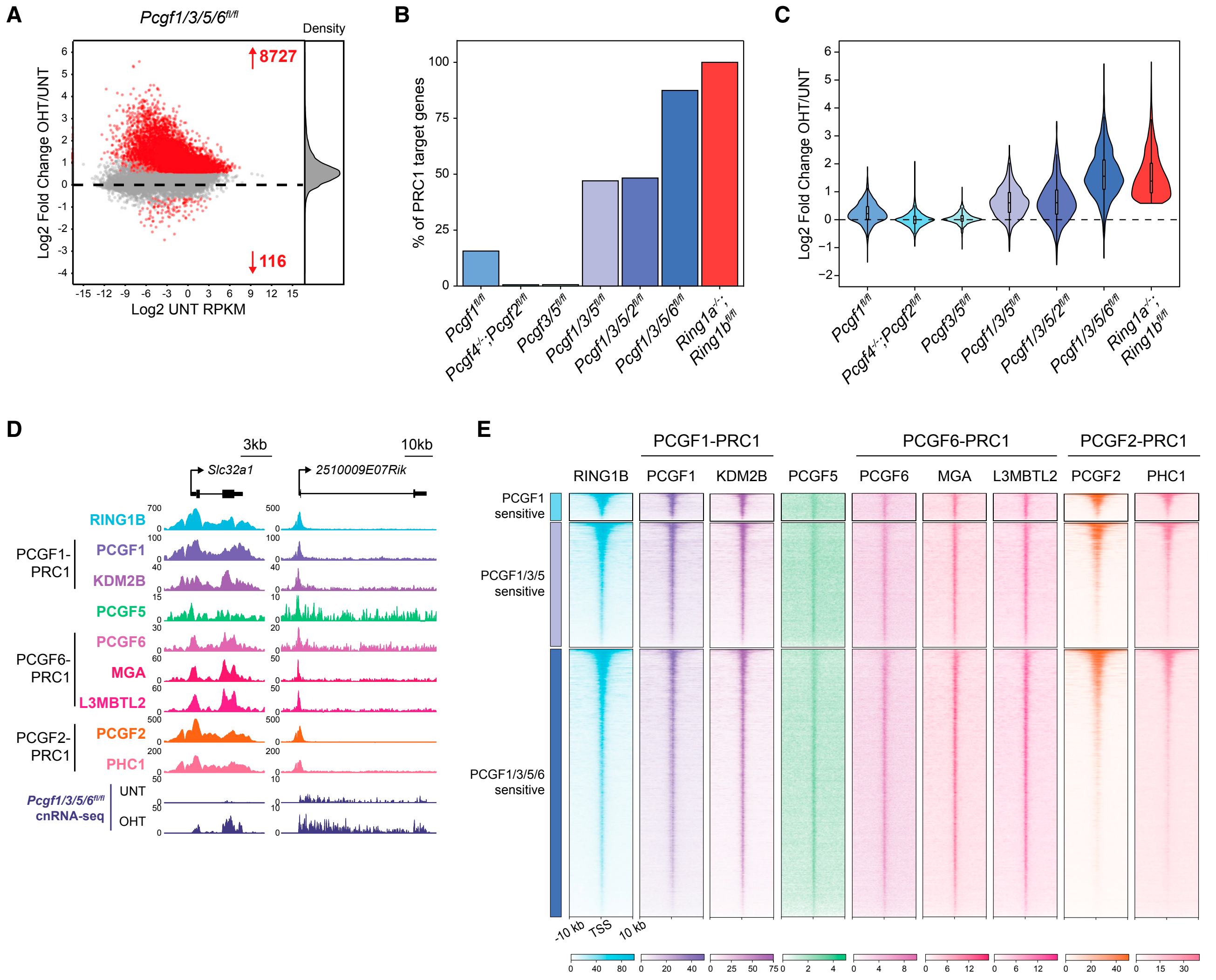

Figure 7. Polycomb-Mediated Gene Repression Relies on Synergy between Variant PRC1 Complexes

(A) An MA plot of log2-fold changes in gene expression (cnRNA-seq) in *Pcgf1/3/5/6*$^{fl/fl}$ ESCs following OHT treatment. Significant gene expression changes (p-adj < 0.05 and >1.5-fold) are shown in red.

(B) A bar plot comparing the proportion of PRC1 target genes that are derepressed (p-adj < 0.05 and >1.5-fold) across our complement of PRC1 perturbation ESCs.

(C) A violin plot comparing log2-fold changes in expression of PRC1 target genes across PRC1 perturbation ESCs.

(D) Genomic snapshots of two Polycomb target genes, showing ChIP-seq for RING1B and various specific components of variant and canonical PRC1 complexes, together with cnRNA-seq from *Pcgf1/3/5/6*$^{fl/fl}$ ESCs (UNT and OHT).

(E) Heatmaps of ChIP-seq data for PRC1 factors shown in (D) at promoters of variant PRC1-regulated genes (PCGF1-sensitive genes, n = 582; PCGF1/3/5-sensitive genes, n = 2,657; PCGF1/3/5/6-sensitive genes, n = 5,707) sorted by RING1B occupancy.

See also Figure S7.

contribute to chromosomal functions that are distinct from gene repression. For example, H2AK119ub1 has been proposed to contribute to DNA replication and repair (Bravo et al., 2015; Ismail et al., 2010; Lee et al., 2014; Uckelmann and Sixma, 2017), and the broad distribution of this modification may be ideally suited to regulate such processes. However, we also find that the blanket of H2AK119ub1 is specifically elevated during Xist-mediated chromosome silencing. This suggests that the level of pervasive H2AK119ub1 may also define whether it impinges on gene expression. In contrast to the low-level H2AK119ub1 throughout the genome, a pool of more punctate and highly enriched H2AK119ub1 deposited by PCGF1/3/5/6-PRC1 is associated with repression of Polycomb target genes. There remains an active debate around whether H2AK119ub1 directly contributes to gene repression. Some evidence suggests only a modest contribution (Cohen et al., 2018; Illingworth et al., 2015; Pengelly et al., 2015), although other studies suggest that H2AK119ub1 is essential for gene repression (Endoh et al., 2012). Here, we reveal that full derepression of Polycomb target genes is only achieved when all variant PRC1 complexes are

removed and H2AK119ub1 is erased, supporting the idea that H2AK119ub1 plays a central role in the process. Moving forward, an important challenge remains to examine in detail the molecular mechanisms by which H2AK119ub1 could contribute to gene repression. This may involve reader proteins that recognize H2AK119ub1 (Cooper et al., 2016; Kalb et al., 2014; Richly et al., 2010; Zhang et al., 2017), or H2AK119ub1 may directly counteract transcription, as suggested by recent observations using *in vitro* assays (Aihara et al., 2016).

Our functional interrogation of PRC1 activity indicates that synergy between variant PRC1 complexes is central to Polycomb-mediated gene repression. This was somewhat surprising given that previous studies of exogenously expressed epitope-tagged PRC1 proteins in human cells concluded that PCGF proteins occupy mutually exclusive regions of the genome, leading to the hypothesis that they had distinct roles in gene regulation (Gao et al., 2012). In contrast to these findings, our profiling of endogenous PCGF proteins indicates that variant PRC1 complexes largely co-occupy target sites, where they collaborate in depositing H2AK119ub1 and repressing Polycomb target genes. This suggests that, although biochemically distinct variant PRC1 complexes may have evolved unique targeting activities, which allow them to function at defined sites in the genome, for example, PCGF6-PRC1 at germline-specific genes (Endoh et al., 2017), they also retain a shared capacity to engage broadly with Polycomb target sites and deposit H2AK119ub1. Retaining this primordial function would provide the Polycomb repressive system with the versatility to accommodate new functional specialization via expansion during evolution, while at the same time enhancing the robustness of transcriptional repression at classical Polycomb target sites. This may be particularly relevant during early mammalian development, where the precise control of gene expression is paramount.

Canonical PRC1 complexes, which are recruited to chromatin via recognition of PRC2-dependent H3K27me3, have been proposed to elicit Polycomb-mediated gene repression (Schuettengruber et al., 2017; Simon and Kingston, 2013). Nevertheless, here, we find that conditional removal of canonical PRC1 complexes has a very minor effect on gene expression. This observation is consistent with previous reports that PRC2 loss has little effect on Polycomb target gene repression in ESCs (Riising et al., 2014). This raises an interesting question: why are canonical PRC1 complexes recruited to sites where variant PRC1 complexes predominate in gene repression? One explanation may be that canonical PRC1 complexes can mediate long-range interactions between Polycomb repressed genes (Boettiger et al., 2016; Bonev et al., 2017; Eagen et al., 2017; Isono et al., 2013; Kundu et al., 2017; Ogiyama et al., 2018; Saurin et al., 1998; Schoenfelder et al., 2015; Vieux-Rochas et al., 2015; Wani et al., 2016) that we speculate could reinforce the fidelity of variant PRC1-dependent gene repression, as previously suggested (Ogiyama et al., 2018). Efficient maintenance of repression would be beneficial during cellular differentiation or in development, where Polycomb systems must constrain transcriptional activation signals that vary in nature and magnitude. In agreement with these ideas, canonical PRC1 has been implicated in these processes (Cohen et al., 2018; Isono et al., 2013; Kloet et al., 2016; Kundu et al., 2017; Morey et al., 2015). Further support that canonical PRC1 complexes do not define Polycomb-mediated gene repression but instead may contribute to fidelity comes from the phenotype of mouse models, in which canonical PRC1 components are mutated or removed and display only mild or delayed defects in embryonic development (Akasaka et al., 2001; Coré et al., 1997; Forzati et al., 2012; Isono et al., 2005; Lau et al., 2017). In contrast, removal of variant PRC1 components leads to early embryonic lethality (Almeida et al., 2017; Blackledge et al., 2014; Endoh et al., 2017; Pirity et al., 2005; Washkowitz et al., 2015), presumably due to severe defects in developmental gene regulation.

An important conclusion from our discoveries is that variant PRC1 complexes, as opposed to canonical PRC1 complexes, define Polycomb-mediated gene repression in ESCs. This fundamental new distinction now paves the way to identify the detailed molecular mechanisms that govern Polycomb target site selection and repression by variant PRC1 complexes. Toward this goal, we and others have identified DNA binding domains in variant PRC1 complexes that appear to dynamically sample mammalian Polycomb responsive elements (Endoh et al., 2017; Farcas et al., 2012; He et al., 2013). We envisage that this could allow PRC1 to identify lowly transcribed or silent Polycomb target genes, at which to deposit H2AK119ub1 and elicit gene repression. Once deposited, H2AK119ub1 appears to stabilize the occupancy of PRC2 (Blackledge et al., 2014; Cooper et al., 2014, 2016; Kalb et al., 2014), which has also recently been shown to sample Polycomb responsive elements in ESCs (Li et al., 2017; Perino et al., 2018), resulting in the deposition of H3K27me3. This would support the recruitment of canonical PRC1 to sustain the fidelity of variant PRC1-dependent gene repression. Exploring these proposed models, discovering how H2AK119ub1 is mechanistically linked to gene repression, and understanding how widely these principles apply in other cellular contexts is now an important challenge for future work.

STAR★METHODS

Detailed methods are provided in the online version of this paper and include the following:

- KEY RESOURCES TABLE
- CONTACT FOR REAGENT AND RESOURCE SHARING
- EXPERIMENTAL MODEL AND SUBJECT DETAILS
- METHOD DETAILS
  - Genome engineering by CRISPR/Homology-Directed Repair (HDR)
  - Cell line generation
  - Calibrated ChIP-sequencing (cChIP-seq)
  - Native cChIP-sequencing
  - Calibrated nuclear RNA-sequencing (cnRNA-seq)
  - Preparation of nuclear and histone extracts and immunoblotting
  - Co-immunoprecipitation
- QUANTIFICATION AND STATISTICAL ANALYSIS
  - Massively parallel sequencing, *data processing and normalization*
  - Peak calling

- ○ Read count quantitation and analysis
- ○ Differential cChIP-seq enrichment and gene expression analysis
- ○ Gene annotation
- DATA AND SOFTWARE AVAILABILITY

SUPPLEMENTAL INFORMATION

Supplemental Information can be found online at https://doi.org/10.1016/j.molcel.2019.03.024.

ACKNOWLEDGMENTS

Work in the Klose lab is supported by the Wellcome Trust (209400/Z/17/Z), the European Research Council (681440), and the Lister Institute of Preventive Medicine. We would like to thank Neil Brockdorff for kindly sharing with us the inducible *Xist* transgene model cell line and Guifeng Wei for help with bioinformatics analysis using allele-specific SNPs. We are grateful to Amanda Williams at the Department of Zoology, Oxford, for sequencing support on the NextSeq 500. We also thank Paula Dobrinic and Miles Huseyin for critical reading of the manuscript.

AUTHOR CONTRIBUTIONS

Conceptualization, N.A.F., N.P.B., A.M.F., H.K., and R.J.K.; Methodology, N.A.F., N.P.B., M.N., S.I., and Y.K.; Investigation, N.A.F., N.P.B., and A.M.F.; Formal Analysis, N.A.F. and H.W.K.; Resources, M.N., S.I., Y.K., and H.W.K.; Writing – Original Draft, N.A.F., N.P.B., and R.J.K.; Writing – Review and Editing, N.A.F., N.P.B., H.W.K., and R.J.K.; Funding Acquisition, H.K. and R.J.K.; Supervision, H.K. and R.J.K.

DECLARATION OF INTERESTS

The authors declare no competing interests.

Received: September 18, 2018
Revised: February 4, 2019
Accepted: March 21, 2019
Published: April 24, 2019

REFERENCES

Aihara, H., Nakagawa, T., Mizusaki, H., Yoneda, M., Kato, M., Doiguchi, M., Imamura, Y., Higashi, M., Ikura, T., Hayashi, T., et al. (2016). Histone H2A T120 phosphorylation promotes oncogenic transformation via upregulation of cyclin D1. Mol. Cell *64*, 176–188.

Akasaka, T., van Lohuizen, M., van der Lugt, N., Mizutani-Koseki, Y., Kanno, M., Taniguchi, M., Vidal, M., Alkema, M., Berns, A., and Koseki, H. (2001). Mice doubly deficient for the Polycomb Group genes Mel18 and Bmi1 reveal synergy and requirement for maintenance but not initiation of Hox gene expression. Development *128*, 1587–1597.

Albright, S.C., Nelson, P.P., and Garrard, W.T. (1979). Histone molar ratios among different electrophoretic forms of mono- and dinucleosomes. J. Biol. Chem. *254*, 1065–1073.

Almeida, M., Pintacuda, G., Masui, O., Koseki, Y., Gdula, M., Cerase, A., Brown, D., Mould, A., Innocent, C., Nakayama, M., et al. (2017). PCGF3/5-PRC1 initiates Polycomb recruitment in X chromosome inactivation. Science *356*, 1081–1084.

Atlasi, Y., and Stunnenberg, H.G. (2017). The interplay of epigenetic marks during stem cell differentiation and development. Nat. Rev. Genet. *18*, 643–658.

Atsuta, T., Fujimura, S., Moriya, H., Vidal, M., Akasaka, T., and Koseki, H. (2001). Production of monoclonal antibodies against mammalian Ring1B proteins. Hybridoma *20*, 43–46.

Blackledge, N.P., Farcas, A.M., Kondo, T., King, H.W., McGouran, J.F., Hanssen, L.L., Ito, S., Cooper, S., Kondo, K., Koseki, Y., et al. (2014). Variant PRC1 complex-dependent H2A ubiquitylation drives PRC2 recruitment and polycomb domain formation. Cell *157*, 1445–1459.

Blackledge, N.P., Rose, N.R., and Klose, R.J. (2015). Targeting Polycomb systems to regulate gene expression: modifications to a complex story. Nat. Rev. Mol. Cell Biol. *16*, 643–649.

Boettiger, A.N., Bintu, B., Moffitt, J.R., Wang, S., Beliveau, B.J., Fudenberg, G., Imakaev, M., Mirny, L.A., Wu, C.T., and Zhuang, X. (2016). Super-resolution imaging reveals distinct chromatin folding for different epigenetic states. Nature *529*, 418–422.

Bonev, B., Mendelson Cohen, N., Szabo, Q., Fritsch, L., Papadopoulos, G.L., Lubling, Y., Xu, X., Lv, X., Hugnot, J.P., Tanay, A., et al. (2017). Multiscale 3D genome rewiring during mouse neural development. Cell *171*, 557–572.e24.

Bonhoure, N., Bounova, G., Bernasconi, D., Praz, V., Lammers, F., Canella, D., Willis, I.M., Herr, W., Hernandez, N., and Delorenzi, M.; CycliX Consortium (2014). Quantifying ChIP-seq data: a spiking method providing an internal reference for sample-to-sample normalization. Genome Res. *24*, 1157–1168.

Bousard, A., Raposo, A.C., Zylicz, J.J., Picard, C., Pires, V.B., Qi, Y., Syx, L., Chang, H.Y., Heard, E., and da Rocha, S.T. (2018). Exploring the role of Polycomb recruitment in Xist-mediated silencing of the X chromosome in ES cells. bioRxiv. https://doi.org/10.1101/495739.

Bravo, M., Nicolini, F., Starowicz, K., Barroso, S., Calés, C., Aguilera, A., and Vidal, M. (2015). Polycomb RING1A- and RING1B-dependent histone H2A monoubiquitylation at pericentromeric regions promotes S-phase progression. J. Cell Sci. *128*, 3660–3671.

Cao, R., Wang, L., Wang, H., Xia, L., Erdjument-Bromage, H., Tempst, P., Jones, R.S., and Zhang, Y. (2002). Role of histone H3 lysine 27 methylation in Polycomb-group silencing. Science *298*, 1039–1043.

Cohen, I., Zhao, D., Bar, C., Valdes, V.J., Dauber-Decker, K.L., Nguyen, M.B., Nakayama, M., Rendl, M., Bickmore, W.A., Koseki, H., et al. (2018). PRC1 fine-tunes gene repression and activation to safeguard skin development and stem cell specification. Cell Stem Cell *22*, 726–739.e7.

Conway, E., Jerman, E., Healy, E., Ito, S., Holoch, D., Oliviero, G., Deevy, O., Glancy, E., Fitzpatrick, D.J., Mucha, M., et al. (2018). A family of vertebrate-specific polycombs encoded by the LCOR/LCORL genes balance PRC2 subtype activities. Mol. Cell *70*, 408–421.e8.

Cooper, S., Dienstbier, M., Hassan, R., Schermelleh, L., Sharif, J., Blackledge, N.P., De Marco, V., Elderkin, S., Koseki, H., Klose, R., et al. (2014). Targeting polycomb to pericentric heterochromatin in embryonic stem cells reveals a role for H2AK119u1 in PRC2 recruitment. Cell Rep. *7*, 1456–1470.

Cooper, S., Grijzenhout, A., Underwood, E., Ancelin, K., Zhang, T., Nesterova, T.B., Anil-Kirmizitas, B., Bassett, A., Kooistra, S.M., Agger, K., et al. (2016). Jarid2 binds mono-ubiquitylated H2A lysine 119 to mediate crosstalk between Polycomb complexes PRC1 and PRC2. Nat. Commun. *7*, 13661.

Coré, N., Bel, S., Gaunt, S.J., Aurrand-Lions, M., Pearce, J., Fisher, A., and Djabali, M. (1997). Altered cellular proliferation and mesoderm patterning in Polycomb-M33-deficient mice. Development *124*, 721–729.

Creppe, C., Palau, A., Malinverni, R., Valero, V., and Buschbeck, M. (2014). A Cbx8-containing polycomb complex facilitates the transition to gene activation during ES cell differentiation. PLoS Genet. *10*, e1004851.

Czermin, B., Melfi, R., McCabe, D., Seitz, V., Imhof, A., and Pirrotta, V. (2002). Drosophila enhancer of Zeste/ESC complexes have a histone H3 methyltransferase activity that marks chromosomal Polycomb sites. Cell *111*, 185–196.

Di Croce, L., and Helin, K. (2013). Transcriptional regulation by Polycomb group proteins. Nat. Struct. Mol. Biol. *20*, 1147–1155.

Dimitrova, E., Kondo, T., Feldmann, A., Nakayama, M., Koseki, Y., Konietzny, R., Kessler, B.M., Koseki, H., and Klose, R.J. (2018). FBXL19 recruits CDK-Mediator to CpG islands of developmental genes priming them for activation during lineage commitment. eLife *7*, e37084.

Dobin, A., Davis, C.A., Schlesinger, F., Drenkow, J., Zaleski, C., Jha, S., Batut, P., Chaisson, M., and Gingeras, T.R. (2013). STAR: ultrafast universal RNA-seq aligner. Bioinformatics *29*, 15–21.

Eagen, K.P., Aiden, E.L., and Kornberg, R.D. (2017). Polycomb-mediated chromatin loops revealed by a subkilobase-resolution chromatin interaction map. Proc. Natl. Acad. Sci. USA *114*, 8764–8769.

Endoh, M., Endo, T.A., Endoh, T., Fujimura, Y., Ohara, O., Toyoda, T., Otte, A.P., Okano, M., Brockdorff, N., Vidal, M., and Koseki, H. (2008). Polycomb group proteins Ring1A/B are functionally linked to the core transcriptional regulatory circuitry to maintain ES cell identity. Development *135*, 1513–1524.

Endoh, M., Endo, T.A., Endoh, T., Isono, K., Sharif, J., Ohara, O., Toyoda, T., Ito, T., Eskeland, R., Bickmore, W.A., et al. (2012). Histone H2A mono-ubiquitination is a crucial step to mediate PRC1-dependent repression of developmental genes to maintain ES cell identity. PLoS Genet. *8*, e1002774.

Endoh, M., Endo, T.A., Shinga, J., Hayashi, K., Farcas, A., Ma, K.-W., Ito, S., Sharif, J., Endoh, T., Onaga, N., et al. (2017). PCGF6-PRC1 suppresses premature differentiation of mouse embryonic stem cells by regulating germ cell-related genes. eLife *6*, e21064.

Eskeland, R., Leeb, M., Grimes, G.R., Kress, C., Boyle, S., Sproul, D., Gilbert, N., Fan, Y., Skoultchi, A.I., Wutz, A., and Bickmore, W.A. (2010). Ring1B compacts chromatin structure and represses gene expression independent of histone ubiquitination. Mol. Cell *38*, 452–464.

Farcas, A.M., Blackledge, N.P., Sudbery, I., Long, H.K., McGouran, J.F., Rose, N.R., Lee, S., Sims, D., Cerase, A., Sheahan, T.W., et al. (2012). KDM2B links the Polycomb repressive complex 1 (PRC1) to recognition of CpG islands. eLife *1*, e00205.

Forzati, F., Federico, A., Pallante, P., Abbate, A., Esposito, F., Malapelle, U., Sepe, R., Palma, G., Troncone, G., Scarfò, M., et al. (2012). CBX7 is a tumor suppressor in mice and humans. J. Clin. Invest. *122*, 612–623.

Francis, N.J., Kingston, R.E., and Woodcock, C.L. (2004). Chromatin compaction by a polycomb group protein complex. Science *306*, 1574–1577.

Frangini, A., Sjöberg, M., Roman-Trufero, M., Dharmalingam, G., Haberle, V., Bartke, T., Lenhard, B., Malumbres, M., Vidal, M., and Dillon, N. (2013). The aurora B kinase and the polycomb protein ring1B combine to regulate active promoters in quiescent lymphocytes. Mol. Cell *51*, 647–661.

Gao, Z., Zhang, J., Bonasio, R., Strino, F., Sawai, A., Parisi, F., Kluger, Y., and Reinberg, D. (2012). PCGF homologs, CBX proteins, and RYBP define functionally distinct PRC1 family complexes. Mol. Cell *45*, 344–356.

Gao, Z., Lee, P., Stafford, J.M., von Schimmelmann, M., Schaefer, A., and Reinberg, D. (2014). An AUTS2-Polycomb complex activates gene expression in the CNS. Nature *516*, 349–354.

Gaytán de Ayala Alonso, A., Gutiérrez, L., Fritsch, C., Papp, B., Beuchle, D., and Müller, J. (2007). A genetic screen identifies novel polycomb group genes in Drosophila. Genetics *176*, 2099–2108.

Gil, J., Bernard, D., and Peters, G. (2005). Role of polycomb group proteins in stem cell self-renewal and cancer. DNA Cell Biol. *24*, 117–125.

Grau, D.J., Chapman, B.A., Garlick, J.D., Borowsky, M., Francis, N.J., and Kingston, R.E. (2011). Compaction of chromatin by diverse Polycomb group proteins requires localized regions of high charge. Genes Dev. *25*, 2210–2221.

Hauri, S., Comoglio, F., Seimiya, M., Gerstung, M., Glatter, T., Hansen, K., Aebersold, R., Paro, R., Gstaiger, M., and Beisel, C. (2016). A high-density map for navigating the human polycomb complexome. Cell Rep. *17*, 583–595.

He, J., Shen, L., Wan, M., Taranova, O., Wu, H., and Zhang, Y. (2013). Kdm2b maintains murine embryonic stem cell status by recruiting PRC1 complex to CpG islands of developmental genes. Nat. Cell Biol. *15*, 373–384.

Heinz, S., Benner, C., Spann, N., Bertolino, E., Lin, Y.C., Laslo, P., Cheng, J.X., Murre, C., Singh, H., and Glass, C.K. (2010). Simple combinations of lineage-determining transcription factors prime cis-regulatory elements required for macrophage and B cell identities. Mol. Cell *38*, 576–589.

Hu, B., Petela, N., Kurze, A., Chan, K.L., Chapard, C., and Nasmyth, K. (2015). Biological chromodynamics: a general method for measuring protein occupancy across the genome by calibrating ChIP-seq. Nucleic Acids Res. *43*, e132.

Huber, W., Carey, V.J., Gentleman, R., Anders, S., Carlson, M., Carvalho, B.S., Bravo, H.C., Davis, S., Gatto, L., Girke, T., et al. (2015). Orchestrating high-throughput genomic analysis with Bioconductor. Nat. Methods *12*, 115–121.

Illingworth, R.S., Moffat, M., Mann, A.R., Read, D., Hunter, C.J., Pradeepa, M.M., Adams, I.R., and Bickmore, W.A. (2015). The E3 ubiquitin ligase activity of RING1B is not essential for early mouse development. Genes Dev. *29*, 1897–1902.

Ismail, I.H., Andrin, C., McDonald, D., and Hendzel, M.J. (2010). BMI1-mediated histone ubiquitylation promotes DNA double-strand break repair. J. Cell Biol. *191*, 45–60.

Isono, K., Fujimura, Y., Shinga, J., Yamaki, M., O-Wang, J., Takihara, Y., Murahashi, Y., Takada, Y., Mizutani-Koseki, Y., and Koseki, H. (2005). Mammalian polyhomeotic homologues Phc2 and Phc1 act in synergy to mediate polycomb repression of Hox genes. Mol. Cell. Biol. *25*, 6694–6706.

Isono, K., Endo, T.A., Ku, M., Yamada, D., Suzuki, R., Sharif, J., Ishikura, T., Toyoda, T., Bernstein, B.E., and Koseki, H. (2013). SAM domain polymerization links subnuclear clustering of PRC1 to gene silencing. Dev. Cell *26*, 565–577.

Jürgens, G. (1985). A group of genes controlling the spatial expression of the bithorax complex in *Drosophila*. Nature *316*, 153–155.

Kahn, T.G., Dorafshan, E., Schultheis, D., Zare, A., Stenberg, P., Reim, I., Pirrotta, V., and Schwartz, Y.B. (2016). Interdependence of PRC1 and PRC2 for recruitment to Polycomb response elements. Nucleic Acids Res. *44*, 10132–10149.

Kalb, R., Latwiel, S., Baymaz, H.I., Jansen, P.W., Müller, C.W., Vermeulen, M., and Müller, J. (2014). Histone H2A monoubiquitination promotes histone H3 methylation in Polycomb repression. Nat. Struct. Mol. Biol. *21*, 569–571.

Kent, W.J., Sugnet, C.W., Furey, T.S., Roskin, K.M., Pringle, T.H., Zahler, A.M., and Haussler, D. (2002). The human genome browser at UCSC. Genome Res. *12*, 996–1006.

Kim, C.A., Gingery, M., Pilpa, R.M., and Bowie, J.U. (2002). The SAM domain of polyhomeotic forms a helical polymer. Nat. Struct. Biol. *9*, 453–457.

Kloet, S.L., Makowski, M.M., Baymaz, H.I., van Voorthuijsen, L., Karemaker, I.D., Santanach, A., Jansen, P.W.T.C., Di Croce, L., and Vermeulen, M. (2016). The dynamic interactome and genomic targets of Polycomb complexes during stem-cell differentiation. Nat. Struct. Mol. Biol. *23*, 682–690.

Krueger, F., and Andrews, S.R. (2016). SNPsplit: allele-specific splitting of alignments between genomes with known SNP genotypes. F1000Res. *5*, 1479.

Kundu, S., Ji, F., Sunwoo, H., Jain, G., Lee, J.T., Sadreyev, R.I., Dekker, J., and Kingston, R.E. (2017). Polycomb repressive complex 1 generates discrete compacted domains that change during differentiation. Mol. Cell *65*, 432–446.e5.

Kuzmichev, A., Nishioka, K., Erdjument-Bromage, H., Tempst, P., and Reinberg, D. (2002). Histone methyltransferase activity associated with a human multiprotein complex containing the Enhancer of Zeste protein. Genes Dev. *16*, 2893–2905.

Lai, W.K.M., and Pugh, B.F. (2017). Understanding nucleosome dynamics and their links to gene expression and DNA replication. Nat. Rev. Mol. Cell Biol. *18*, 548–562.

Langmead, B., and Salzberg, S.L. (2012). Fast gapped-read alignment with Bowtie 2. Nat. Methods *9*, 357–359.

Lau, M.S., Schwartz, M.G., Kundu, S., Savol, A.J., Wang, P.I., Marr, S.K., Grau, D.J., Schorderet, P., Sadreyev, R.I., Tabin, C.J., and Kingston, R.E. (2017). Mutation of a nucleosome compaction region disrupts Polycomb-mediated axial patterning. Science *355*, 1081–1084.

Lavigne, M., Francis, N.J., King, I.F., and Kingston, R.E. (2004). Propagation of silencing; recruitment and repression of naive chromatin in trans by polycomb repressed chromatin. Mol. Cell *13*, 415–425.

Lee, H.S., Lee, S.A., Hur, S.K., Seo, J.W., and Kwon, J. (2014). Stabilization and targeting of INO80 to replication forks by BAP1 during normal DNA synthesis. Nat. Commun. *5*, 5128.

Lee, H.G., Kahn, T.G., Simcox, A., Schwartz, Y.B., and Pirrotta, V. (2015). Genome-wide activities of Polycomb complexes control pervasive transcription. Genome Res. *25*, 1170–1181.

Lewis, E.B. (1978). A gene complex controlling segmentation in Drosophila. Nature *276*, 565–570.

Lex, A., Gehlenborg, N., Strobelt, H., Vuillemot, R., and Pfister, H. (2014). UpSet: visualization of intersecting sets. IEEE Trans. Vis. Comput. Graph. *20*, 1983–1992.

Li, H., Handsaker, B., Wysoker, A., Fennell, T., Ruan, J., Homer, N., Marth, G., Abecasis, G., and Durbin, R.; 1000 Genome Project Data Processing Subgroup (2009). The Sequence Alignment/Map format and SAMtools. Bioinformatics *25*, 2078–2079.

Li, H., Liefke, R., Jiang, J., Kurland, J.V., Tian, W., Deng, P., Zhang, W., He, Q., Patel, D.J., Bulyk, M.L., et al. (2017). Polycomb-like proteins link the PRC2 complex to CpG islands. Nature *549*, 287–291.

Long, H.K., Sims, D., Heger, A., Blackledge, N.P., Kutter, C., Wright, M.L., Grützner, F., Odom, D.T., Patient, R., Ponting, C.P., and Klose, R.J. (2013). Epigenetic conservation at gene regulatory elements revealed by non-methylated DNA profiling in seven vertebrates. eLife *2*, e00348.

Love, M.I., Huber, W., and Anders, S. (2014). Moderated estimation of fold change and dispersion for RNA-seq data with DESeq2. Genome Biol. *15*, 550.

Margueron, R., Justin, N., Ohno, K., Sharpe, M.L., Son, J., Drury, W.J., 3rd, Voigt, P., Martin, S.R., Taylor, W.R., De Marco, V., et al. (2009). Role of the polycomb protein EED in the propagation of repressive histone marks. Nature *461*, 762–767.

Matsui, S.I., Seon, B.K., and Sandberg, A.A. (1979). Disappearance of a structural chromatin protein A24 in mitosis: implications for molecular basis of chromatin condensation. Proc. Natl. Acad. Sci. USA *76*, 6386–6390.

Morey, L., Aloia, L., Cozzuto, L., Benitah, S.A., and Di Croce, L. (2013). RYBP and Cbx7 define specific biological functions of polycomb complexes in mouse embryonic stem cells. Cell Rep. *3*, 60–69.

Morey, L., Santanach, A., Blanco, E., Aloia, L., Nora, E.P., Bruneau, B.G., and Di Croce, L. (2015). Polycomb regulates mesoderm cell fate-specification in embryonic stem cells through activation and repression mechanisms. Cell Stem Cell *17*, 300–315.

Müller, J., Hart, C.M., Francis, N.J., Vargas, M.L., Sengupta, A., Wild, B., Miller, E.L., O'Connor, M.B., Kingston, R.E., and Simon, J.A. (2002). Histone methyltransferase activity of a Drosophila Polycomb group repressor complex. Cell *111*, 197–208.

Nesterova, T.B., Wei, G., Coker, H., Pintacuda, G., Bowness, J.S., Zhang, T., Almeida, M., Bloechl, B., Moindrot, B., Carter, E.J., et al. (2018). Systematic allelic analysis defines the interplay of key pathways in X chromosome inactivation. bioRxiv. https://doi.org/10.1101/477232.

Ogiyama, Y., Schuettengruber, B., Papadopoulos, G.L., Chang, J.M., and Cavalli, G. (2018). Polycomb-dependent chromatin looping contributes to gene silencing during Drosophila development. Mol. Cell *71*, 73–88.e5.

Orlando, D.A., Chen, M.W., Brown, V.E., Solanki, S., Choi, Y.J., Olson, E.R., Fritz, C.C., Bradner, J.E., and Guenther, M.G. (2014). Quantitative ChIP-seq normalization reveals global modulation of the epigenome. Cell Rep. *9*, 1163–1170.

Pengelly, A.R., Kalb, R., Finkl, K., and Müller, J. (2015). Transcriptional repression by PRC1 in the absence of H2A monoubiquitylation. Genes Dev. *29*, 1487–1492.

Perino, M., van Mierlo, G., Karemaker, I.D., van Genesen, S., Vermeulen, M., Marks, H., van Heeringen, S.J., and Veenstra, G.J.C. (2018). MTF2 recruits Polycomb repressive complex 2 by helical-shape-selective DNA binding. Nat. Genet. *50*, 1002–1010.

Pintacuda, G., Wei, G., Roustan, C., Kirmizitas, B.A., Solcan, N., Cerase, A., Castello, A., Mohammed, S., Moindrot, B., Nesterova, T.B., et al. (2017). hnRNPK recruits PCGF3/5-PRC1 to the Xist RNA B-repeat to establish Polycomb-mediated chromosomal silencing. Mol. Cell *68*, 955–969.e10.

Pirity, M.K., Locker, J., and Schreiber-Agus, N. (2005). Rybp/DEDAF is required for early postimplantation and for central nervous system development. Mol. Cell. Biol. *25*, 7193–7202.

Poux, S., Melfi, R., and Pirrotta, V. (2001). Establishment of Polycomb silencing requires a transient interaction between PC and ESC. Genes Dev. *15*, 2509–2514.

Quinlan, A.R. (2014). BEDTools: the Swiss-army tool for genome feature analysis. Curr. Protoc. Bioinformatics *47*, 11.12.1–11.12.34.

Ramírez, F., Dündar, F., Diehl, S., Grüning, B.A., and Manke, T. (2014). deepTools: a flexible platform for exploring deep-sequencing data. Nucleic Acids Res. *42*, W187–W191.

Richly, H., Rocha-Viegas, L., Ribeiro, J.D., Demajo, S., Gundem, G., Lopez-Bigas, N., Nakagawa, T., Rospert, S., Ito, T., and Di Croce, L. (2010). Transcriptional activation of polycomb-repressed genes by ZRF1. Nature *468*, 1124–1128.

Riising, E.M., Comet, I., Leblanc, B., Wu, X., Johansen, J.V., and Helin, K. (2014). Gene silencing triggers polycomb repressive complex 2 recruitment to CpG islands genome wide. Mol. Cell *55*, 347–360.

Rose, N.R., King, H.W., Blackledge, N.P., Fursova, N.A., Ember, K.J., Fischer, R., Kessler, B.M., and Klose, R.J. (2016). RYBP stimulates PRC1 to shape chromatin-based communication between Polycomb repressive complexes. eLife *5*, e18591.

Saurin, A.J., Shiels, C., Williamson, J., Satijn, D.P., Otte, A.P., Sheer, D., and Freemont, P.S. (1998). The human polycomb group complex associates with pericentromeric heterochromatin to form a novel nuclear domain. J. Cell Biol. *142*, 887–898.

Schoenfelder, S., Sugar, R., Dimond, A., Javierre, B.M., Armstrong, H., Mifsud, B., Dimitrova, E., Matheson, L., Tavares-Cadete, F., Furlan-Magaril, M., et al. (2015). Polycomb repressive complex PRC1 spatially constrains the mouse embryonic stem cell genome. Nat. Genet. *47*, 1179–1186.

Schuettengruber, B., Bourbon, H.M., Di Croce, L., and Cavalli, G. (2017). Genome regulation by Polycomb and trithorax: 70 years and counting. Cell *171*, 34–57.

Shao, Z., Raible, F., Mollaaghababa, R., Guyon, J.R., Wu, C.T., Bender, W., and Kingston, R.E. (1999). Stabilization of chromatin structure by PRC1, a Polycomb complex. Cell *98*, 37–46.

Simon, J.A., and Kingston, R.E. (2013). Occupying chromatin: Polycomb mechanisms for getting to genomic targets, stopping transcriptional traffic, and staying put. Mol. Cell *49*, 808–824.

Smits, A.H., Jansen, P.W., Poser, I., Hyman, A.A., and Vermeulen, M. (2013). Stoichiometry of chromatin-associated protein complexes revealed by label-free quantitative mass spectrometry-based proteomics. Nucleic Acids Res. *41*, e28.

Stielow, B., Finkernagel, F., Stiewe, T., Nist, A., and Suske, G. (2018). MGA, L3MBTL2 and E2F6 determine genomic binding of the non-canonical Polycomb repressive complex PRC1.6. PLoS Genet. *14*, e1007193.

Suzuki, A., Hirasaki, M., Hishida, T., Wu, J., Okamura, D., Ueda, A., Nishimoto, M., Nakachi, Y., Mizuno, Y., Okazaki, Y., et al. (2016). Loss of MAX results in meiotic entry in mouse embryonic and germline stem cells. Nat. Commun. *7*, 11056.

Taherbhoy, A.M., Huang, O.W., and Cochran, A.G. (2015). BMI1-RING1B is an autoinhibited RING E3 ubiquitin ligase. Nat. Commun. *6*, 7621.

Tarasov, A., Vilella, A.J., Cuppen, E., Nijman, I.J., and Prins, P. (2015). Sambamba: fast processing of NGS alignment formats. Bioinformatics *31*, 2032–2034.

Taruttis, F., Feist, M., Schwarzfischer, P., Gronwald, W., Kube, D., Spang, R., and Engelmann, J.C. (2017). External calibration with Drosophila whole-cell spike-ins delivers absolute mRNA fold changes from human RNA-seq and qPCR data. Biotechniques *62*, 53–61.

Tavares, L., Dimitrova, E., Oxley, D., Webster, J., Poot, R., Demmers, J., Bezstarosti, K., Taylor, S., Ura, H., Koide, H., et al. (2012). RYBP-PRC1 complexes mediate H2A ubiquitylation at polycomb target sites independently of PRC2 and H3K27me3. Cell *148*, 664–678.

Uckelmann, M., and Sixma, T.K. (2017). Histone ubiquitination in the DNA damage response. DNA Repair (Amst.) *56*, 92–101.

Vieux-Rochas, M., Fabre, P.J., Leleu, M., Duboule, D., and Noordermeer, D. (2015). Clustering of mammalian Hox genes with other H3K27me3 targets within an active nuclear domain. Proc. Natl. Acad. Sci. USA *112*, 4672–4677.

Wang, H., Wang, L., Erdjument-Bromage, H., Vidal, M., Tempst, P., Jones, R.S., and Zhang, Y. (2004a). Role of histone H2A ubiquitination in Polycomb silencing. Nature *431*, 873–878.

Wang, L., Brown, J.L., Cao, R., Zhang, Y., Kassis, J.A., and Jones, R.S. (2004b). Hierarchical recruitment of polycomb group silencing complexes. Mol. Cell *14*, 637–646.

Wani, A.H., Boettiger, A.N., Schorderet, P., Ergun, A., Münger, C., Sadreyev, R.I., Zhuang, X., Kingston, R.E., and Francis, N.J. (2016). Chromatin topology is coupled to Polycomb group protein subnuclear organization. Nat. Commun. *7*, 10291.

Washkowitz, A.J., Schall, C., Zhang, K., Wurst, W., Floss, T., Mager, J., and Papaioannou, V.E. (2015). Mga is essential for the survival of pluripotent cells during peri-implantation development. Development *142*, 31–40.

Yao, M., Zhou, X., Zhou, J., Gong, S., Hu, G., Li, J., Huang, K., Lai, P., Shi, G., Hutchins, A.P., et al. (2018). PCGF5 is required for neural differentiation of embryonic stem cells. Nat. Commun. *9*, 1463.

Zdzieblo, D., Li, X., Lin, Q., Zenke, M., Illich, D.J., Becker, M., and Müller, A.M. (2014). Pcgf6, a polycomb group protein, regulates mesodermal lineage differentiation in murine ESCs and functions in iPS reprogramming. Stem Cells *32*, 3112–3125.

Zhang, Y., Liu, T., Meyer, C.A., Eeckhoute, J., Johnson, D.S., Bernstein, B.E., Nusbaum, C., Myers, R.M., Brown, M., Li, W., and Liu, X.S. (2008). Model-based analysis of ChIP-seq (MACS). Genome Biol. *9*, R137.

Zhang, Z., Jones, A.E., Wu, W., Kim, J., Kang, Y., Bi, X., Gu, Y., Popov, I.K., Renfrow, M.B., Vassylyeva, M.N., et al. (2017). Role of remodeling and spacing factor 1 in histone H2A ubiquitination-mediated gene silencing. Proc. Natl. Acad. Sci. USA *114*, E7949–E7958.

Zhao, W., Huang, Y., Zhang, J., Liu, M., Ji, H., Wang, C., Cao, N., Li, C., Xia, Y., Jiang, Q., and Qin, J. (2017a). Polycomb group RING finger proteins 3/5 activate transcription via an interaction with the pluripotency factor Tex10 in embryonic stem cells. J. Biol. Chem. *292*, 21527–21537.

Zhao, W., Tong, H., Huang, Y., Yan, Y., Teng, H., Xia, Y., Jiang, Q., and Qin, J. (2017b). Essential role for Polycomb group protein Pcgf6 in embryonic stem cell maintenance and a noncanonical Polycomb repressive complex 1 (PRC1) integrity. J. Biol. Chem. *292*, 2773–2784.

STAR★METHODS

KEY RESOURCES TABLE

REAGENT or RESOURCE	SOURCE	IDENTIFIER
**Antibodies**		
Rabbit monoclonal anti-H2AK119ub1	Cell Signaling Technology	Cat# 8240; RRID:AB_10891618
Rabbit polyclonal anti-H3K27me3	In house (Rose et al., 2016)	N/A
Rabbit monoclonal anti-RING1B (ChIP)	Cell Signaling Technology	Cat# 5694; RRID:AB_10705604
Mouse monoclonal anti-RING1B (WB)	Atsuta et al., 2001	N/A
Rabbit monoclonal anti-SUZ12	Cell Signaling Technology	Cat# 3737; RRID:AB_2196850
Mouse monoclonal anti-H3	Cell Signaling Technology	Cat# 3638; RRID:AB_1642229
Mouse monoclonal anti-H4	Cell Signaling Technology	Cat# 2935; RRID:AB_1147658
Rabbit polyclonal anti-PCGF6	LifeSpan BioSciences	Cat# LS-C482495
Rabbit monoclonal anti-PCGF3+PCGF5	Abcam	Cat# ab201510
Rabbit polyclonal anti-PCGF2 (Mel-18)	Santa Cruz	Cat# sc-10744; RRID:AB_2267885
Rabbit polyclonal anti-PCGF1	In house (Blackledge et al., 2014)	N/A
Anti-PHC1	Cell Signaling Technology	Cat# 13768; RRID:AB_2716803
Anti-CBX7 (WB)	Millipore	Cat# 07-981; RRID:AB_10807034
Anti-CBX7 (ChIP)	Abcam	Cat# ab21873; RRID:AB_726005
Rabbit monoclonal anti-BRG1	Abcam	Cat# ab110641; RRID:AB_10861578
Rabbit polyclonal anti-H2A	Millipore	Cat# 07-146; RRID:AB_11212920
Rabbit monoclonal anti-EZH2	Cell Signaling Technology	Cat# 5246; RRID:AB_10694683
**Chemicals, Peptides, and Recombinant Proteins**		
Methanol-free Formaldehyde	Thermo Fisher Scientific	Cat# 10751395
DSG	Thermo Fisher Scientific	Cat# 11836794
Micrococcal Nuclease	Thermo Fisher Scientific	Cat# EN0181
Proteinase K	Sigma	Cat# P4850
(Z)-4-Hydroxytamoxifen	Sigma	Cat# H7904
SensiMix SYBR No-ROX Kit	Bioline	Cat# QT650-20
TRIzol reagent	Thermo Fisher Scientific	Cat# 15596018
Lipofectamine 3000	Thermo Fisher Scientific	Cat# L3000015
**Critical Commercial Assays**		
NEBNext® Multiplex Oligos for Illumina® (Index Primers Set 1)	NEB	Cat# E7335L
NEBNext® Multiplex Oligos for Illumina® (Index Primers Set 2)	NEB	Cat# E7500L
NEBNext® Ultra DNA Library Prep Kit for Illumina®	NEB	Cat# E7370L
NEBNext® Ultra Directional RNA Library Prep Kit for Illumina®	NEB	Cat# E7420S
NEBNext rRNA Depletion Kit (HumanMouseRat)	NEB	Cat# E6310L
High Sensitivity DNA Kit for Bioanalyzer	Agilent	Cat# 5067-4626
RNA Pico 6000 Kit for Bioanalyzer	Agilent	Cat# 5067-1513
TURBO DNA-*free* Kit	Thermo Fisher Scientific	Cat# AM1907
Gibson Assembly Master Mix	NEB	Cat# E2611L
NextSeq® 500/550 High Output Kit v2 (150 cycles)	Illumina	Cat# FC-404-2002
NextSeq 500/550 High Output v2 Kit (75 cycles)	Illumina	Cat# FC-404-2005
KAPA Illumina DNA Standards	Roche	Cat# 7960387001
ChIP DNA Clean and Concentrator	Zymo Research	Cat# D5205
**Deposited Data**		
All NGS data, GEO SuperSeries	This study	GEO: GSE119620

(*Continued on next page*)

Continued		
REAGENT or RESOURCE	SOURCE	IDENTIFIER
cChIP-seq	This study	GEO: GSE119618
cnRNA-seq	This study	GEO: GSE119619
Experimental Models: Cell Lines		
Mouse ESC: *Pcgf1*$^{fl/fl}$	Almeida et al., 2017	N/A
Mouse ESC: *Pcgf3*$^{fl/fl}$	Almeida et al., 2017	N/A
Mouse ESC: *Pcgf5*$^{fl/fl}$	This study	N/A
Mouse ESC: *Pcgf6*$^{fl/fl}$	Endoh et al., 2017	N/A
Mouse ESC: *Pcgf3/5*$^{fl/fl}$	This study	N/A
Mouse ESC: *Pcgf1/3/5*$^{fl/fl}$	This study	N/A
Mouse ESC: *Pcgf1/3/5/2*$^{fl/fl}$	This study	N/A
Mouse ESC: *Pcgf1/3/5/6*$^{fl/fl}$	This study	N/A
Mouse ESC: *Ring1a*$^{-/-}$;*Ring1b*$^{fl/fl}$	This study	N/A
Mouse ESC: *Pcgf4*$^{-/-}$;*Pcgf2*$^{fl/fl}$	This study	N/A
Mouse ESC: *Pcgf4*$^{-/-}$;*Pcgf1/3/5/2*$^{fl/fl}$	This study	N/A
Mouse ESC: C57BL/6JJcl x 129/SvJcl with *Xist* WT transgene (P4D7-F4)	Pintacuda et al., 2017	N/A
Oligonucleotides		
Pcgf2 loxP site #1 sgRNA target, AAAGACTGAAGTCAGACCAT	This study	N/A
Pcgf2 loxP site #2 sgRNA target, TGAGTGACCCCTGAATCCAC	This study	N/A
Pcgf4 deletion sgRNA #1 target, TTTAACAGTGGAGGTTATTC	This study	N/A
Pcgf4 deletion sgRNA #2 target, AATGTTAGTTCATAACTGTG	This study	N/A
Pcgf6 loxP site #1 sgRNA target, AGTTAGATGTCTAAGGCTAC	This study	N/A
Pcgf6 loxP site #2 sgRNA target, ATCTGAGGTTAAAGCCAGCA	This study	N/A
Ring1a deletion sgRNA #1 target, CTCAGCGGAGCCCCGCTTGG	This study	N/A
Ring1a deletion sgRNA #2 target, GCGACCGTGCAGCTGACGTT	This study	N/A
Ring1b loxP site #1 sgRNA target, GATCAGTGAAGCTAGCGATG	This study	N/A
Ring1b loxP site #2 sgRNA target, TTGCAGGCTAAGACCACATT	This study	N/A
Rosa26 locus sgRNA target, CGCCCATCTTCTAGAAAGAC	This study	N/A
Software and Algorithms		
SAMtools (v1.7)	Li et al., 2009	http://www.htslib.org/
BEDtools (v2.17.0)	Quinlan, 2014	https://bedtools.readthedocs.io/en/latest/
Bowtie 2 (v2.3.4)	Langmead and Salzberg, 2012	http://bowtie-bio.sourceforge.net/bowtie2/index.shtml
SNPsplit (v0.3.3)	Krueger and Andrews, 2016	http://www.bioinformatics.babraham.ac.uk/projects/SNPsplit/
Sambamba (v0.6.7)	Tarasov et al., 2015	http://lomereiter.github.io/sambamba/
STAR (v2.5.4)	Dobin et al., 2013	https://github.com/alexdobin/STAR
deepTools (v3.0.1)	Ramírez et al., 2014	https://deeptools.readthedocs.io/en/develop/
MACS2 (v2.1.1)	Zhang et al., 2008	https://github.com/taoliu/MACS/tree/master/MACS2
UCSC Genome Browser	Kent et al., 2002	https://genome.ucsc.edu/
Bioconductor (v3.6)	Huber et al., 2015	https://www.bioconductor.org/
DESeq2	Love et al., 2014	https://bioconductor.org/packages/release/bioc/html/DESeq2.html
UpSetR	Lex et al., 2014	https://cran.r-project.org/web/packages/UpSetR/README.html
HOMER	Heinz et al., 2010	http://homer.ucsd.edu/homer/

CONTACT FOR REAGENT AND RESOURCE SHARING

Further information and requests for resources and reagents should be directed to and will be fulfilled by the Lead Contact, Rob Klose (rob.klose@bioch.ox.ac.uk).

EXPERIMENTAL MODEL AND SUBJECT DETAILS

Unless otherwise indicated, male mouse embryonic stem cells were grown on gelatin-coated plates at 37°C and 5% CO2, in Dulbecco's Modified Eagle Medium (DMEM) supplemented with 15% fetal bovine serum (Labtech), 2 mM L-glutamine (Life Technologies), 1x penicillin-streptomycin solution (Life Technologies), 1x non-essential amino acids (Life Technologies), 0.5 mM beta-mercaptoethanol (Life Technologies), and 10 ng/mL leukemia inhibitory factor. To induce conditional removal of PRC1 complexes individually or in combination, cells were treated with 800 nM 4-hydroxytamoxifen (OHT) for 72 hours. Cells were regularly tested for the presence of mycoplasma.

Pcgf6$^{fl/fl}$ ESCs and *Mus domesticus* (129S1) x *Mus castaneus* F1 hybrid ESCs were grown on a monolayer of mitomycin-inactivated SNLP feeders (STO mouse fibroblasts expressing Neomycin, Puromycin resistance and *Lif* genes) in otherwise the same conditions as other mouse ESCs. Prior to harvesting these ES cells, the feeders were depleted by pre-plating trypsinised cells for 30 mins at 37°C on plates not coated with gelatin and discarding attached cells. To induce expression of the *Xist* transgene driven by a TetO promoter, *Mus domesticus* (129S1) x *Mus castaneus* F1 hybrid ESCs with a full length *Xist* transgene integrated into the *Mus castaneus* allele of chromosome 3 (clone P4D7F4) (Pintacuda et al., 2017) were treated with 1.5 μg/mL doxycycline for 72 hours.

Human HEK293T cells used for calibrated ChIP-seq were grown at 37°C and 5% CO2, in Dulbecco's Modified Eagle Medium (DMEM) supplemented with 10% fetal bovine serum (Labtech), 2 mM L-glutamine (Life Technologies), 1x penicillin-streptomycin solution (Life Technologies), and 0.5 mM beta-mercaptoethanol (Life Technologies).

Drosophila S2 (SG4) cells were grown adhesively at 25°C in Schneider's *Drosophila* Medium (Life Technologies), supplemented with 1x penicillin-streptomycin solution (Life Technologies) and 10% heat-inactivated fetal bovine serum (Labtech).

METHOD DETAILS

Genome engineering by CRISPR/Homology-Directed Repair (HDR)

The pSptCas9(BB)-2A-Puro(PX459)-V2.0 vector was obtained from Addgene (#62988) and sgRNAs were designed using the CRISPOR online tool (http://crispor.tefor.net/crispor.py). Targeting constructs with appropriate homology arms were generated by Gibson assembly using the Gibson Assembly Master Mix kit (New England Biolabs), or in the case of single LoxP sites with 150 bp homology arms, purchased from GeneArt Gene Synthesis (Invitrogen). In all instances, targeting constructs were designed such that Cas9 recognition sites were disrupted by the presence of the LoxP site. ESCs (one well of a 6-well plate) were transfected with 0.5 μg of each Cas9 guide, and 1 μg of targeting construct (where appropriate) using Lipofectamine 3000 (ThermoFisher) according to manufacturer's guidelines. The day after transfection, cells were passaged at a range of densities and subjected to puromycin selection (1 μg/ml) for 48 hours to eliminate any non-transfected cells. Approximately one week later, individual clones were isolated, expanded, and PCR-screened for the desired genomic modification.

Cell line generation

Mouse ESCs were targeted to produce appropriate conditional alleles for *Pcgf1*, *Pcgf3* (Almeida et al., 2017), *Pcgf5,* and *Pcgf6* (Endoh et al., 2017). For each gene, parallel LoxP sites were inserted such that they flanked a critical portion of the gene: for *Pcgf1* the LoxP sites flanked exons 2-7, for *Pcgf3* exon 4 was flanked (containing ATG start codon), for *Pcgf5* exon 2 (containing ATG start codon) was flanked, and for *Pcgf6* exons 2-3 were flanked. *Pcgf1$^{fl/fl}$*, *Pcgf3$^{fl/fl}$* and *Pcgf5$^{fl/fl}$* mouse lines were established by injecting targeted ESCs into 8-cell stage embryos to create chimeric mouse lines. *Pcgf1$^{fl/fl}$*, *Pcgf3$^{fl/fl}$* and *Pcgf5$^{fl/fl}$* mouse lines were crossed with *Rosa26*::*CreERT2* (ERT2-Cre) transgenic mice purchased from Artemis Pharmaceuticals, and further intercrossed to generate *Pcgf3/5$^{fl/fl}$*, and *Pcgf1/3/5$^{fl/fl}$* mice. *Pcgf1$^{fl/fl}$*, *Pcgf3$^{fl/fl}$*, *Pcgf5$^{fl/fl}$*, *Pcgf3/5$^{fl/fl}$*, and *Pcgf1/3/5$^{fl/fl}$* ESCs (all *Rosa26*::*CreERT2*) were derived from blastocysts as described previously (Endoh et al., 2008). *Pcgf6$^{fl/fl}$* cells were generated as described previously (Endoh et al., 2017). ESCs were initially maintained on a monolayer of mitomycin-inactivated fibroblast feeders, before being adapted to grow under feeder-free conditions (plates were coated with gelatin only). All animal experiments were carried out according to the in-house guidelines for the care and use of laboratory animals of the RIKEN Center for Integrative Medical Sciences, Yokohama, Japan.

Pcgf4$^{-/-}$;Pcgf2$^{fl/fl}$ ESCs were derived from *Rosa26::CreERT2* ESCs by a two-step process. First, exon 2 of both *Pcgf4* alleles was deleted using two Cas9 guides flanking the deletion region. Genomic DNA samples from ESC clones were screened for the desired deletion by PCR, and correct clones were validated by sequencing of the PCR products. A validated *Pcgf4$^{-/-}$* ESC clone was subjected to a second round of genomic engineering to flank the first coding exon of *Pcgf2* (exon 2) with LoxP sites in a parallel orientation using homology arms of approximately 1 kb and appropriate Cas9 guides. *Pcgf4$^{-/-}$;Pcgf2$^{fl/fl}$* clones were analyzed by both RT-qPCR and western blot to confirm PCGF2 removal (at both the RNA and protein level) in response to OHT treatment.

Both $Pcgf1/3/5/2^{fl/fl}$ and $Pcgf1/3/5/6^{fl/fl}$ ESCs were derived from $Pcgf1/3/5^{fl/fl}$ ESCs by a two-step process in which upstream and downstream LoxP sites were sequentially inserted using targeting constructs with 150 bp arms of homology and appropriate Cas9 guides. For *Pcgf2*, the LoxP sites flanked the first coding exon (exon 2), while for *Pcgf6* exons 2-3 were flanked. Appropriately targeted clones were subjected to OHT treatment and analyzed by RT-qPCR and western blot to validate PCGF2 or PCGF6 removal. $Pcgf4^{-/-};Pcgf1/3/5/2^{fl/fl}$ ESCs were derived from $Pcgf1/3/5/2^{fl/fl}$ ESCs by deleting exon 2 of both *Pcgf4* alleles using two Cas9 guides flanking the deletion region (as described above for $Pcgf4^{-/-};Pcgf2^{fl/fl}$ ESCs).

$Ring1a^{-/-};Ring1b^{fl/fl}$ ESCs used in this study were derived from E14 mouse ESCs. Exons 1-3 of Ring1a were first deleted using two Cas9 guides flanking the deletion region, and in parallel CreERT2 was inserted into the Rosa26 locus using a Rosa26-specific Cas9 guide. $Ring1a^{-/-};CreERT2$ ESCs were then subjected to two rounds of genome editing to sequentially insert LoxP sites flanking the first coding exon of *Ring1b*. $Ring1a^{-/-};Ring1b^{fl/fl}$ ESCs were validated by RT-qPCR and western blot to confirm constitutive deletion of RING1A and conditional RING1B removal in response to OHT treatment.

Calibrated ChIP-sequencing (cChIP-seq)

For RING1B, SUZ12, PCGF1, PCGF2, CBX7 and PHC1 cChIP-seq, 5 × 10^7 mouse ESCs (both untreated and following 72 hours OHT treatment) were mixed with 2 × 10^6 human HEK293T cells. Cells were resuspended in 10 mL phosphate buffered saline (PBS) and crosslinked at 25°C first with 2 mM DSG (Thermo Scientific) for 45 mins, and then with 1% formaldehyde (methanol-free, Thermo Scientific) for a further 15 minutes. Reactions were quenched by the addition of 125 mM glycine. Crosslinked cells were incubated in lysis buffer (50 mM HEPES pH 7.9, 140 mM NaCl, 1 mM EDTA, 10% glycerol, 0.5% NP40, 0.25% Triton X-100) for 10 min at 4°C. The released nuclei were then washed (10 mM Tris-HCl pH 8, 200 mM NaCl, 1 mM EDTA, 0.5 mM EGTA) for 5 min at 4°C. Chromatin was then resuspended in 1 mL of sonication buffer (10 mM Tris-HCl pH 8, 100 mM NaCl, 1 mM EDTA, 0.5 mM EGTA, 0.1% Na deoxycholate, 0.5% N-lauroylsarcosine) and sonicated for 30 min using a BioRuptor Pico sonicator (Diagenode), shearing genomic DNA to an average size of approximately 0.5 kb. Following sonication, Triton X-100 was added to a final concentration of 1%.

For ChIP, sonicated chromatin was diluted 10-fold in ChIP dilution buffer (1% Triton X-100, 1 mM EDTA, 20 mM Tris-HCl pH 8, 150 mM NaCl) and pre-cleared for 1 hour using Protein A agarose beads (Repligen) blocked with 1 mg/ml BSA and 1 mg/ml yeast tRNA. For each ChIP reaction, 1ml of diluted and pre-cleared chromatin was incubated overnight with the appropriate antibody, anti-RING1B (CST, D22F2, 3 ul), anti-SUZ12 (CST, D39F6, 3 ul), anti-PCGF1 (in-house, 3 ul), anti-PCGF2 (Santa Cruz, sc-10744, 3 ul), anti-CBX7 (Abcam, ab21873, 4 ul), anti-PHC1 (CST, 1F3F3, 6 ul). Antibody-bound chromatin was captured using blocked protein A agarose for 2 hours at 4°C and collected by centrifugation. ChIP washes were performed as described previously (Farcas et al., 2012). ChIP DNA was eluted in elution buffer (1% SDS, 0.1 M NaHCO3) and cross-links were reversed overnight at 65°C with 200 mM NaCl and 2 µL of RNase A (Sigma). A matched input sample (corresponding to 10% of original ChIP reaction) was identically treated. The following day, ChIP samples and Inputs were incubated with Proteinase K (Sigma) for 1.5 hours at 56°C and purified using ChIP DNA Clean and Concentrator Kit (Zymo Research).

cChIP-seq libraries for both ChIP and Input samples were prepared using NEBNext Ultra DNA Library Prep Kit for Illumina, following manufacturer's guidelines. Samples were indexed using NEBNext Multiplex Oligos. The average size and concentration of all libraries was analyzed using the 2100 Bioanalyzer High Sensitivity DNA Kit (Agilent) followed by qPCR using SensiMix SYBR (Bioline, UK) and KAPA Illumina DNA standards (Roche). Libraries were sequenced as 40 bp paired-end reads on Illumina NextSeq 500 platform in biological triplicate unless otherwise specified.

Native cChIP-sequencing

For H2AK119ub1 and H3K27me3 native cChIP-seq, 5 × 10^7 mouse ESCs (both untreated and following 72 hours OHT treatment) were mixed with 2 × 10^7 *Drosophila* SG4 cells in PBS. Mixed cells were pelleted and nuclei were released by resuspending in ice cold lysis buffer (10mM Tris-HCl pH 8.0, 10 mM NaCl, 3 mM $MgCl_2$, 0.1% NP40, 5 mM N-ethylmaleimide). Nuclei were then washed, and resuspended in 1 mL of MNase digestion buffer (10 mM Tris-HCl pH 8.0, 10 mM NaCl, 3 mM $MgCl_2$, 0.1% NP40, 0.25M sucrose, 3mM $CaCl_2$, 10 mM N-ethylmaleimide, 1x protease inhibitor cocktail (Roche)). Each sample was incubated with 200 units of MNase (Fermentas) at 37°C for 5 min, followed by the addition of 4 mM EDTA to halt MNase digestion. Following centrifugation at 1500 g for 5 min at 4°C, the supernatant (S1) was retained. The remaining pellet was incubated with 300 µl of nucleosome release buffer (10 mM Tris-HCl pH 7.5, 10 mM NaCl, 0.2 mM EDTA, 1x protease inhibitor cocktail (Roche), 10 mM N-ethylmaleimide) at 4°C for 1 h, passed five times through a 27G needle using a 1 mL syringe, and spun at 1500 g for 5 min at 4°C. The second supernatant (S2) was collected and combined with corresponding S1 sample from above. A small amount of S1/S2 DNA was purified and visualized on a 1.5% agarose gel to confirm digestion to mostly mono-nucleosomes.

For ChIP experiments, S1/S2 nucleosomes were diluted 10-fold in native ChIP incubation buffer (70 mM NaCl, 10 mM Tris pH 7.5, 2 mM MgCl2, 2 mM EDTA, 0.1% Triton, 1x protease inhibitor cocktail (Roche), 10 mM N-ethylmaleimide (NEM)), and 1 mL aliquots were made. Each ChIP reaction was then incubated overnight at 4°C with 5 µL of anti-H2AK119ub1 (Cell Signaling Technology, D27C4) or 5 µL of anti-H3K27me3 (in-house) antibody. Antibody-bound nucleosomes were captured using protein A agarose (Repligen) beads, pre-blocked in native ChIP incubation buffer supplemented with 1 mg/ml BSA and 1 mg/ml yeast tRNA, for 1 hour at 4°C and collected by centrifugation. Immunoprecipitated material was washed four times with Native ChIP wash buffer (20 mM Tris pH 7.5, 2 mM EDTA, 125 mM NaCl, 0.1% Triton X-100) and once with Tris-EDTA buffer (10 mM Tris pH 8, 1 mM EDTA). ChIP DNA was eluted using 100 µL of elution buffer (1% SDS, 0.1 M NaHCO3), and then purified using ChIP DNA Clean

and Concentrator Kit (Zymo Research). For each individual ChIP sample, DNA from a matched Input control (corresponding to 10% of original ChIP reaction) was also purified.

Native cChIP-seq library preparation and sequencing was performed as described above for cChIP-seq. For allele-specific analysis of H2AK119ub1 ChIP-seq in the *Mus domesticus* (129S1) x *Mus castaneus* F1 hybrid ESC line with inducible *Xist* transgene, native cChIP-seq libraries were sequenced as 80 bp paired-end reads on Illumina NextSeq 500 platform to increase the number of reads overlapping allele-specific SNPs.

Calibrated nuclear RNA-sequencing (cnRNA-seq)

For preparation of RNA for cnRNA-seq, 1 × 10^7 mouse ESCs (both untreated and following 72 hours OHT treatment) were mixed with 4 × 10^6 *Drosophila* SG4 cells in PBS. Nuclei were isolated in 1 mL HS Lysis buffer (50 mM KCl, 10 mM $MgSO_4.7H_20$, 5 mM HEPES, 0.05% NP40 (IGEPAL CA630)), 1 mM PMSF, 3 mM DTT) for 1 min at room temperature. They were then recovered by centrifugation at 1000 × *g* for 5 min at 4°C, followed by a total of three washes with ice-cold RSB buffer (10 mM NaCl, 10 mM Tris (pH 8.0), 3 mM $MgCl_2$). Nuclei integrity was assessed using 0.4% Trypan Blue staining (ThermoScientific). Next, nuclei were resuspended in 1 mL of TRIzol reagent (ThermoScientific) and RNA was extracted according to the manufacturer's protocol, followed by treatment with the TURBO DNA-free Kit (ThermoScientific). Quality of RNA was assessed using 2100 Bioanalyzer RNA 6000 Pico kit (Agilent). Next, RNA samples were depleted of rRNA using the NEBNext rRNA Depletion kit (NEB). RNA-seq libraries were prepared using the NEBNext Ultra Directional RNA Library Prep kit (NEB) and libraries were sequenced as 80 bp paired-end reads on the Illumina NextSeq 500 platform in biological triplicate.

To quantitate the consistency of spike-in cell mixing for each individual sample, a small aliquot of nuclei was used to isolate genomic DNA using phenol-chloroform extraction. This was followed by sonication of DNA for 13-15 min using a BioRuptor Pico sonicator (Diagenode), shearing genomic DNA to an average size of less than 1 kb. Libraries from sonicated genomic DNA were constructed as described above for cChIP-seq and sequenced as 80 bp paired-end reads on the Illumina NextSeq 500 platform.

Preparation of nuclear and histone extracts and immunoblotting

For nuclear extraction, ESCs were washed with PBS and then resuspended in 10 volumes of Buffer A (10 mM HEPES pH 7.9, 1.5 mM MgCl2, 10 mM KCl, 0.5 mM DTT, 0.5 mM PMSF and protease inhibitor cocktail (Roche)). After 10 min incubation on ice, cells were recovered by centrifugation at 1500 g for 5 min and resuspended in 3 volumes of Buffer A supplemented with 0.1% NP-40. The released nuclei were pelleted by centrifugation at 1500 g for 5 min, followed by resuspension in 1 volume of Buffer C (5 mM HEPES (pH 7.9), 26% glycerol, 1.5 mM MgCl 2, 0.2 mM EDTA, protease inhibitor cocktail (Roche) and 0.5mM DTT) supplemented with 400 mM NaCl. The extraction was allowed to proceed on ice for 1 hour with occasional agitation, then the nuclei were pelleted by centrifugation at 16,000 g for 20 min at 4°C. The supernatant was taken as the nuclear extract.

For histone extraction, ESCs were washed with RSB supplemented with 20 mM NEM, incubated on ice for 10 min in RSB with 0.5% NP-40 and 20 mM NEM, pelleted by centrifugation at 800 g for 5 min and incubated in 2.5 mM MgCl2, 0.4 M HCl and 20 mM NEM on ice for 30 min. After that, cells were pelleted by centrifugation at 16,000 g at 4°C for 20 min, the supernatant recovered and precipitated on ice with 25% TCA for 30 min, followed by centrifugation at 16,000 g for 15 min at 4°C to recover histones. Following two acetone washes, the histones were resuspended in 150 μL 1xSDS loading buffer and boiled at 95°C for 5 min. Finally, any insoluble precipitate was pelleted by centrifugation at 16,000 g for 15 min at 4°C and the soluble fraction retained as the histone extract. Histone concentrations across samples were compared by Coomassie Blue staining following SDS-PAGE. Semiquantitative western blot analysis of histone extracts was performed using LI-COR IRDye® secondary antibodies and imaging was done using the LI-COR Odyssey Fc system. To measure the changes in bulk H2AK119ub1 levels, the relative signal of H2AK119ub1 to H3 or H4 histones was quantified.

Co-immunoprecipitation

For co-immunoprecipitation reactions, 400 μg of nuclear extract from *Pcgf4*$^{-/-}$;*Pcgf2*$^{fl/fl}$ ESCs (before and after OHT treatment) was added to BC150 buffer (150 mM KCl, 10% glycerol, 50 mM HEPES (pH 7.9), 0.5 mM EDTA, 0.5 mM DTT) with 1x protease inhibitor cocktail (Roche) to a total volume of 550 μl. A 50 μL Input sample was retained, and 5 μg of mouse monoclonal anti-RING1B antibody (Atsuta et al., 2001) was added to the remaining 500 μL of sample. Immunoprecipitation reactions were then incubated overnight at 4°C. Immunoprecipitated material was collected with Protein A agarose beads and washed four times in 1 mL of BC150 buffer. Following the final wash step, beads were directly resuspended in 100 μL of 1x SDS loading buffer (2% SDS, 0.1 M Tris pH 6.8, 0.1 M DTT, 10% glycerol, 0.1% bromophenol blue) and placed at 95°C for 5 mins. 1x SDS loading buffer was similarly added to Input samples which were also incubated at 95°C for 5 mins, prior to SDS-PAGE and western blot analysis.

QUANTIFICATION AND STATISTICAL ANALYSIS

Massively parallel sequencing, *data processing and normalization*

For calibrated ChIP-seq, paired-end reads were aligned to the genome sequence of concatenated mouse and spike-in genomes (mm10+dm6 for native cChIP-seq and mm10+hg19 for cross-linked cChIP-seq) using Bowtie 2 (Langmead and Salzberg, 2012) with the "–no-mixed" and "–no-discordant" options specified. Reads that were mapped more than once were discarded, followed

by removal of PCR duplicates with SAMTools (Li et al., 2009) for native cChIP-seq or Sambamba for cross-linked cChIP-seq (Tarasov et al., 2015).

For cnRNA-seq, first, paired-end reads were aligned using Bowtie 2 (with "–very-fast," "–no-mixed" and "–no-discordant" options) against the concatenated mm10 and dm6 rRNA genomic sequence (GenBank: BK000964.3 and M21017.1), to filter out reads mapping to rDNA fragments. All unmapped reads from the first step, were aligned against the genome sequence of concatenated mm10 and dm6 genomes using STAR (Dobin et al., 2013). To improve mapping of intronic sequences of nascent transcripts abundant in nuclear RNA-seq, reads which failed to map using STAR were aligned against the mm10+dm6 concatenated genome using Bowtie 2 (with "–sensitive-local," "–no-mixed" and "–no-discordant" options). PCR duplicates were removed using SAMTools. A list of all cChIP-seq and cnRNA-seq experiments performed in this study and the number of uniquely aligned reads for both mouse and spike-in genomes is provided in Table S1.

To internally calibrate our cChIP-seq and cnRNA-seq experiments, we spiked-in a fixed number of control cells (*Drosophila* SG4 cells for native cChIP-seq and cnRNA-seq and human HEK293T cells for cross-linked cChIP-seq) to each experimental sample. This exogenous genome spike-in was then used to quantitatively compare the genomic profiles of chromatin modifications or gene expression between experimental conditions as described previously (Bonhoure et al., 2014; Hu et al., 2015; Orlando et al., 2014). Briefly, for annotation of genomic intervals and data visualization, mm10 reads were randomly subsampled using factors that reflected the total number of dm6 (or hg19) reads in each sample. To account for any minor variation in spike-in cell mixing in different biological replicates, the downsampling factors were additionally corrected using the ratio of dm6 (or hg19)/mm10 total read counts in corresponding Input samples. For cnRNA-seq, corresponding genomic DNA-seq samples were used to calculate the ratio of dm6/mm10 total read counts. For additional clarity, we provide the formula used for calculation of downsampling factors:

$$Downsampling\ factor = \alpha \times \frac{1}{N(spikeIn\ in\ ChIP\ or\ RNA)} \times \frac{N(spikeIn\ in\ Input)}{N(mouse\ in\ Input)}$$

where *N (spikeIn in ChIP or RNA)* - total number of reads aligning to the spike-in genome for each ChIP-seq/RNA-seq sample; *N (spikeIn in Input)* - total number of reads aligning to the spike-in genome in the corresponding Input sample; *N (mouse in Input)* - total number of reads aligning to the mouse genome in the corresponding Input sample; α - coefficient applied for all the files normalized together so the value of the largest downsampling factor equals 1.

Allele-specific analysis of H2AK119ub1 cChIP-seq in the *Mus domesticus* (129S1) *x Mus castaneus* F1 hybrid ESC line with inducible full-length *Xist* transgene was performed as described previously with minor alterations (Pintacuda et al., 2017). Briefly, paired-end reads were aligned using STAR (with "–outFilterMultimapNmax 1," "–outFilterMismatchNmax 2," "–alignEndsType EndToEnd" parameters) against the concatenated mm10+dm6 genome, in which polymorphic SNPs for 129S1 and CAST mouse strains were N-masked. Reads that mapped more than once were discarded and PCR duplicates were removed with Sambamba. Reads mapping to mm10 genome were randomly subsampled using downsampling factors calculated based on spike-in calibration as described previously. To separate reads specifically mapping to the 129S1 and CAST alleles, we used SNPsplit (Krueger and Andrews, 2016) with the paired-end mode.

To compare replicates, read coverage across regions of interest (RING1B peaks for cChIP-seq or gene bodies for cnRNA-seq) was analyzed using multiBamSummary and plotCorrelation functions from deepTools suite (Ramírez et al., 2014). For each condition, biological replicates correlated well with each other (Pearson correlation coefficient > 0.9, see Tables S2 and S3) and were merged for downstream applications. Genome coverage tracks were generated using the pileup function from MACS2 (Zhang et al., 2008) for cChIP-seq and genomeCoverageBed from BEDTools (Quinlan, 2014) for cnRNA-seq and visualized using the UCSC genome browser (Kent et al., 2002).

Peak calling

To identify PRC1- or PRC2-bound regions, we used cChIP-seq data for RING1B and SUZ12 respectively that were obtained from *Pcgf4*$^{-/-}$;*Pcgf2*$^{fl/fl}$ and *Pcgf1/3/5*$^{fl/fl}$ ESCs. RING1B and SUZ12 peak sets were generated with the MACS2 function (–broad option specified) using corresponding Input samples for background normalization. For each factor, a set of peaks identified in all biological replicates and in both cell lines was used for further analysis. Peaks overlapping with a custom-build blacklist were discarded to remove sequencing artifacts. For RING1B peaks, RING1B cChIP-seq data from *Ring1a*$^{-/-}$; *Ring1b*$^{fl/fl}$ ESCs following OHT treatment was used to filter out peaks which retained RING1B signal following loss of RING1B. In total, 8833 RING1B peaks and 5965 SUZ12 peaks were identified. Classical Polycomb chromatin domains were identified by k-means clustering of ± 10 kb windows around the center of RING1B peaks which was based on RING1B and SUZ12 cChIP-seq signal in untreated *Pcgf4*$^{-/-}$;*Pcgf2*$^{fl/fl}$ ESCs using deeptools (v.3.0.1). This generated three clusters, two of which displayed high enrichment of both RING1B and SUZ12 and were combined into one set of genomic intervals (n = 2096). To characterize pervasive genomic deposition of H2AK119ub1, we have generated a set of 100 kb windows spanning the genome (n = 27,348) using makewindows function from BEDtools (v2.17.0).

Read count quantitation and analysis

For cChIP-seq, metaplot and heatmap analysis of read density at the PRC1-bound sites and classical Polycomb chromatin domains was performed using computeMatrix and plotProfile/plotHeatmap functions from deeptools (v.3.0.1). For these analyses, read density at the center of genomic intervals in untreated cells was set to 1 for each cChIP-seq dataset and each cell line individually. For

chromosome-wide density plots and heatmaps, read coverage in 250 kb bins was calculated with a custom R (v3.4.4) script utilizing packages (*GenomicRanges*, *GenomicAlignments* and *Rsamtools*) that are distributed by Bioconductor (Huber et al., 2015) and visualized using ggplot2. For cChIP-seq, intervals of interest were annotated with spike-in normalized read counts from merged replicates using a custom Perl script utilizing SAMtools (v1.7). For differential enrichment analysis, read counts in the intervals of interest were obtained for individual replicates from original bam files prior to spike-in normalization using a custom-made Perl script. For differential gene expression analysis, the same read count quantitation approach was applied for a custom-built, non-redundant mm10 gene set. Briefly, mm10 refGene genes were filtered to remove very short genes with poor sequence mappability and highly similar transcripts which resulted in the final set of 20,633 genes.

The distribution of log2-fold changes and normalized read count values for different genomic intervals was visualized using custom R scripts and ggplot2. For boxplot analysis comparing H2AK119ub1 and RING1B enrichment at PRC1-bound sites and 100 kb genomic windows, read counts were normalized to the genomic region size (in kb) and to the median value of cChIP-seq signal at PRC1-bound sites in untreated cells which was then set to 1. For boxplot analyses comparing normalized cChIP-seq signal across different groups of genomic intervals before and after treatment, log2 read counts were normalized to the median value in untreated cells which was then set to 1 for each cell line individually. Boxes for the boxplots and the center of each violin plot show interquartile range (IQR) and whiskers extend by 1.5xIQR. Correlation analyses were also performed in R using Pearson correlation and visualized with scatterplots colored by density using stat_density2d.

Differential cChIP-seq enrichment and gene expression analysis

To identify significant changes in chromatin binding/enrichment or gene expression, a custom-made R script utilizing DESeq2 package was used (Love et al., 2014). To incorporate spike-in calibration into this analysis, read counts for the spike-in genome at a control set of intervals were supplied to calculate DESeq2 size factors which were then used for DESeq2 normalization of raw mm10 read counts (similarly to the approach described in (Taruttis et al., 2017)). A set of unique dm6 refGene genes was used for spike-in normalization of cnRNA-seq and native cChIP-seq, while a set of hg19 CpG island regions was obtained from UCSC Table Browser for RING1B and SUZ12 cChIP-seq differential enrichment analysis. Prior to quantitation, spike-in reads were pre-normalized to accurately reflect the actual spike-in ratio derived from a corresponding Input or genomic DNA-seq sample. For a change to be called significant, we applied a threshold of p-adj < 0.05 and fold change > 1.5 for cnRNA-seq and p-adj < 0.05 for cChIP-seq. Log2-fold change values were visualized using R and ggplot2 with MA plots and violin plots. For MA plots, density of the data points across y axis is shown to reflect the general direction of gene expression changes.

Gene annotation

Mouse genes from a custom-build non-redundant set (n = 20,633) were classified into three categories based on the presence of a non-methylated CpG island (NMI) and PRC1+PRC2 binding at their promoters. We defined PcG-occupied genes as genes for which corresponding TSS regions (±2500 bp) overlap with stringent RING1B and SUZ12 peak sets identified in this study. These genes (n = 4868) represent bona fide Polycomb target genes. Furthermore, genes that did not overlap with genomic regions bound by both PRC1 and PRC2 but contain a non-methylated CpG island (NMI) were classified as non-PcG-occupied genes (n = 9898). Finally, genes that did not overlap with a non-methylated CpG island (NMI) are referred to as non-NMI genes (n = 5867). To identify non-methylated CpG islands in the mouse genome, we used an experimental definition derived from BioCAP-seq data using MACS2 peak calling (Long et al., 2013). To compare gene expression changes across different cell lines to those observed in RING1A/B-deficient cells, we also isolated PRC1 target genes (n = 2071) which were defined as a subset of PcG-occupied genes which showed a statistically significant increase in gene expression following removal of PRC1 in *Ring1a*$^{-/-}$;*Ring1b*$^{fl/fl}$ ESCs. For comparison of individual PCGF occupancy at promoters of variant PRC1-regulated genes, PCGF1-sensitive genes were defined as genes significantly upregulated following OHT treatment in *Pcgf1*$^{fl/fl}$ ESCs. PCGF1/3/5-sensitive genes were defined as the set of genes significantly upregulated following OHT treatment in *Pcgf1/3/5*$^{fl/fl}$ ESCs, excluding the PCGF1-sensitive set of genes. Similarly, PCGF1/3/5/6-sensitive genes were defined as the set of genes significantly upregulated following OHT treatment in *Pcgf1/3/5/6*$^{fl/fl}$ ESCs, excluding the PCGF1- and PCGF1/3/5-sensitive sets of genes.

To identify genes that were further reactivated following removal of PCGF2 in combination with PCGF1/3/5 in *Pcgf1/3/5/2*$^{fl/fl}$ ESCs, we have performed a differential analysis comparing expression of genes in OHT-treated *Pcgf1/3/5*$^{fl/fl}$ and *Pcgf1/3/5/2*$^{fl/fl}$ ESCs. This was done using a traditional DESeq2 approach. PRC1-bound sites that retained RING1B following removal of PCGF1/3/5/6 were identified as sites with no significant decrease in RING1B signal by cChIP-seq (sites with p-adj < 0.05 and fold change < 0 were excluded). We then limited our analysis to the sites that overlapped with the promoters (TSS ± 2500 bp) of PRC1 target genes characterized earlier. To identify genes that depend on PCGF6 for normal level of H2AK119ub1 at their promoters, we have overlapped PRC1-bound sites that displayed a significant decrease in H2AK119ub1 following OHT treatment in *Pcgf6*$^{fl/fl}$ ESCs with promoters of genes from a custom non-redundant mm10 set. HOMER was used to perform gene ontology (GO) enrichment analysis for genes that showed significant reductions in promoter-associated H2AK119ub1 following PCGF6 removal and genes that retained RING1B at their promoters following removal of PCGF1/3/5/6. For gene expression analysis in *Pcgf6*$^{fl/fl}$ ESCs before and after 8 days of OHT treatment, we used FPKM values obtained with cufflinks for refGene genes as reported previously (Endoh et al., 2017). UpSet plots were generated using the UpSetR package available on CRAN (Lex et al., 2014).

DATA AND SOFTWARE AVAILABILITY

The high-throughput data reported in this study have been deposited in GEO under the accession number GSE119620. Published data used in this study include BioCAP-seq (GSE43512 (Long et al., 2013)); RNA-seq gene expression data from *Pcgf6*$^{fl/fl}$ ESCs (GSE84480 (Endoh et al., 2017)); 4sU RNA-seq gene expression data from the *Mus domesticus* (129S1) *x Mus castaneus* F1 hybrid ESC line with randomly integrated full-length *Xist* transgene (GSE103370 (Pintacuda et al., 2017)); 4sU RNA-seq gene expression data for mESCs following RA-induced differentiation (GSE98756 (Dimitrova et al., 2018)); KDM2B ChIP-seq (GSE55698 (Blackledge et al., 2014)); PCGF5 ChIP-seq (GSE107377 (Yao et al., 2018)); PCGF6, MGA, and L3MBTL2 ChIP-seq (ArrayExpress E-MTAB-6007 (Stielow et al., 2018). All R and Perl scripts used for data analysis in this study are available upon request.

Molecular Cell, Volume *74*

Supplemental Information

Synergy between Variant PRC1 Complexes Defines Polycomb-Mediated Gene Repression

Nadezda A. Fursova, Neil P. Blackledge, Manabu Nakayama, Shinsuke Ito, Yoko Koseki, Anca M. Farcas, Hamish W. King, Haruhiko Koseki, and Robert J. Klose

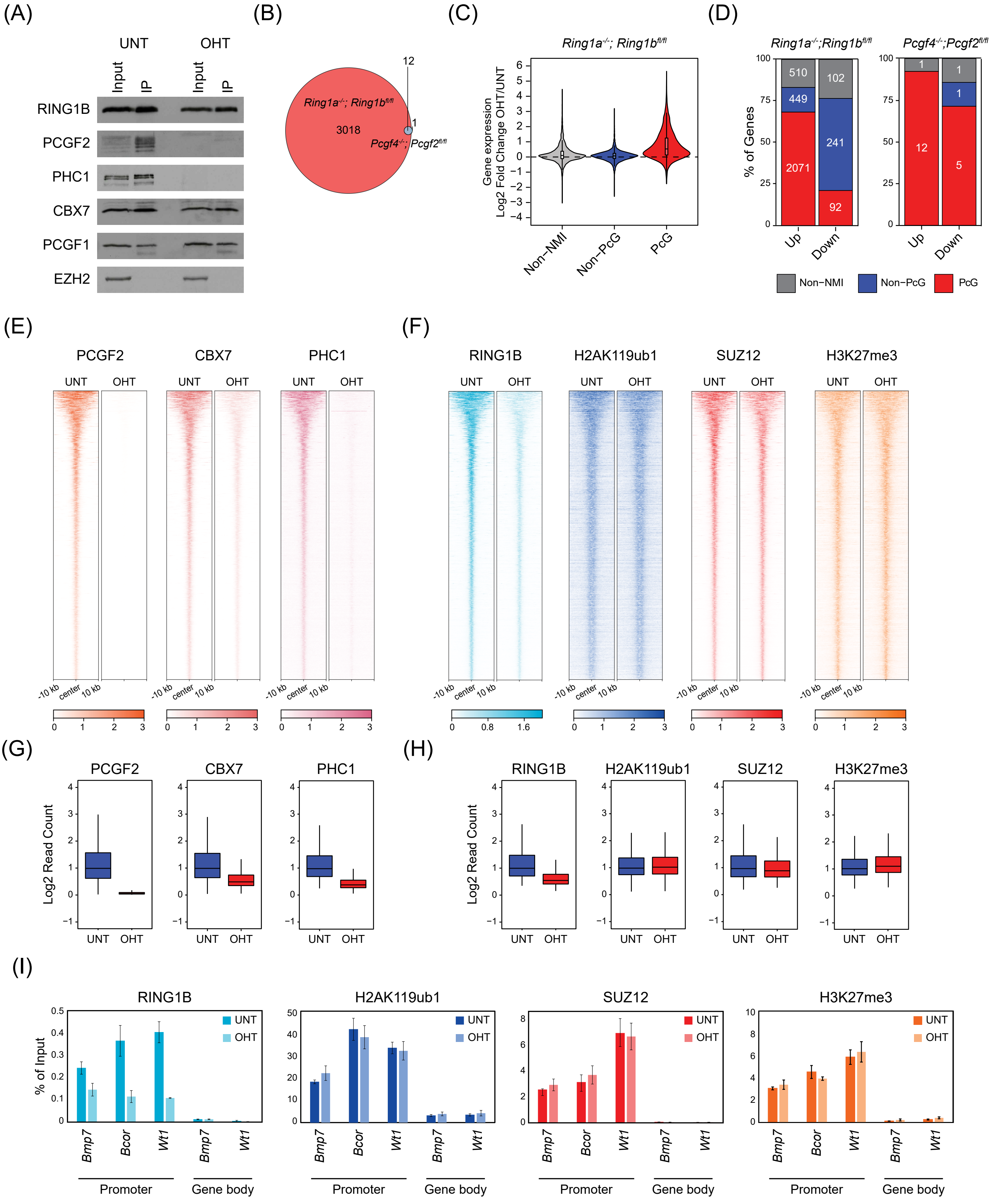
(A)
UNT
OHT
Input
IP
Input
IP
RING1B
PCGF2
PHC1
CBX7
PCGF1
EZH2
(B)
12
Ring1a-/-; Ring1bfl/fl
3018
1
Pcgf4-/-; Pcgf2fl/fl
(C)
Ring1a-/-; Ring1bfl/fl
Gene expression
Log2 Fold Change OHT/UNT
Non−NMI
Non−PcG
PcG
(D)
Ring1a-/-;Ring1bfl/fl
Pcgf4-/-;Pcgf2fl/fl
% of Genes
510
449
2071
102
241
92
1
12
1
1
5
Up
Down
Non−NMI
Non−PcG
PcG
(E)
PCGF2
CBX7
PHC1
UNT
OHT
-10 kb
center
10 kb
(F)
RING1B
H2AK119ub1
SUZ12
H3K27me3
(G)
Log2 Read Count
(H)
(I)
% of Input
Bmp7
Bcor
Wt1
Promoter
Gene body

Figure S1

Figure S1. Related to Figure 1.

- **(A)** Input and immunoprecipitation of RING1B from *Pcgf4*$^{-/-}$*;Pcgf2*$^{fl/fl}$ ESCs (UNT and OHT-treated) followed by western blot with antibodies against PCGF2, PHC1 and CBX7 (canonical PRC1-specific subunits), PCGF1 (a variant PRC1-specific factor) and EZH2 (a PRC2 component that does not interact with RING1B). In OHT-treated cells, PHC1 and PCGF2 no longer associate with RING1B, indicating canonical PRC1 has been disrupted.
- **(B)** A Venn diagram of significantly upregulated genes (p-adj < 0.05 and > 1.5-fold) following OHT treatment in *Ring1a*$^{-/-}$*;Ring1b*$^{fl/fl}$ and *Pcgf4*$^{-/-}$*;Pcgf2*$^{fl/fl}$ ESCs.
- **(C)** A violin plot comparing log2-fold expression changes in *Ring1a*$^{-/-}$*;Ring1b*$^{fl/fl}$ ESCs for three classes of genes - Non-NMI (n = 5867), Non-PcG-occupied (n = 9898) and PcG-occupied (n = 4868). Non-NMI genes lack a non-methylated CpG island (NMI) at their promoter. PcG- and Non-PcG-occupied genes have an NMI promoter that is either bound or not bound by Polycomb complexes.
- **(D)** Bar plots of the distribution of gene expression changes (p-adj < 0.05 and > 1.5-fold) in *Ring1a*$^{-/-}$*;Ring1b*$^{fl/fl}$ and *Pcgf4*$^{-/-}$*;Pcgf2*$^{fl/fl}$ ESCs following OHT treatment between different classes of genes described in (C).
- **(E)** Heatmaps of PCGF2, CBX7 and PHC1 cChIP-seq in *Pcgf4*$^{-/-}$*;Pcgf2*$^{fl/fl}$ ESCs (UNT and OHT) at classical Polycomb chromatin domains sorted based on RING1B occupancy in untreated cells.
- **(F)** As in (E) for RING1B, H2AK119ub1, SUZ12 and H3K27me3.
- **(G)** Box plots of PCGF2, CBX7 and PHC1 cChIP-seq signal at classical Polycomb chromatin domains in *Pcgf4*$^{-/-}$*; Pcgf2*$^{fl/fl}$ ESCs before (UNT, blue) and after OHT treatment (OHT, red).
- **(H)** As in (G) for RING1B, H2AK119ub1, SUZ12 and H3K27me3.
- **(I)** ChIP-qPCR analysis of RING1B, H2AK119ub1, SUZ12 and H3K27me3 in *Pcgf4*$^{-/-}$*;Pcgf2*$^{fl/fl}$ ESCs (UNT and OHT) for a panel of Polycomb-bound genes. Error bars show SEM (n=2 for RING1B and n=3 for H2AK119ub1, SUZ12 and H3K27me3).

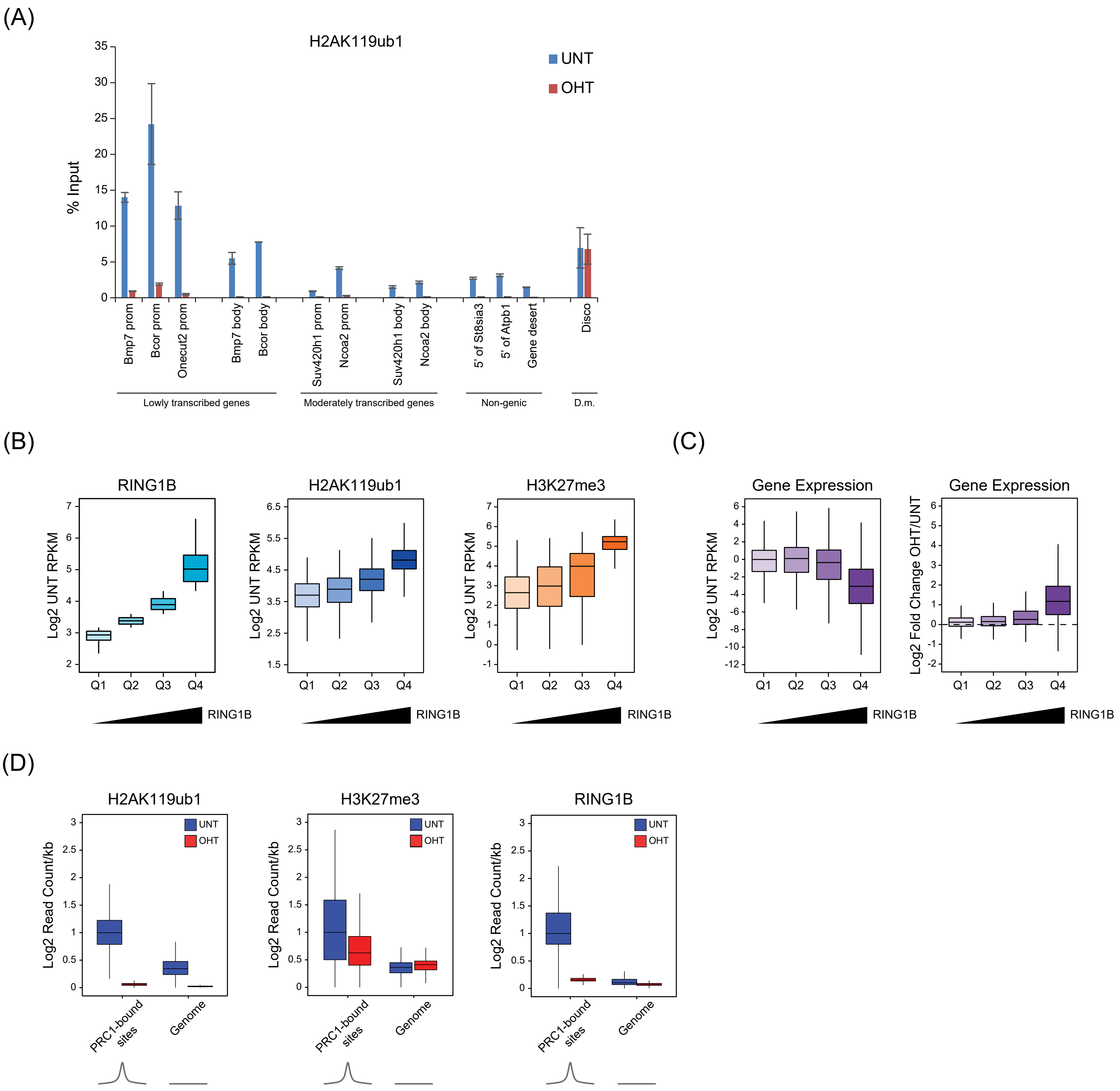
(A)
H2AK119ub1
UNT
OHT
% Input
Bmp7 prom
Bcor prom
Onecut2 prom
Bmp7 body
Bcor body
Suv420h1 prom
Ncoa2 prom
Suv420h1 body
Ncoa2 body
5' of St8sia3
5' of Atpb1
Gene desert
Disco
Lowly transcribed genes
Moderately transcribed genes
Non-genic
D.m.
(B)
RING1B
H2AK119ub1
H3K27me3
Log2 UNT RPKM
Q1
Q2
Q3
Q4
RING1B
(C)
Gene Expression
Log2 UNT RPKM
Log2 Fold Change OHT/UNT
(D)
H2AK119ub1
H3K27me3
RING1B
Log2 Read Count/kb
UNT
OHT
PRC1-bound sites
Genome

Figure S2

Figure S2. Related to Figure 2.

- **(A)** ChIP-qPCR analysis of H2AK119ub1 in untreated (UNT, blue) and OHT-treated (OHT, red) *Ring1a*$^{-/-}$;*Ring1b*$^{fl/fl}$ ESCs. To illustrate the broad nature of H2AK119ub1 in mouse ESCs, qPCR was performed using primers at various genic (lowly or moderately transcribed) or non-genic regions as indicated. As a control for ChIP efficiency, all ChIPs included Drosophila SG4 cells, which were interrogated using primers specific for a Polycomb target in *Drosophila melanogaster* (D.m.).
- **(B)** Box plots of RING1B, H2AK119ub1 and H3K27me3 cChIP-seq signal in untreated *Ring1a*$^{-/-}$; *Ring1b*$^{fl/fl}$ ESCs at PRC1-bound gene promoters divided into quartiles based on RING1B occupancy (from lowest Q1 to highest Q4).
- **(C)** Box plots of gene expression in untreated cells (left panel) and log2-fold expression changes following OHT treatment (right panel) in *Ring1a*$^{-/-}$;*Ring1b*$^{fl/fl}$ ESCs for the gene quartiles as in (B).
- **(D)** Box plots of H2AK119ub1, H3K27me3 and RING1B cChIP-seq signal at PRC1-bound sites and 100 kb windows covering the genome in *Ring1a*$^{-/-}$;*Ring1b*$^{fl/fl}$ ESCs (UNT, blue, and OHT, red). This analysis shows that there is an appreciable amount of H2AK119ub1 outside of PRC1-bound sites throughout the genome which is lost after removal of RING1A/B. In addition, it demonstrates that PRC1 loss leads to a specific reduction in H3K27me3 at regions with punctate H2AK119ub1, indicating that communication between PRC1 and PRC2 is largely restricted to these regions of the genome.

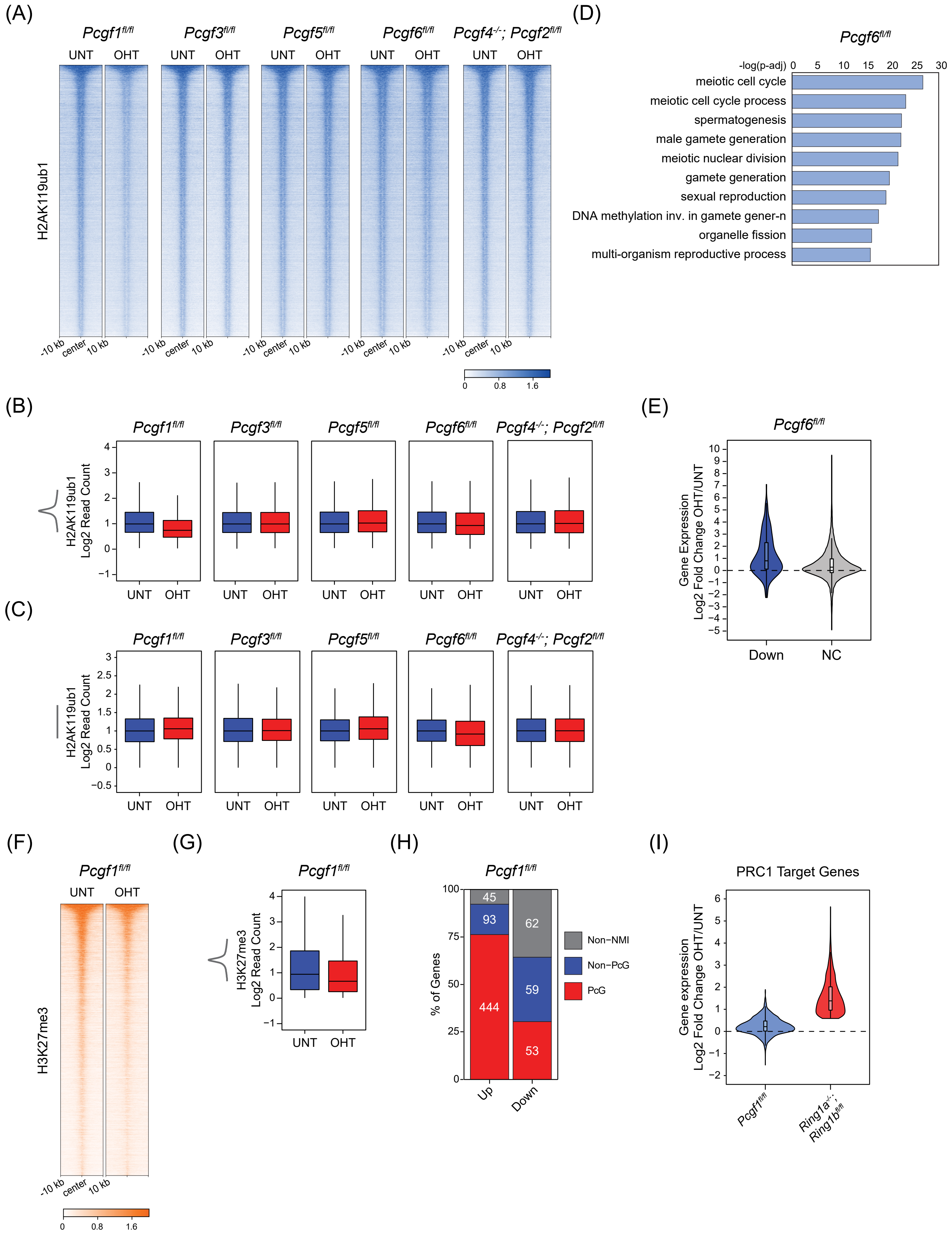

(A)
Pcgf1fl/fl
Pcgf3fl/fl
Pcgf5fl/fl
Pcgf6fl/fl
Pcgf4-/-; Pcgf2fl/fl
UNT OHT
H2AK119ub1
-10 kb center 10 kb
0 0.8 1.6
(D)
Pcgf6fl/fl
-log(p-adj) 0 5 10 15 20 25 30
meiotic cell cycle
meiotic cell cycle process
spermatogenesis
male gamete generation
meiotic nuclear division
gamete generation
sexual reproduction
DNA methylation inv. in gamete gener-n
organelle fission
multi-organism reproductive process
(B)
H2AK119ub1 Log2 Read Count
UNT OHT
(C)
H2AK119ub1 Log2 Read Count
(E)
Gene Expression Log2 Fold Change OHT/UNT
Down
NC
(F)
H3K27me3
(G)
H3K27me3 Log2 Read Count
(H)
% of Genes
45
93
444
62
59
53
Up
Down
Non−NMI
Non−PcG
PcG
(I)
PRC1 Target Genes
Gene expression Log2 Fold Change OHT/UNT
Pcgf1fl/fl
Ring1a-/-; Ring1bfl/fl

Figure S3

Figure S3. Related to Figure 3.

- **(A)** Heatmaps of H2AK119ub1 cChIP-seq in *Pcgf1*$^{fl/fl}$, *Pcgf3*$^{fl/fl}$, *Pcgf5*$^{fl/fl}$, *Pcgf6*$^{fl/fl}$ and *Pcgf4*$^{-/-}$*; Pcgf2*$^{fl/fl}$ ESCs (UNT and OHT) at PRC1-bound sites sorted based on RING1B occupancy in untreated *Ring1a*$^{-/-}$*;Ring1b*$^{fl/fl}$ ESCs.
- **(B)** Box plots of H2AK119ub1 cChIP-seq at PRC1-bound sites in *Pcgf1*$^{fl/fl}$, *Pcgf3*$^{fl/fl}$, *Pcgf5*$^{fl/fl}$, *Pcgf6*$^{fl/fl}$ and *Pcgf4*$^{-/-}$*;Pcgf2*$^{fl/fl}$ ESCs before (UNT, blue) and after OHT treatment (OHT, red).
- **(C)** Box plots of H2AK119ub1 cChIP-seq at 100 kb windows covering the genome in *Pcgf1*$^{fl/fl}$, *Pcgf3*$^{fl/fl}$, *Pcgf5*$^{fl/fl}$, *Pcgf6*$^{fl/fl}$ and *Pcgf4*$^{-/-}$*;Pcgf2*$^{fl/fl}$ ESCs (UNT and OHT).
- **(D)** A gene ontology analysis of Biological Process term enrichment for genes showing statistically significant reductions in H2AK119ub1 levels at their promoters in *Pcgf6*$^{fl/fl}$ ESCs following OHT treatment (n = 241).
- **(E)** A violin plot comparing log2-fold expression changes for genes showing a statistically significant reduction (Down) or no change (NC) in H2AK119ub1 levels at their promoters in *Pcgf6*$^{fl/fl}$ ESCs following OHT treatment.
- **(F)** A heatmap of H3K27me3 cChIP-seq signal in *Pcgf1*$^{fl/fl}$ ESCs (UNT and OHT) at PRC1-bound sites sorted based on RING1B occupancy in untreated *Ring1a*$^{-/-}$*;Ring1b*$^{fl/fl}$ ESCs.
- **(G)** A box plot of H3K27me3 cChIP-seq signal at PRC1-bound sites in *Pcgf1*$^{fl/fl}$ ESCs before (UNT, blue) and after OHT treatment (OHT, red).
- **(H)** A bar plot of the distribution of gene expression changes (p-adj < 0.05 and > 1.5-fold) in *Pcgf1*$^{fl/fl}$ ESCs following OHT treatment between different classes of genes (Non-NMI, Non-PcG, PcG) described in Figure S1C.
- **(I)** A violin plot comparing log2-fold expression changes of PRC1 target genes in *Pcgf1*$^{fl/fl}$ and *Ring1a*$^{-/-}$*;Ring1b*$^{fl/fl}$ ESCs following OHT treatment.

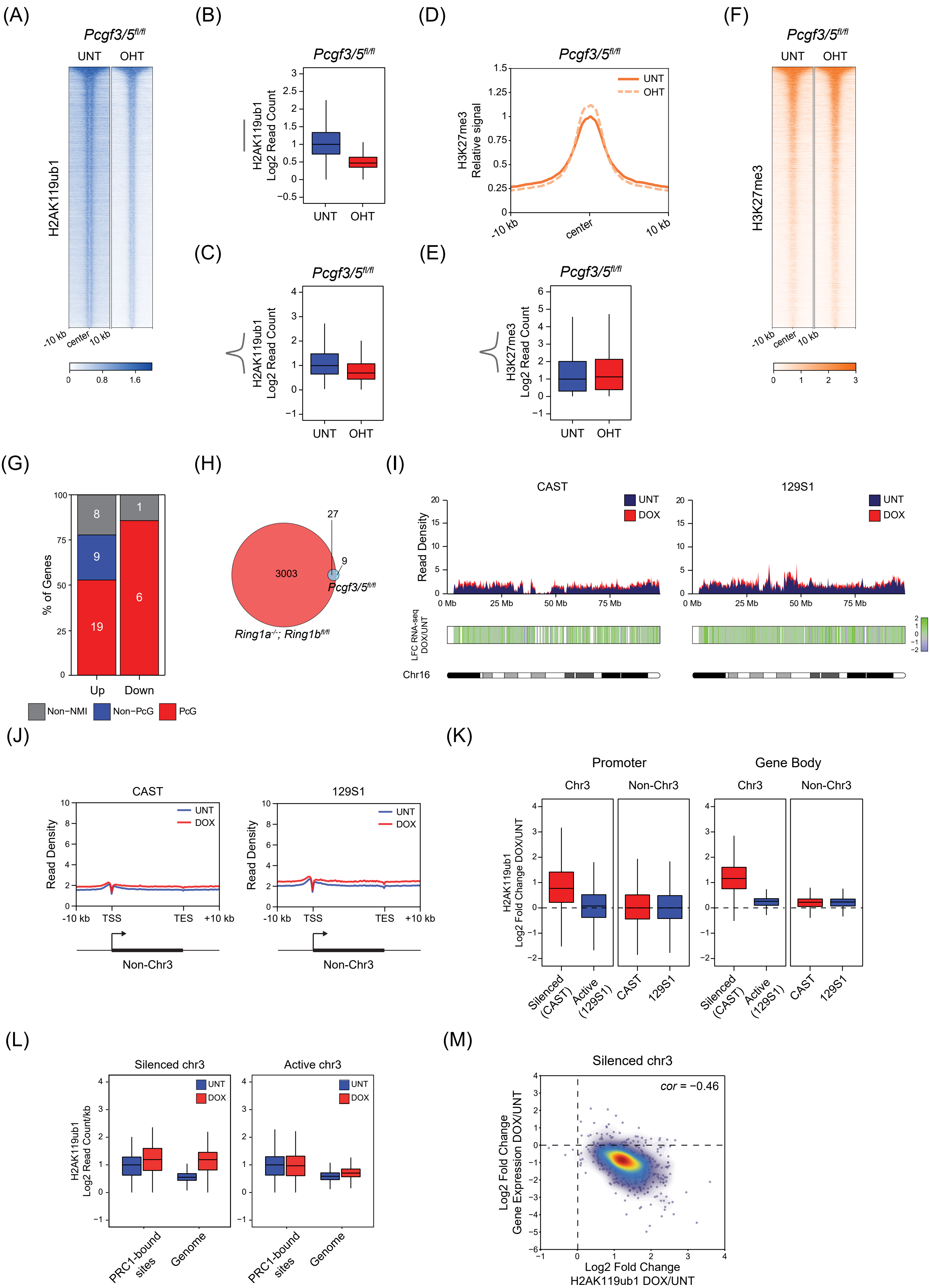

(A)
Pcgf3/5fl/fl
UNT
OHT
H2AK119ub1
-10 kb
center
10 kb
0
0.8
1.6
(B)
Pcgf3/5fl/fl
H2AK119ub1
Log2 Read Count
UNT
OHT
(C)
Pcgf3/5fl/fl
H2AK119ub1
Log2 Read Count
UNT
OHT
(D)
Pcgf3/5fl/fl
H3K27me3
Relative signal
UNT
OHT
-10 kb
center
10 kb
(E)
Pcgf3/5fl/fl
H3K27me3
Log2 Read Count
UNT
OHT
(F)
Pcgf3/5fl/fl
UNT
OHT
H3K27me3
-10 kb
center
10 kb
(G)
% of Genes
Up
Down
Non–NMI
Non–PcG
PcG
(H)
3003
27
9
Pcgf3/5fl/fl
Ring1a-/-; Ring1bfl/fl
(I)
CAST
129S1
UNT
DOX
Read Density
0 Mb
25 Mb
50 Mb
75 Mb
LFC RNA-seq
DOX/UNT
Chr16
(J)
CAST
129S1
UNT
DOX
Read Density
-10 kb
TSS
TES
+10 kb
Non-Chr3
(K)
Promoter
Gene Body
Chr3
Non-Chr3
H2AK119ub1
Log2 Fold Change DOX/UNT
Silenced (CAST)
Active (129S1)
CAST
129S1
(L)
Silenced chr3
Active chr3
UNT
DOX
H2AK119ub1
Log2 Read Count/kb
PRC1-bound sites
Genome
(M)
Silenced chr3
cor = −0.46
Log2 Fold Change
Gene Expression DOX/UNT
Log2 Fold Change
H2AK119ub1 DOX/UNT

Figure S4

Figure S4. Related to Figure 4.

- **(A)** A heatmap of H2AK119ub1 cChIP-seq in *Pcgf3/5$^{fl/fl}$* ESCs (UNT and OHT) at PRC1-bound sites sorted based on RING1B occupancy in untreated *Ring1a$^{-/-}$;Ring1b$^{fl/fl}$* ESCs.
- **(B)** A box plot of H2AK119ub1 cChIP-seq signal at 100 kb windows covering the genome in *Pcgf3/5$^{fl/fl}$* ESCs before (UNT, blue) and after OHT treatment (OHT, red).
- **(C)** A box plot of H2AK119ub1 cChIP-seq signal at PRC1-bound sites in *Pcgf3/5$^{fl/fl}$* ESCs before (UNT, blue) and after OHT treatment (OHT, red).
- **(D)** A metaplot of H3K27me3 cChIP-seq signal at PRC1-bound sites in *Pcgf3/5$^{fl/fl}$* ESCs (UNT and OHT).
- **(E)** A box plot of H3K27me3 cChIP-seq signal at PRC1-bound sites in *Pcgf3/5$^{fl/fl}$* ESCs before (UNT, blue) and after OHT treatment (OHT, red).
- **(F)** A heatmap of H3K27me3 cChIP-seq in *Pcgf3/5$^{fl/fl}$* ESCs (UNT and OHT) at PRC1-bound sites sorted based on RING1B occupancy in untreated *Ring1a$^{-/-}$;Ring1b$^{fl/fl}$* ESCs.
- **(G)** A bar plot of the distribution of gene expression changes (p-adj < 0.05 and > 1.5-fold) in *Pcgf3/5$^{fl/fl}$* ESCs following OHT treatment between different classes of genes (Non-NMI, Non-PcG, PcG) described in Figure S1C.
- **(H)** A Venn diagram of significantly upregulated genes (p-adj < 0.05 and > 1.5-fold) in *Pcgf3/5$^{fl/fl}$* and *Ring1a$^{-/-}$;Ring1b$^{fl/fl}$* ESCs following OHT treatment.
- **(I)** Allele-specific chromosome density plots for H2AK119ub1 cChIP-seq across a control chromosome lacking the *Xist* transgene (chromosome 16) in the *Mus domesticus* (129S1) x *Mus castaneus* F1 hybrid ESCs (UNT and DOX). Below is a heatmap of corresponding log2-fold gene expression changes upon DOX addition. The bins containing no expressed genes are shown in white.
- **(J)** Allele-specific metaplots of H2AK119ub1 cChIP-seq for genes located on chromosomes other than chromosome 3 in the *Mus domesticus* (129S1) x *Mus castaneus* F1 hybrid ESCs (UNT and DOX). Genes were scaled to the same length and aligned at their TSS and TES.
- **(K)** Allele-specific box plots of log2-fold changes in H2AK119ub1 cChIP-seq following DOX treatment at promoters (TSS ± 500 bp) or bodies of genes located on chromosome 3 (Chr3) or any other chromosome (Non-Chr3) in the *Mus domesticus* (129S1) x *Mus castaneus* F1 hybrid ESCs.
- **(L)** Allele-specific box plots of H2AK119ub1 cChIP-seq signal at PRC1-bound sites and 100 kb windows covering the chromosome for the silenced (left, from *Mus castaneus*) or active (right, from *Mus domesticus*) allele of chromosome 3 in the *Mus domesticus* (129S1) x *Mus castaneus* F1 hybrid ESCs (UNT and DOX).
- **(M)** A scatterplot comparing log2-fold changes in H2AK119ub1 and gene expression for all expressed 250 kb genomic bins across the silenced allele of chromosome 3 following induction of *Xist* transgene expression with DOX. *Cor* denotes a Pearson correlation coefficient.

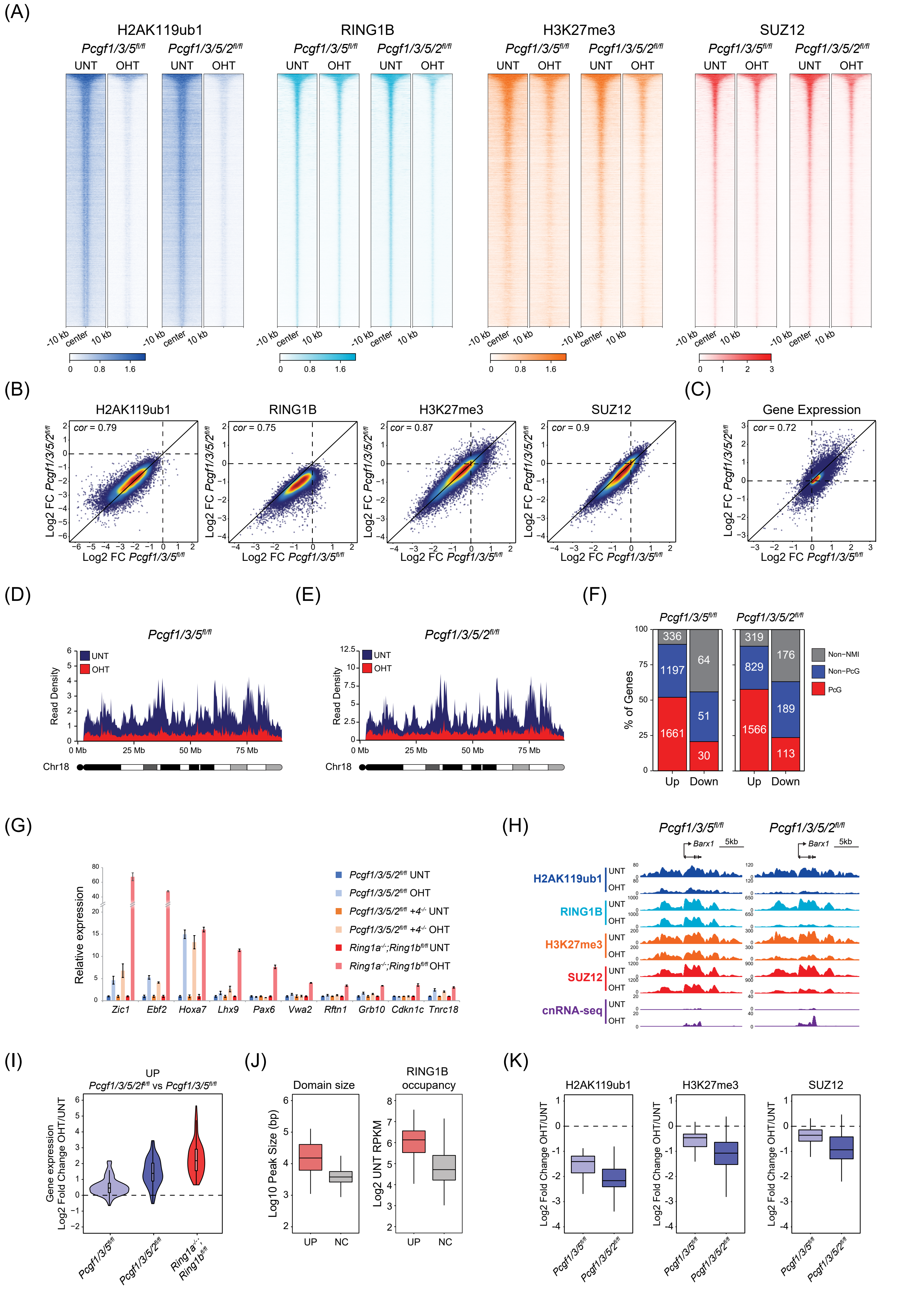
(A)
H2AK119ub1
RING1B
H3K27me3
SUZ12
UNT
OHT
(B)
(C)
Gene Expression
(D)
(E)
(F)
(G)
(H)
(I)
(J)
Domain size
RING1B occupancy
(K)

Figure S5

Figure S5. Related to Figure 5.

- **(A)** Heatmaps of H2AK119ub1, RING1B, H3K27me3 and SUZ12 cChIP-seq in *Pcgf1/3/5*$^{fl/fl}$ (*left*) and *Pcgf1/3/5/2*$^{fl/fl}$ (*right*) ESCs (UNT and OHT) at PRC1-bound sites sorted based on RING1B occupancy in untreated *Ring1a*$^{-/-}$*;Ring1b*$^{fl/fl}$ ESCs.
- **(B)** Scatterplots comparing log2-fold changes in H2AK119ub1, RING1B, H3K27me3 and SUZ12 cChIP-seq at PRC1-bound sites between *Pcgf1/3/5*$^{fl/fl}$ and *Pcgf1/3/5/2*$^{fl/fl}$ ESCs following OHT treatment. *Cor* denotes a Pearson correlation coefficient.
- **(C)** A scatterplot comparing log2-fold changes in gene expression (cnRNA-seq) between *Pcgf1/3/5*$^{fl/fl}$ and *Pcgf1/3/5/2*$^{fl/fl}$ ESCs following OHT treatment. *Cor* denotes a Pearson correlation coefficient.
- **(D)** A chromosome density plot of H2AK119ub1 cChIP-seq across chromosome 18 in *Pcgf1/3/5*$^{fl/fl}$ ESCs before (UNT, blue) and after OHT treatment (OHT, red).
- **(E)** As in (D) for *Pcgf1/3/5/2*$^{fl/fl}$ ESCs.
- **(F)** A bar plot comparing the distribution of gene expression changes (p-adj < 0.05 and > 1.5-fold) in *Pcgf1/3/5*$^{fl/fl}$ (left) and *Pcgf1/3/5/2*$^{fl/fl}$ (right) ESCs following OHT treatment between different classes of genes (Non-NMI, Non-PcG, PcG) described in Figure S1C.
- **(G)** RT-qPCR analysis of expression for a panel of Polycomb target genes in *Pcgf1/3/5/2*$^{fl/fl}$, *Pcgf4*$^{-/-}$*;Pcgf1/3/5/2*$^{fl/fl}$ and *Ring1a*$^{-/-}$*;Ring1b*$^{fl/fl}$ ESCs (UNT and OHT). Gene expression is normalised to *b-actin* and is shown relative to the average expression in untreated cells for each individual cell line. Error bars show SEM (n=3).
- **(H)** A genomic snapshot of a PRC1 target gene that is further derepressed when PCGF2 is removed in addition to PCGF1/3/5, showing PRC1 (H2AK119ub1 and RING1B) and PRC2 (H3K27me3 and SUZ12) cChIP-seq, and cnRNA-seq in *Pcgf1/3/5*$^{fl/fl}$ and *Pcgf1/3/5/2*$^{fl/fl}$ ESCs (UNT and OHT).
- **(I)** A violin plot comparing log2-fold expression changes for PRC1 target genes that are further derepressed when PCGF2 is removed along with PCGF1/3/5 (n = 137) in *Pcgf1/3/5*$^{fl/fl}$, *Pcgf1/3/5/2*$^{fl/fl}$ and *Ring1a*$^{-/-}$*;Ring1b*$^{fl/fl}$ ESCs following OHT treatment.
- **(J)** Box plots of the size (left panel) and RING1B occupancy in untreated cells (right panel) for Polycomb chromatin domains associated with PRC1 target genes displaying an additional derepression (UP, n=137) or no additional derepression (NC, n = 1934) when PCGF2 is removed along with PCGF1/3/5 in *Pcgf1/3/5/2*$^{fl/fl}$ ESCs.
- **(K)** Box plots comparing log2-fold changes in H2AK119ub1, H3K27me3 and SUZ12 cChIP-seq at promoters of PRC1 target genes that are further derepressed when PCGF2 is removed along with PCGF1/3/5 (n = 137) in *Pcgf1/3/5*$^{fl/fl}$ and *Pcgf1/3/5/2*$^{fl/fl}$ ESCs following OHT treatment.

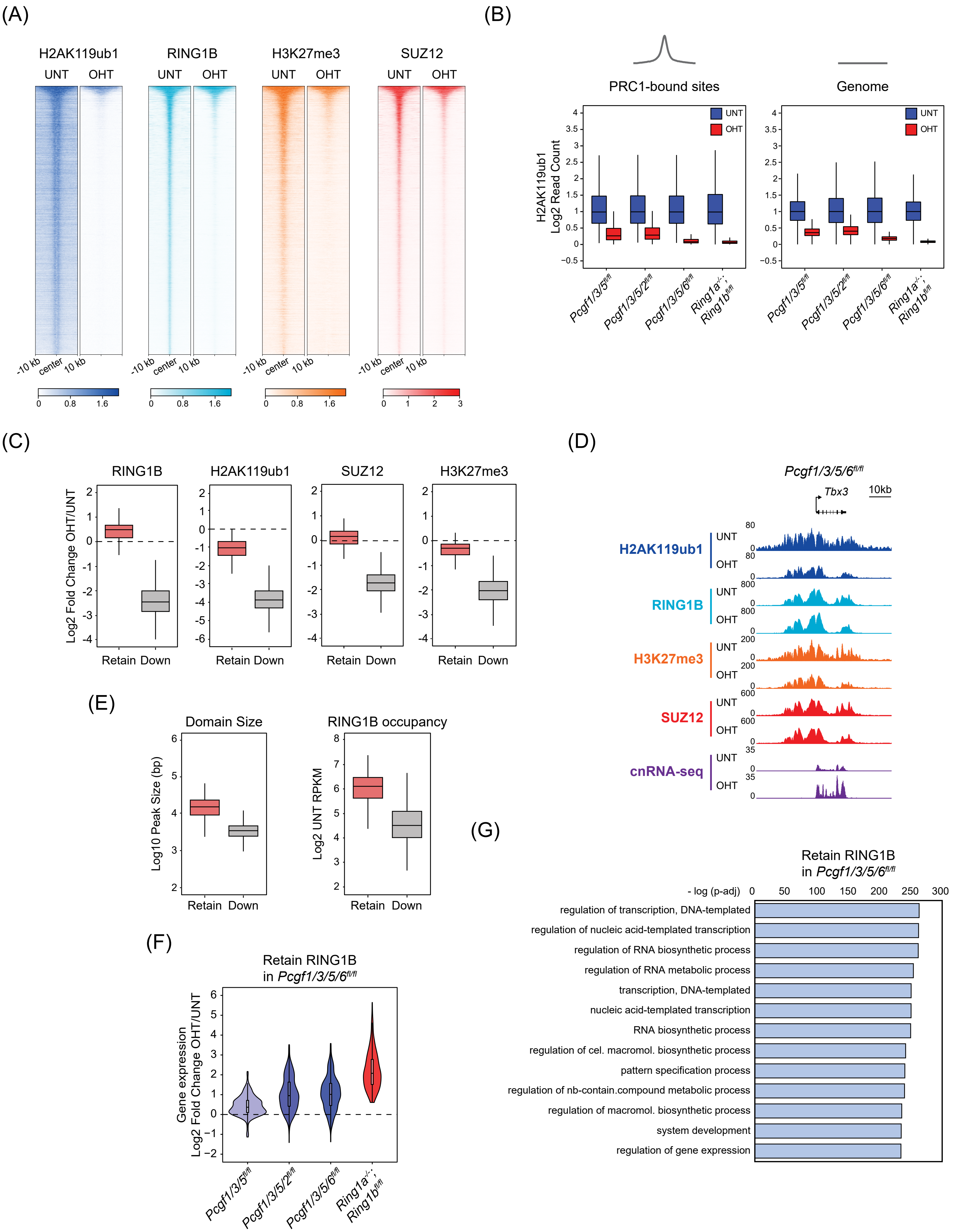
(A)
H2AK119ub1
RING1B
H3K27me3
SUZ12
UNT
OHT
-10 kb
center
10 kb
(B)
PRC1-bound sites
Genome
H2AK119ub1
Log2 Read Count
UNT
OHT
Pcgf1/3/5fl/fl
Pcgf1/3/5/2fl/fl
Pcgf1/3/5/6fl/fl
Ring1a-/-; Ring1bfl/fl
(C)
Log2 Fold Change OHT/UNT
Retain Down
(D)
Pcgf1/3/5/6fl/fl
Tbx3
10kb
H2AK119ub1
RING1B
H3K27me3
SUZ12
cnRNA-seq
(E)
Domain Size
RING1B occupancy
Log10 Peak Size (bp)
Log2 UNT RPKM
Retain Down
(F)
Retain RING1B in Pcgf1/3/5/6fl/fl
Gene expression Log2 Fold Change OHT/UNT
(G)
Retain RING1B in Pcgf1/3/5/6fl/fl
- log (p-adj)
regulation of transcription, DNA-templated
regulation of nucleic acid-templated transcription
regulation of RNA biosynthetic process
regulation of RNA metabolic process
transcription, DNA-templated
nucleic acid-templated transcription
RNA biosynthetic process
regulation of cel. macromol. biosynthetic process
pattern specification process
regulation of nb-contain.compound metabolic process
regulation of macromol. biosynthetic process
system development
regulation of gene expression

Figure S6

Figure S6. Related to Figure 6.

(A) Heatmaps of H2AK119ub1, RING1B, H3K27me3 and SUZ12 cChIP-seq in *Pcgf1/3/5/6*$^{fl/fl}$ ESCs (UNT and OHT) at PRC1-bound sites sorted based on RING1B occupancy in untreated *Ring1a*$^{-/-}$;*Ring1b*$^{fl/fl}$ ESCs.

(B) Box plots of H2AK119ub1 cChIP-seq signal at PRC1-bound sites (left) and 100 kb windows covering the genome (right) in *Pcgf1/3/5*$^{fl/fl}$, *Pcgf1/3/5/2*$^{fl/fl}$, *Pcgf1/3/5/6*$^{fl/fl}$ and *Ring1a*$^{-/-}$; *Ring1b*$^{fl/fl}$ ESCs (UNT, blue, and OHT, red).

(C) Box plots of log2-fold changes in RING1B, H2AK119ub1, SUZ12 and H3K27me3 cChIP-seq at the promoters of PRC1 target genes showing no reduction (Retain, n=354) or a statistically significant decrease (Down, n=1717) in RING1B occupancy in *Pcgf1/3/5/6*$^{fl/fl}$ ESCs following OHT treatment.

(D) A genomic snapshot of a PRC1 target gene that retains RING1B binding at the promoter following removal of PCGF1/3/5/6, showing PRC1 (H2AK119ub1 and RING1B) and PRC2 (H3K27me3 and SUZ12) cChIP-seq, and cnRNA-seq in *Pcgf1/3/5/6*$^{fl/fl}$ ESCs (UNT and OHT).

(E) Box plots of the size (left panel) and RING1B occupancy in untreated cells (right panel) for Polycomb chromatin domains associated with the promoters of PRC1 target genes that demonstrate no reduction (Retain, n=354) or a statistically significant decrease (Down, n=1717) in RING1B occupancy in *Pcgf1/3/5/6*$^{fl/fl}$ ESCs following OHT treatment.

(F) A violin plot of log2-fold expression changes for PRC1 target genes that retain RING1B binding at their promoter upon PCGF1/3/5/6 removal (n=354) in *Pcgf1/3/5*$^{fl/fl}$, *Pcgf1/3/5/2*$^{fl/fl}$, *Pcgf1/3/5/6*$^{fl/fl}$, and *Ring1a*$^{-/-}$;*Ring1b*$^{fl/fl}$ ESCs following OHT treatment.

(G) A gene ontology analysis of Biological Process term enrichment for genes that retain RING1B in *Pcgf1/3/5/6*$^{fl/fl}$ ESCs following OHT treatment (n = 354).

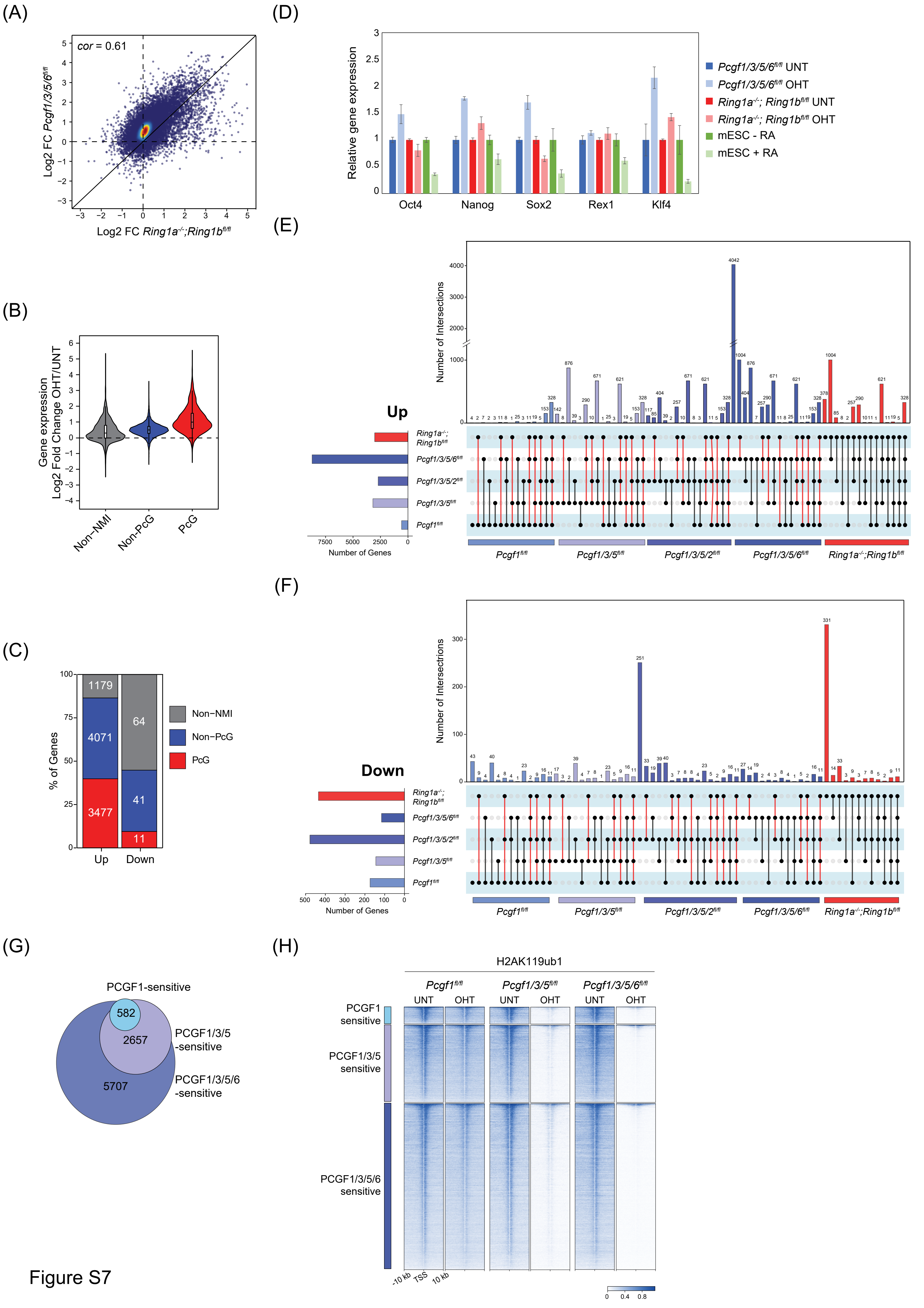
(A)
cor = 0.61
Log2 FC Pcgf1/3/5/6fl/fl
Log2 FC Ring1a-/-;Ring1bfl/fl
(B)
Gene expression
Log2 Fold Change OHT/UNT
Non−NMI
Non−PcG
PcG
(C)
% of Genes
1179
4071
3477
64
41
11
Up
Down
Non−NMI
Non−PcG
PcG
(D)
Relative gene expression
Oct4
Nanog
Sox2
Rex1
Klf4
Pcgf1/3/5/6fl/fl UNT
Pcgf1/3/5/6fl/fl OHT
Ring1a-/-; Ring1bfl/fl UNT
Ring1a-/-; Ring1bfl/fl OHT
mESC - RA
mESC + RA
(E)
Up
Number of Intersections
Number of Genes
Ring1a-/-; Ring1bfl/fl
Pcgf1/3/5/6fl/fl
Pcgf1/3/5/2fl/fl
Pcgf1/3/5fl/fl
Pcgf1fl/fl
(F)
Down
Number of Intersectrions
Number of Genes
Ring1a-/-; Ring1bfl/fl
Pcgf1/3/5/6fl/fl
Pcgf1/3/5/2fl/fl
Pcgf1/3/5fl/fl
Pcgf1fl/fl
(G)
PCGF1-sensitive
582
2657
5707
PCGF1/3/5 -sensitive
PCGF1/3/5/6 -sensitive
(H)
H2AK119ub1
Pcgf1fl/fl
Pcgf1/3/5fl/fl
Pcgf1/3/5/6fl/fl
UNT
OHT
PCGF1 sensitive
PCGF1/3/5 sensitive
PCGF1/3/5/6 sensitive
-10 kb
TSS
10 kb

Figure S7

Figure S7. Related to Figure 7.

(A) A scatterplot comparing log2-fold changes in gene expression in *Pcgf1/3/5/6*$^{fl/fl}$ and *Ring1a*$^{-/-}$*; Ring1b*$^{fl/fl}$ ESCs following OHT treatment. *Cor* denotes a Pearson correlation coefficient.

(B) A violin plot of log2-fold gene expression changes in *Pcgf1/3/5/6*$^{fl/fl}$ ESCs following OHT treatment for different classes of genes (Non-NMI, Non-PcG and PcG) described in Figure S1C.

(C) A bar plot of the distribution of gene expression changes (p-adj < 0.05 and > 1.5-fold) in *Pcgf1/3/5/6*$^{fl/fl}$ ESCs following OHT treatment between different classes of genes (Non-NMI, Non-PcG, PcG) as described in Figure S1C.

(D) Expression of pluripotency-associated genes in *Pcgf1/3/5/6*$^{fl/fl}$ and *Ring1a*$^{-/-}$*; Ring1b*$^{fl/fl}$ ESCs (UNT and OHT) as well as in ESCs before (-RA) and after 72 hr retinoic acid treatment (+RA) to induce differentiation. Read counts from cnRNA-seq (*Pcgf1/3/5/6*$^{fl/fl}$ and *Ring1a*$^{-/-}$*; Ring1b*$^{fl/fl}$) or 4sU RNA-seq (RA-treated ESCs (Dimitrova et al., 2018)) were normalised to the average gene expression in the corresponding untreated cells. Error bars show SEM (n=3).

(E) UpSet plot for significantly upregulated genes in *Pcgf1*$^{fl/fl}$, *Pcgf1/3/5*$^{fl/fl}$, *Pcgf1/3/5/2*$^{fl/fl}$, *Pcgf1/3/5/6*$^{fl/fl}$, and *Ring1a*$^{-/-}$*; Ring1b*$^{fl/fl}$ ESCs following OHT treatment. The number of genes in each set is shown in the bar chart on the left. All non-empty intersections for each set were grouped together and sorted by the number of overlapping sets. Intersections of each set of genes with the set of genes upregulated in *Ring1a*$^{-/-}$*; Ring1b*$^{fl/fl}$ ESCs are highlighted in red.

(F) As in (E) for significantly downregulated genes (p-adj < 0.05 and > 1.5-fold).

(G) A Venn diagram illustrating the classification of variant PRC1-regulated genes used for the heatmaps in Figure 7E and Figure S7H. PCGF1-sensitive genes were defined as genes significantly upregulated following OHT treatment in *Pcgf1*$^{fl/fl}$ ESCs. PCGF1/3/5-sensitive genes were defined as the set of genes significantly upregulated following OHT treatment in *Pcgf1/3/5*$^{fl/fl}$ ESCs, excluding the PCGF1-sensitive set of genes. Similarly, PCGF1/3/5/6-sensitive genes were defined as the set of genes significantly upregulated following OHT treatment in *Pcgf1/3/5/6*$^{fl/fl}$ ESCs, excluding the PCGF1- and PCGF1/3/5-sensitive sets of genes.

(H) Heatmaps of H2AK119ub1 cChIP-seq in *Pcgf1*$^{fl/fl}$, *Pcgf1/3/5*$^{fl/fl}$, and *Pcgf1/3/5/6*$^{fl/fl}$ ESCs (UNT and OHT) at promoters of variant PRC1-regulated genes. Classification of genes into PCGF1-, PCGF1/3/5- and PCGF1/3/5/6- sensitive is as in (G).
